# Optimal Non-Pharmacological Interventions for Reducing Problematic Internet Use in Youth: A Systematic Review and Bayesian Network Meta-Analysis

**DOI:** 10.3390/bs15010098

**Published:** 2025-01-20

**Authors:** Jing-Jing Tian, Xiao-Ya He, Zhen Guo

**Affiliations:** 1 Department of Physical Education, Tsinghua University, Haidian District, Beijing 100190, China; tjj@mail.tsinghua.edu.cn; 2 Sports & Medicine Integrative Innovation Center, Capital University of Physical Education and Sports, No. 11, North Third Ring West Road, Haidian District, Beijing 100190, China; hexiaoya2023@cupes.edu.cn

**Keywords:** non-pharmacological interventions, internet addiction, problematic internet use, youth, network meta-analysis

## Abstract

The purpose of this network meta-analysis (NMA) is to compare the effect of different non-pharmacological interventions (NPIs) on Problematic Internet Use (PIU). Randomized controlled trials (RCTs) published from their inception to 22 December 2023 were searched in Cochrane Central Register of Controlled Trials, Embase, Medline, Web of Science, China National Knowledge Infrastructure, China Science and Technology Journal Database, Chinese BioMedical Literature Database, and WanFang Data. We carried out a data analysis to compare the efficacy of various NPIs using Bayesian NMA. A battery of analyses and assessments, such as conventional meta-analysis and risk of bias, were performed concurrently. Two reviewers extracted data and evaluated bias using the Cochrane Risk of Bias tool independently. We identified 90 RCTs including 15 different NPIs (5986 participants), namely sports intervention (SI), electroencephalogram biological feedback (EBF), reality therapy (RT), positive psychology therapy (PPT), sandplay therapy (ST), educational intervention (EI), compound psychotherapy (CPT), electroacupuncture therapy (AT), group counseling (GC), family therapy (FT), electrotherapy (ELT), craving behavior intervention (CBI), virtual reality therapy (VRT), cognitive behavior therapy (CBT), and mindfulness therapy (MT). Our NMA results showed that SI, EBF, RT, PPT, ST, EI, CPT, AT, GC, FT, ELT, CBT, CBI, VRT, and MT were effective in reducing PIU levels. The most effective NPI was SI (SMD = −4.66, CrI: −5.51, −3.82, SUCRA = 95.43%), followed by EBF (SMD = −4.51, CrI: −6.62, −2.39, SUCRA = 90.89%) and RT (SMD = −3.83, CrI: −6.01, −1.62, SUCRA = 81.90%). Our study showed that SI was the best NPI to relieve PIU levels in youth. Medical staff should be aware of the application of SI to the treatment of PIU in youth in future clinical care.

## 1. Introduction

Epidemiological surveys indicate that approximately 49.7% of the global population uses the internet daily (Lozano-Blasco et al., 2022). As the number of internet users increases, Problematic Internet Use (PIU) has emerged as one of the forms of addiction-related behaviors. PIU (so-called internet addiction) first characterized by Young (Young, 1996), is recognized as a behavioral disorder in which excessive engagement with the internet leads to disruptions in an individual’s personal and social life (Poon, 2018). PIU includes addictive use of the internet, in both specified and unspecified domains, which is characterized by diminished control over usage and failures to stop/control use despite the occurrence of negative consequences resulting in functional impairments in daily life (Lizarte Simón et al., 2024). “Internet Gaming Disorder” as a subtype of PIU was included in the fifth edition of the Diagnostic and Statistical Manual of Mental Disorders (DSM-5) as a potential new diagnosis in 2013. The World Health Organization (WHO) has also included “Gaming Disorder” as a category of substance use and addictive disorders in the ICD-11 beta draft. This distinction is crucial for medical diagnosis, as it enables professionals to identify and treat specific Internet-related disorders. Furthermore, it assists clinicians in developing targeted treatment plans and interventions.

PIU can lead to many health problems. On a physical level, this includes problems such as visual impairment and sleep disorders. On a psychological level, it can lead to anxiety, depression, and even suicidal tendencies. (Lu et al., 2023; Nakshine et al., 2022). Consequently, PIU is recognized as a significant public health concern, with an average prevalence rate of approximately 14.22% (Meng et al., 2022).

The underlying psychopathological basis of PIU remains a subject of ongoing debate (King et al., 2018); however, substantial research has been conducted on interventions for PIU. Contemporary treatment strategies for PIU can be categorized into two primary types: pharmacological and non-pharmacological interventions (NPIs) (Jiang et al., 2024; Zhang et al., 2022). In contrast to NPIs, pharmacological interventions, such as sertraline and buspirone, exhibit limited efficacy and may pose significant side effects to individuals (Banerjee et al., 2011; Nam et al., 2017). Furthermore, the development of drug resistance following prolonged use is an unavoidable consequence (De Crescenzo et al., 2022). Intervening in adolescent internet addiction through non-pharmacological means can effectively avoid the side effects associated with pharmacological interventions on one hand, and on the other hand, it can significantly reduce the financial burden on families for medical expenses, alleviating the pressure on societal medical costs.

Due to their superior safety profile and reduced side effects compared to pharmacological interventions, NPIs have increasingly attracted the attention of healthcare professionals (Dai et al., 2020). These interventions are defined as therapeutic approaches that do not necessitate the prescription of medications and can be employed either in isolation or in conjunction with other measures (Burton et al., 2021). The primary NPIs currently utilized for addressing PIU can be divided into four categories, namely psychological interventions (e.g., group counseling and cognitive–behavioral therapy), sports interventions, integrated interventions, and electrotherapy. Currently, while there are studies that have examined the effectiveness of various interventions for internet addiction (Zhang et al., 2022; Zhu et al., 2023), none have conducted a meta-analysis focusing specifically on non-pharmacological interventions. There exists only one systematic review indicating that NPIs, viewed from a holistic perspective, can effectively alleviate the symptoms of PIU in adolescents (Jiang et al., 2024).

Despite the general consensus that NPIs are beneficial for adolescents with PIU, there has been limited attention to integrating these major therapeutic interventions and ranking their effectiveness in a hierarchical manner. For example, sports intervention has emerged as a frequently recommended approach in the field of PIU intervention, and its efficacy has been validated by a substantial body of research. Nevertheless, when contrasted with other NPIs methods, such as psychological and family interventions, the relative effectiveness of exercise intervention remains undetermined. Additionally, the specific components of exercise intervention, the optimal intervention duration, and the appropriate exercise dosage remain unclear. Bayesian network meta-analysis (NMA) provides a solution to address these limitations by facilitating the simultaneous comparison and ranking of two or more interventions, even in the absence of direct evidence between them. Unlike paired meta-analysis, network meta-analysis (NMA) improves the precision of efficacy estimates by integrating both direct and indirect treatment effect estimates. This approach offers several significant advantages, including enhanced flexibility, increased stability in small sample sizes, adaptability to complex models, reduced effects of heterogeneity, and improved decision support (Thorlund & Mills, 2012). Therefore, our study aims to determine the most effective NPIs for treating PIU in youth by comprehensively reviewing and analyzing the available data.

## 2. Methods

### 2.1. Literature Research

We conducted a comprehensive search across several databases, including Medline, Web of Science, Embase, the Cochrane Central Register of Controlled Trials, China National Knowledge Infrastructure, China Science and Technology Journal Database, Chinese BioMedical Literature Database, and WanFang Data. This search encompassed the period from the inception of these databases to December 2023. The objective was to identify relevant randomized controlled trials (RCTs) that utilized parallel-group and cross-over designs. Included studies were required to compare non-pharmacological interventions with other interventions or to rigorously compare any two or more treatments with other treatments or with a control group (placebo, inactive group) in the treatment of adolescent internet addiction according to any clinical criteria based on the Cochrane Handbook for Systematic Review of Interventions. (Chandler et al., 2019). We employed Medical Subject Headings (MeSH) in conjunction with text terms and Boolean logical operators to conduct a thorough search on topics related to internet addiction, youth, non-pharmacological intervention approaches, randomized controlled trials, and other pertinent conceptual keywords. There were no restrictions placed on the year or language of the studies included in the search. Additional information can be found in Supplementary File S1 (search strategy).

Relevant papers were examined through bibliographical lists to retrieve prospective data using a recursive method. Additionally, we personally collected records from significant international conferences held between 2000 and 2023 to ensure that no relevant research presented solely in abstract form was overlooked. Following the established process, two separate authors evaluated the publications by reviewing the titles and abstracts of the collected citations during the initial literature search. Simultaneously, duplicate studies were discarded. Full texts were obtained for further assessment based on the selection criteria if the article appeared to be potentially useful. Ultimately, we excluded research that was published solely in an abstract format and lacked accessible data. For studies with missing raw data, we sent an email to the corresponding author to inquire, and if the person refused to provide raw data, we excluded the study. Any inconsistencies that arose during the search strategy and literature selection process were addressed through discussions led by an experienced author. When we express disagreement about an experienced author, the matter is resolved through arbitration. The raw data for the studies included in this paper are available from the original article. This investigation was conducted and documented in accordance with the guidelines set forth in the Preferred Reporting Items for Systematic Reviews and Meta-Analyses (PRISMA) extended statement for systematic reviews that include network meta-analyses in the healthcare field (Hutton et al., 2015). The analyses were conducted using previously published papers, and there was no requirement for ethics approval or patient permission. The registration number is CRD42024610265.

### 2.2. Inclusion and Exclusion Criteria

The researchers in this study employed a modified version of the Cochrane Consumers and Communication Review Group’s data extraction template to collect relevant data from each trial included in the analysis (Chandler et al., 2019). Any inconsistencies in the collected data were resolved through consensus, and when necessary, discrepancies were objectively assessed by a knowledgeable expert from our team.

Further descriptive information regarding the inclusion and exclusion criteria is summarized in Table S1, in accordance with the PICOS selection criteria.

Non-pharmacological treatments involve an interaction between a therapist and a patient. These treatments may occur in a group setting, through face-to-face individual sessions, or via mobile health platforms. The primary objective of these therapies is to prevent internet addiction. The randomized controlled trials RCTs were analyzed and classified into fifteen distinct active treatment groups and two common control groups based on the type of therapy format. The details of each category of non-pharmacological treatment are presented in Table S2.

### 2.3. Outcome Measurement and Quality Appraisal

Based on the predetermined methodology and criteria, we gathered data from relevant studies on several topics, including research design, first author, publication year, interventions, duration, and participant characteristics such as age, sample size, and sex ratio. All non-pharmacological intervention options were examined to evaluate the primary outcome of interest. The outcome measures employed in each study varied and were represented by a range of scales. To determine the change score of PIU symptoms, we retrieved the mean difference and standard deviation (SD) values between the baseline and the most recent observation. All standardized self-reports and observer-rated instruments measuring PIU symptoms were extracted. Subsequently, we determined the effect size within the established hierarchy by utilizing the highest average when multiple instruments were reported. This approach ensured that each condition comparison contributed a single effect size to the analyses (Altman & Bland, 1996).

Utilizing the Cochrane Risk of Bias tool (Chandler et al., 2019), we assessed and categorized the potential for bias in each included study according to predetermined importance criteria. Each study was assigned a risk of bias classification based on the independent evaluations conducted by two authors across seven domains of risk of bias (ROB): high, low, or unclear. These domains included random sequence generation, allocation concealment, blinding of participants and personnel, blinding of outcome assessment, incomplete outcome data, selective reporting, and other biases.

### 2.4. Statistical Analysis

Bayesian network meta-analysis (NMA) has the ability to simultaneously evaluate and compare multiple interventions (Lu & Ades, 2004). The application of complex models allows for greater flexibility and the generation of scientifically robust explanations based on causal relationships. Initially, we performed a traditional pairwise meta-analysis by comparing all available contrasts for each intervention. The *I*^2^ statistic was employed to quantitatively assess the heterogeneity among the studies under consideration. This statistic classifies heterogeneity into three categories: low, when the *I*^2^ value is below 25%; moderate, for *I*^2^ values ranging from 25% to 75%; and high, when the *I*^2^ value exceeds 75% (Melsen et al., 2014). A comparison-adjustment funnel plot was constructed to identify any significant biases, including potential publication bias. The fifteen non-pharmacological intervention approaches were compared simultaneously using a random-effects Bayesian statistical model, resulting in the development of a connected network that integrates both direct and indirect evidence.

The effectiveness of each intervention approach was clearly demonstrated through a network plot, which provided a compact and informative visual representation. Given that the effect sizes pertain to continuous outcome variables, the standardized mean difference (SMD) was meticulously computed for each comparative study (Hedges, 1984). This was achieved by applying the relevant group means and standard deviations obtained from the individual research. To combine the results and assess the level of uncertainty, the pooled SMD and 95% credibility interval (CrI) were calculated. In instances where the existing literature lacked the necessary data (e.g., mean and standard deviation), which are essential for our extraction processes, we employed an alternative analytical approach. This involved calculating various statistical metrics to estimate the standard deviation. We incorporated standard errors, confidence intervals, and other relevant indicators to enhance the robustness and reliability of our analytical results (Follmann et al., 1992).

The assumption of network transitivity is essential in NMA (Salanti, 2012). To evaluate this assumption, we examined the clinical and methodological aspects of all studies included in the analysis, such as patient characteristics and experimental design. We conducted the NMA using non-informative prior distributions and the Markov Chain Monte Carlo (MCMC) model within a Bayesian framework. Furthermore, we utilized the cumulative ranking likelihood curve to evaluate the effects of NPIs. Since the SUCRA (surface under the cumulative ranking curve) values reflect the efficacy of an intervention, higher SUCRA values indicate more effective interventions (Rücker & Schwarzer, 2015). Funnel plots were employed to detect publication bias; any asymmetry in the funnel plot suggested the presence of publication bias (Sterne & Egger, 2001). All analyses were conducted using Stata version 14.0 (Stata Corp., College Station, TX, USA) and OpenBUGS version 3.2.3.

## 3. Results

### 3.1. Baseline Characteristics and ROB Quality of Included Studies

A total of 46,871 articles were identified following an initial database search. Subsequently, 2123 records were removed due to duplication, and an additional 34,760 records were discarded based on an evaluation of their titles and abstracts. Out of the remaining 391 articles, 10 were sourced through manual searches. After a thorough examination of the complete texts, 301 records were eliminated for various reasons: 27 studies were not RCTs, 78 studies did not yield suitable results, and 196 studies failed to disclose their accessible data. Ultimately, 90 studies were included for NMA.

**Figure 1:**
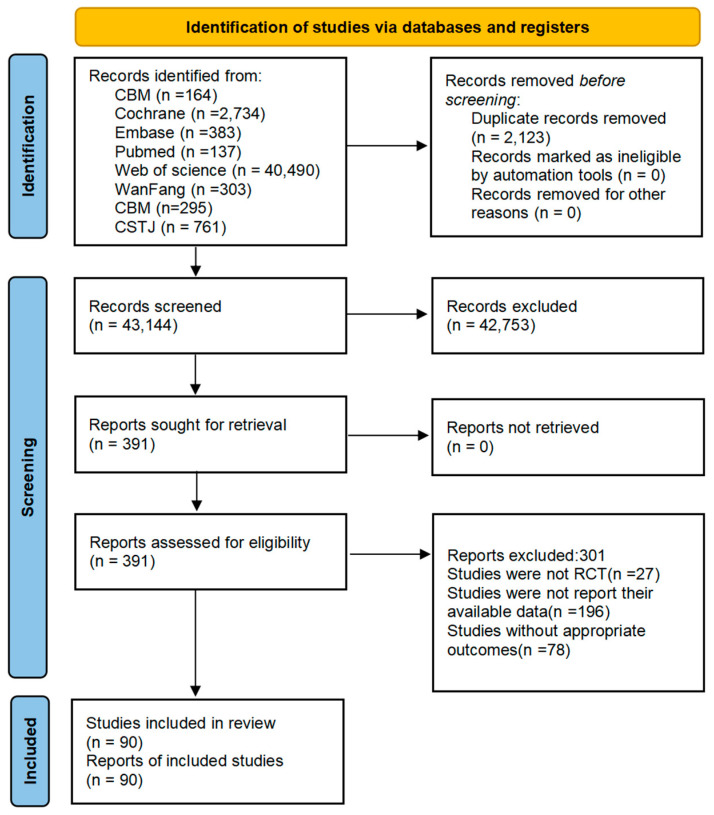
Literature review flowchart. CNKI, China National Knowledge Infrastructure; CMB, Chinese Biomedical; CSTJ, *China Science and Technology Journal*.

Figure 1 presents a flowchart illustrating the PRISMA screening procedure. The characteristics of the research included in this NMA are detailed in Table S3. The analysis encompassed a total of 90 studies, involving a cohort of 5503 individuals aged between 12 and 26 years. The data included in these studies were published between 2007 and 2023. Geographically, 74 studies were conducted in China, 8 in Korea, 5 in Europe, and the remaining 3 in other locations. The overall quality of these studies, as well as the quality of each individual study, is presented in Figures S1 and S2, respectively. Among the 90 studies, 90 were determined to have low bias in the randomized sequence generation criterion, while 49 studies exhibited low bias in the allocation concealment criterion. Of the 20 studies assessed for bias detection, it was found that they demonstrated “high bias.” Additionally, 86 studies were classified as having an “unclear risk of bias.” Based on the attrition items, 7 studies were found to have a high risk of bias, while 19 studies exhibited an uncertain risk of bias. Furthermore, among the other bias items, 15 trials were identified as having a high risk of bias. Overall, the studies included in this research have a low overall risk of bias, which is specifically manifested in a low risk of selection bias and attrition bias, as well as a low risk of reporting bias. However, a high risk of detection bias is an issue present in this paper.

### 3.2. Results of the Pairwise and Network Meta-Analyses

The meta-analysis we conducted showed a high heterogeneity among all the studies included, as indicated by the I-squared values (*I*^2^ = 87.9%, *p* < 0.1). Publication bias is reflected through the funnel plot, which is characterized by the presence of multiple asymmetric scattering points in an inverted funnel shape (Figure S3).

**Figure 2:**
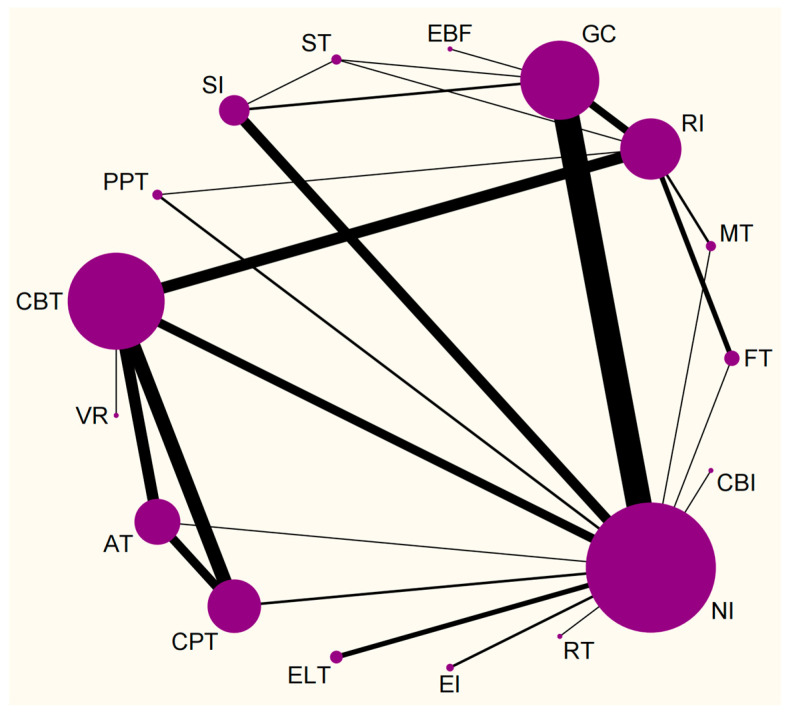
Network meta-analysis of eligible comparisons. AT, Electroacupuncture Therapy; CBT, Cognitive Behavior Therapy; CBI, Craving Behavior Intervention; CPT, Compound Psychotherapy; EBF, Electroencephalogram Biological Feedback; ELT, Electrotherapy; EI, Educational Intervention; FT, Family Therapy; GC, Group Counseling; MT, Mindfulness Therapy; NI, No Intervention; RI, Routine Intervention; RT, Reality Therapy; PPT, Positive Psychology Therapy; ST, Sandplay Therapy; SI, Sports Intervention; VRT, Virtual Reality Therapy.

We constructed a visual representation of a network geometry to showcase each arm, consisting of fifteen NPI strategies and two control circumstances. The most common intervention approach was CBT (Cognitive Behavior Therapy), that investigated in 30 arms (n = 1193), followed by group counseling (GC) (n = 660) and compound psychotherapy (CPT) (n = 462) involving 29 and 13 arms, respectively, then electroacupuncture therapy (AT) (n = 304) and sports intervention (SI) (n = 306) involving 10 arms. The least common interventions were Electroencephalogram Biological Feedback (EBF) (n = 25), Reality Therapy (RT) (n = 13), Craving Behavior Intervention (CBI) (n = 44), Virtual Reality Therapy (VRT) (n = 12) involving 1 arm (Figure 2).

**Figure 3:**
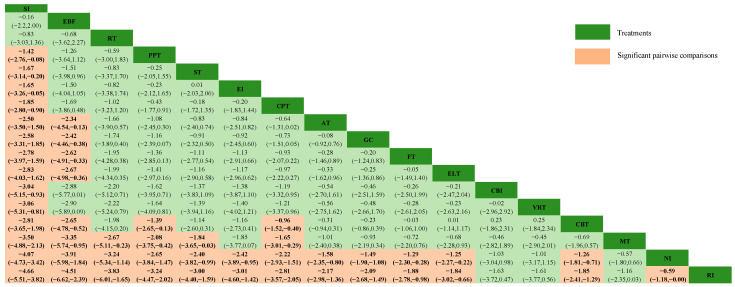
Relative effect sizes of efficacy at post-intervention according to network meta-analysis. AT, Electroacupuncture Therapy; CBT, Cognitive Behavior Therapy; CBI, Craving Behavior Intervention; CPT, Compound Psychotherapy; EBF, Electroencephalogram Biological Feedback; ELT, Electrotherapy; EI, Educational Intervention; FT, Family Therapy; GC, Group Counseling; MT, Mindfulness Therapy; NI, No Intervention; RI, Routine Intervention; RT, Reality Therapy; PPT, Positive Psychology Therapy; ST, Sandplay Therapy; SI, Sports Intervention; VRT, Virtual Reality Therapy.

Figure 3 displays the post-intervention efficacies of different NPI approaches (bold and red represent statistical significance). Twelve intervention approaches were found to be statistically superior to routine intervention (RI), and these included SI (SMD = −4.66, CrI: −5.51, −3.82), EBF (SMD = −4.51, CrI: −6.62, −2.39), RT (SMD = −3.83, CrI: −6.01, −1.65), ST (SMD = −3.00, CrI: −4.40, −1.59), EI (SMD = −3.01, CrI: −4.60, −1.42), CPT (SMD = −2.81, CrI: −3.57, −2.05), AT (SMD = −2.17, CrI: −2.98, −1.36), GC (SMD = −2.09, CrI: −2.68, −1.49), FT (SMD = −1.88, CrI: −2.78, −0.98), ELT (SMD = −1.84, CrI: −3.02, −0.66), CBT (SMD = −1.85, CrI: −2.41, −1.29), NI (SMD = −0.59, and CrI: −1.18, −0.00). However, only SI showed significantly more benefits than CBI [(SMD = −3.04, CrI: −5.15, −0.93) and VRT (SMD = −3.06, CrI: −5.31, −0.81)].

**Figure 4:**
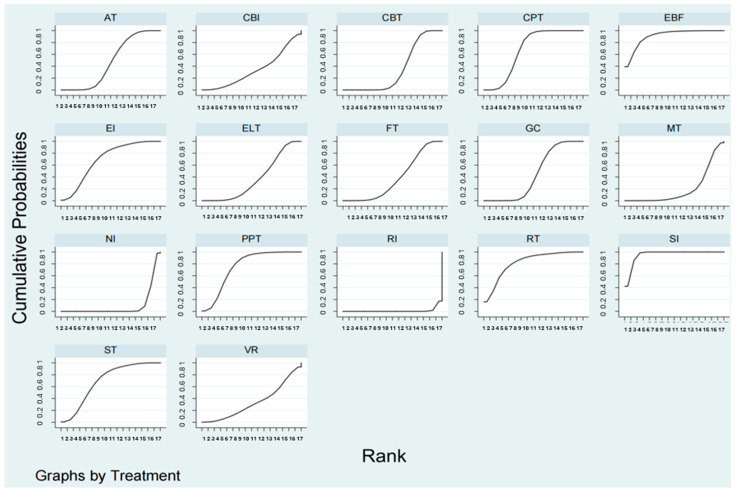
Surface under cumulative ranking curve, ranking non-pharmacological intervention effects for youths with internet addiction. AT, Electroacupuncture Therapy; CBT, Cognitive Behavior Therapy; CBI, Craving Behavior Intervention; CPT, Compound Psychotherapy; EBF, Electroencephalogram Biological Feedback; ELT, Electrotherapy; EI, Educational Intervention; FT, Family Therapy; GC, Group Counseling; MT, Mindfulness Therapy; NI, No Intervention; RI, Routine Intervention; RT, Reality Therapy; PPT, Positive Psychology Therapy; ST, Sandplay Therapy; SI, Sports Intervention; VRT, Virtual Reality Therapy.

The hierarchy of each NPI treatment was ranked using an SUCRA line (Figure 4), showing that SI had the highest probability of relieving PIU in youth when compared to the other active therapies (SUCRA = 95.43%, CrI: 0.88, 1.00). Meanwhile, EBF (SUCRA = 90.89%, CrI: 0.56, 1.00), RT (SUCRA = 81.90%, CrI: 0.31, 1.00), and PPT (SUCRA = 75.28%, CrI: 0.44, 0.94) also had a remarkable ranking. The examination results of the funnel plot for PIU indicated that there was publication bias (Figure S4).

## 4. Discussion

We conducted a Bayesian Network NMA on RCTs that examined the impact of NPIs on PIU in youth. We utilized indirect evidence in our research to evaluate the relative effectiveness of various NIP approaches. The findings indicate that SI (SUCRA = 95.43%) has the highest likelihood of being the most effective intervention for improving PIU. EBF (SUCRA = 90.89%) and RT (SUCRA = 81.90%) can be used as suboptimal treatment for young individuals to alleviate PIU. Given the limited number of NMA studies focusing on PIU among adolescents, and the absence of studies comparing the relative effectiveness of various NPIs for improving internet addiction, there is a need for more comprehensive research to provide further evidence.

Overall, a series of NPIs have demonstrated greater efficacy in improving adolescent PIU compared to conventional blank control groups and routine groups, which aligns with previous meta-analytic findings (Jiang et al., 2024). Our study included 90 papers that encompassed a diverse array of NPIs; consequently, we established four categories to classify the different types of NPIs: sports intervention, psychological intervention, electrotherapy, and compound psychotherapy.

SIs are low-cost and non-invasive approaches. NMA revealed a significant advantage of SI over other interventions in enhancing PIU (Wu et al., 2019; Wu et al., 2018). Playing online games recreationally raises the risk of PIU due to the brain’s reward system activation and neurotransmitter release, including dopamine. Repeated gaming can alter brain structure, exacerbating PIU symptoms (Kim et al., 2014). Sports can replace the reward mechanism linked to adolescent PIU by stimulating dopamine secretion (Bastioli et al., 2022) and can mitigate PIU by improving physical and mental health (Yang & Zeng, 2017).

Various psychological intervention methods, such as RT (SUCRA = 81.90%), PPT (SUCRA = 75.28%), and CBT (SUCRA = 35.07%), have been shown to effectively alleviate the symptoms of adolescent PIU. These interventions demonstrate a significant effect compared to the NI (SUCRA = 9.46%) and RI group (SUCRA = 1.20%), aligning with the findings of previous systematic reviews (Stevens et al., 2019). Excessive engagement in online gaming can lead to diminished social relationships and increased feelings of loneliness, which may manifest as symptoms of anxiety. These symptoms can exacerbate PIU, creating a detrimental cycle. Psychological therapies, such as RT and CBT, are thought to mitigate PIU levels by enhancing the quality of interpersonal relationships, alleviating feelings of loneliness, and reducing symptoms of anxiety (Adalıer & Balkan, 2012).

Another important point to emphasize is that electrical stimulation techniques, such as EBF, ELT, and AT, have been found to effectively reduce symptoms of PIU in youth. Among these techniques, EBF has demonstrated greater efficacy than other forms of electrical stimulation therapy (SUCRA = 90.89%). Electrical stimulation may suppress addictive behavior by modulating the action of neurotransmitters, such as dopamine, in the brain and specifically targeting the left dorsolateral prefrontal cortex in humans (Strafella et al., 2001; Zhang et al., 2013). Simultaneously, applying electrical stimulation to the left dorsolateral prefrontal cortex can inhibit the cerebral cortex associated with addictive behavior or sensory seeking, thereby effectively reducing an individual’s propensity for addictive behavior (Shen et al., 2016). Additionally, CPT (SUCRA = 67.85%) has also been shown to improve PIU compared to the NI and RI, aligning with previous research findings. An experimental intervention involving high-intensity exercise combined with dietary changes was conducted with 80 students in a RCT. After 8 weeks, the experimental group exhibited significant improvements in PIU symptoms compared to the NI. However, due to the presence of multiple influencing factors in mixed interventions, accurately regulating a single variable proves challenging. Consequently, the underlying mechanisms of mixed interventions in alleviating adolescent PIU symptoms require further investigation.

### Strengths and Limitations

The current study presents several advantages. It is the first to utilize NMA to validate the efficacy of various NPIs for adolescents with PIU, addressing the limitations of conventional meta-analysis by allowing for direct comparisons among multiple therapies. Additionally, this study imposed no restrictions on language or publication date. To ensure a comprehensive search, we employed multiple databases, resulting in a large and representative sample size. Furthermore, NPIs are effective in preventing medication side effects, thereby promoting the continuation of long-term interventions and facilitating the successful cessation of addictive behaviors. Consequently, this study serves as a valuable reference for future research and clinical implementation, aiding physicians and decision-makers in their clinical decision-making.

However, several limitations warrant discussion. A significant limitation is that the outcomes of our NMA may have been influenced by the considerable heterogeneity observed in several included studies. Many of these trials exhibited insufficient blinding concerning participant selection, intervention administration, or outcome measurement. Heterogeneity may be due to the population, the intervention, or the outcome measures, but the subgroup analyses conducted in this study still failed to find a cause for heterogeneity. Moreover, caution should be exercised when generalizing the results of this analysis, as a substantial number of the included studies were conducted in China. Cultural differences may affect subjects’ acceptance of non-pharmacological interventions, thereby influencing the effectiveness of the intervention outcomes. Incorporating a large number of Chinese studies may also increase the risk of selection bias. It is advisable to include only English-language articles in future research to effectively reduce the risk of selection bias in the study. Most of the research is based on Asian populations and may not represent the entire population, potentially limiting the applicability of the findings. Additionally, the low quality of certain eligible studies may jeopardize the reliability of the results; as previously noted, some studies employed imprecise blinding allocation methods and were rated as having a high risk of bias. Ultimately, the lack of quality in specific studies may compromise the dependability of the findings. As indicated earlier, many studies have utilized imprecise blinding allocation procedures and have been assessed as having a high risk of bias, and further exploration of adolescent PIU symptoms is still necessary.

## 5. Conclusions

Based on our research findings, we recommend utilizing SI as an effective therapeutic approach to prevent the onset of symptoms associated with PIU in young individuals. The underlying mechanism may be that SI can effectively replace the reward mechanisms associated with adolescent internet addiction by promoting the secretion of substances such as dopamine (Guendalina Bastioli et al., 2022; Kim et al., 2014). It is recommended to carry out interventions for adolescents with improper internet use in the forms of aerobic exercise and resistance exercise. Specifically, the intervention measures can be implemented two-to-three times a week, with each session lasting 40 to 60 min. Clinical practitioners, policymakers, and educators may consider incorporating exercise interventions into existing treatment programs to help address the issue of internet addiction among adolescents. However, further investigation is necessary to substantiate these findings, as the studies included in the analysis were of inadequate quality. Conducting high-quality RCTs is crucial for developing effective management and prevention interventions for PIU among adolescents.

## Data Availability

Some or all data generated or analyzed during this study are included in this published article or in the data repositories listed in References.

## Supplementary Materials

The following supporting information can be downloaded at: https://www.mdpi.com/article/10.3390/bs15010098/s1. Supplement Appendix S1. Search strategy; Supplement Appendix S2. Raw data; Supplement Appendix S3. List of excluded studies; Supplement Appendix S4. The list of included studies; Supplement Appendix S5. Author contribution; Supplement Appendix S6. PRISMA checklist; Supplement Figure S1. Risk of bias summary; Supplement Figure S2. Risk of bias graph; Supplement Figure S3. Funnel plot; Supplement Figure S4. Funnel plot; Supplement Table S1. Inclusion and Exclusion criteria; Supplement Table S2. Description of NPIs; Supplement Table S3. Demographic characteristics.
