# Supplementary material for: Optimal Non-Pharmacological Interventions for Reducing Problematic Internet Use in Youth: A Systematic Review and Bayesian Network Meta-Analysis
Source: Behav Sci (Basel). 2025 Jan 20;15(1):98. doi: 10.3390/bs15010098 (PMC11762159; doi:10.3390/bs15010098)
Supplement: Supplementary file 1 [file behavsci-15-00098-s001.zip › Supplement Appendix S1 Search strategy.pdf]

Web of science Search Strategy

- WOS: 1900 to 2023
- CSCD: 1989 to 2023
- KJD: 1980 to 2023
- MEDLINE: 1950 to 2023
- PPRN: 1991 to 2023
- PQDT: 1637 to 2023
- SCIELO: 2002 to 2023

Search:

1: (((TS=(Internet Addiction Disorder)) OR TS=(Addiction Disorder, Internet)) OR TS=(Addiction Disorders, Internet)) OR TS=(Disorder, Internet Addiction)) OR TS=(Disorders, Internet Addiction)) OR TS=(Internet Addiction Disorders)) OR TS=(Internet Addiction)) OR TS=(Addiction, Internet)) OR TS=(Addictions, Internet)) OR TS=(Internet Addictions)) OR TS=(Social Media Addiction)) OR TS=(Addiction, Social Media)) OR TS=(Addictions, Social Media)) OR TS=(Media Addiction, Social)) OR TS=(Media Addictions, Social)) OR TS=(Social Media Addictions)) OR TS=(Smartphone Addiction)) OR TS=(Addiction, Smartphone)) OR TS=(Addictions, Smartphone)) OR TS=(Smartphone Addictions)) OR TS=(Internet Gaming Disorder)) OR TS=(Disorder, Internet Gaming)) OR TS=(Disorders, Internet Gaming)) OR TS=(Gaming Disorder, Internet)) OR TS=(Gaming Disorders, Internet)) OR TS=(Internet Gaming Disorders)) OR TS=(problematic Internet use)) OR TS=(internet use disorder)) OR TS=(internet use)) OR TS=(Internet Uses)) OR TS=(Use, Internet)) OR TS=(Web Usage)) OR TS=(Usage, Web)) OR TS=(Web Use)) OR TS=(Use, Web)) OR TS=(Internet Usage)) OR TS=(Usage, Internet)) OR TS=(Internet-addicted)) OR TS=(Internet depend)) OR TS=(Internet overuse)) OR TS=(Compulsive internet)) OR TS=(Pathological internet)) OR TS=(Excessive internet)) OR TS=(Addictive internet)) OR TS=(Video game addiction)) OR TS=(Online game addiction)) OR TS=(Cybersex addiction)) OR TS=(Internet sex addiction)) OR TS=(Social network addiction)) OR TS=(Facebook addiction) and Preprint Citation Index (Troubleshooting - Database) Run Date: Thu Dec 21 2023 10:02:25 GMT+0800 (CST) Retrieved: 809339

2: TS= clinical trial* OR TS=research design OR TS=comparative stud* OR TS=evaluation stud* OR TS=controlled trial* OR TS=follow-up stud* OR TS=prospective stud* OR TS=random* OR TS=placebo* OR TS=(single blind*) OR TS=(double blind*) and Preprint Citation Index (Exclusions - Database) Run Date: Thu Dec 21 2023 10:02:49 GMT+0800 (CST) Retrieved: 14296321

3: ((((((((((((((((((((((((((((TS=(Behavioral Therapies, Cognitive)) OR TS=(Cognitive

Behavioral Therapy)) OR TS=(Behavioral Therapy, Cognitive)) OR TS=(Cognitive Behavioral Therapies)) OR TS=(Therapies, Cognitive Behavioral)) OR TS=(Therapy, Cognitive Behavioral)) OR TS=(Psychotherapy, Cognitive)) OR TS=(Therapy, Cognitive)) OR TS=(Cognitive Therapies)) OR TS=(Therapies, Cognitive)) OR TS=(Cognitive Therapy)) OR TS=(Cognitive Behaviour Therapy)) OR TS=(Behaviour Therapies, Cognitive)) OR TS=(Behaviour Therapy, Cognitive)) OR TS=(Cognitive Behaviour Therapies)) OR TS=(Therapies, Cognitive Behaviour)) OR TS=(Therapy, Cognitive Behaviour)) OR TS=(Cognitive Psychotherapy)) OR TS=(Cognitive Psychotherapies)) OR TS=(Psychotherapies, Cognitive)) OR TS=(Cognition Therapy)) OR TS=(Cognition Therapies)) OR TS=(Therapies, Cognition)) OR TS=(Therapy, Cognitive Behavior)) OR TS=(Behavior Therapies, Cognitive)) OR TS=(Cognitive Behavior Therapies)) OR TS=(Therapies, Cognitive Behavior)) OR TS=(Therapy, Cognition)) OR TS=(Behavior Therapy, Cognitive)) OR TS=(Cognitive Behavior Therapy) and Preprint Citation Index (Exclusions - Database) Run Date: Thu Aug 21 2023 10:03:09 GMT+0800 (CST) Retrieved: 375182

4: ((TS=(Exercises)) OR TS=(exercise)) OR TS=(Physical Activity)) OR TS=(Activities, Physical)) OR TS=(Activity, Physical)) OR TS=(Physical Activities)) OR TS=(Exercise, Physical)) OR TS=(Exercises, Physical)) OR TS=(Physical Exercise)) OR TS=(Physical Exercises)) OR TS=(Acute Exercise)) OR TS=(Acute Exercises)) OR TS=(Exercise, Acute)) OR TS=(Exercises, Acute)) OR TS=(Exercise, Isometric)) OR TS=(Exercises, Isometric)) OR TS=(Isometric Exercises)) OR TS=(Isometric Exercise)) OR TS=(Exercise, Aerobic)) OR TS=(Aerobic Exercise)) OR TS=(Aerobic Exercises)) OR TS=(Exercises, Aerobic)) OR TS=(Exercise Training)) OR TS=(Exercise Trainings)) OR TS=(Training, Exercise)) OR TS=(Trainings, Exercise)) OR TS=(football)) OR TS=(Tai Chi)) OR TS=(basketball)) OR TS=(High-Intensity Interval Training)) OR TS=(High Intensity Interval Training)) OR TS=(High-Intensity Interval Trainings)) OR TS=(Interval Training, High-Intensity)) OR TS=(Interval Trainings, High-Intensity)) OR TS=(Training, High-Intensity Interval)) OR TS=(Trainings, High-Intensity Interval)) OR TS=(High-Intensity Intermittent Exercise)) OR TS=(Exercise, High-Intensity Intermittent)) OR TS=(High-Intensity Intermittent Exercises)) OR TS=(Sprint Interval Training)) OR TS=(Sprint Interval Trainings) and Preprint Citation Index (Exclusions - Database) Run Date: Thu Dec 21 2023 10:03:39 GMT+0800 (CST) Retrieved: 1851049

5: ((((((((((((((((((((((TS=(tDCS)) OR TS=(Cathodal Stimulation Transcranial Direct Current Stimulation)) OR TS=(Cathodal Stimulation tDCS)) OR TS=(Cathodal Stimulation tDCSs)) OR TS=(Stimulation tDCS, Cathodal)) OR TS=(Stimulation tDCSs, Cathodal)) OR TS=(tDCS, Cathodal Stimulation)) OR TS=(tDCSs, Cathodal Stimulation)) OR TS=(Transcranial Random Noise Stimulation)) OR TS=(Transcranial Alternating Current Stimulation)) OR TS=(Transcranial Electrical Stimulation)) OR TS=(Electrical Stimulation, Transcranial)) OR TS=(Electrical Stimulations, Transcranial)) OR TS=(Stimulation, Transcranial Electrical)) OR TS=(Stimulations, Transcranial Electrical)) OR TS=(Transcranial Electrical Stimulations)) OR

TS=(Anodal Stimulation Transcranial Direct Current Stimulation)) OR TS=(Anodal Stimulation tDCS)) OR TS=(Anodal Stimulation tDCSs)) OR TS=(Stimulation tDCS, Anodal)) OR TS=(Stimulation tDCSs, Anodal)) OR TS=(tDCS, Anodal Stimulation)) OR TS=(tDCSs, Anodal Stimulation)) OR TS=(Repetitive Transcranial Electrical Stimulation) and Preprint Citation Index (Exclusions - Database) Run Date: Thu Dec 21 2023 10:03:54 GMT+0800 (CST) Retrieved: 16770

6: (((((((TS=(Virtual Reality Exposure Therapy)) OR TS=(Virtual Reality Immersion Therapy)) OR TS=(Virtual Reality Therapy)) OR TS=(Reality Therapies, Virtual)) OR TS=(Reality Therapy, Virtual)) OR TS=(Therapies, Virtual Reality)) OR TS=(Therapy, Virtual Reality)) OR TS=(Virtual Reality Therapies) and Preprint Citation Index (Exclusions - Database) Run Date: Thu Dec 21 2023 10:04:01 GMT+0800 (CST) Retrieved: 10920

7: ((((TS=(family intervention)) OR TS=(counseling)) OR TS=(Mindfulness)) OR TS=(acupuncture)) OR TS=(nursing) and Preprint Citation Index (Exclusions - Database) Run Date: Thu Dec 21 2023 10:04:09 GMT+0800 (CST) Retrieved: 1560783

8: (((((((((TS=(sandplay therapy)) OR TS=(Play Therapies)) OR TS=(Therapies, Play)) OR TS=(Therapy, Play)) OR TS=(Sandplay Therapy)) OR TS=(Sandplay Therapies)) OR TS=(Therapies, Sandplay)) OR TS=(Therapy, Sandplay)) OR TS=(Sandplay)) OR TS=(Sandplays) and Preprint Citation Index (Exclusions - Database) Run Date: Thu Dec 21 2023 10:04:18 GMT+0800 (CST) Retrieved: 408667

9: (TS=(Psychology, Positive)) OR TS=(Positive Psychology) and Preprint Citation Index (Exclusions - Database) Run Date: Thu Dec 21 2023 10:04:31 GMT+0800 (CST) Retrieved: 208886

10: TS=(group counselling) and Preprint Citation Index (Exclusions - Database) Run Date: Thu Dec 21 2023 10:08:19 GMT+0800 (CST) Retrieved: 65581

11: TS=(routine intervention) and Preprint Citation Index (Exclusions - Database) Run Date: Thu Dec 21 2023 10:08:42 GMT+0800 (CST) Retrieved: 56148

12: TS=(education intervention) and Preprint Citation Index (Exclusions - Database) Run Date: Thu Dec 21 2023 10:09:05 GMT+0800 (CST) Retrieved: 254593

13: ((((((((((((((TS=(Telemedicine)) OR TS=(Tele-Referral)) OR TS=(Tele Referral)) OR TS=(Tele-Referrals)) OR TS=(Virtual Medicine)) OR TS=(Medicine, Virtual)) OR TS=(Tele-Intensive Care)) OR TS=(Tele Intensive Care)) OR TS=(Tele-ICU)) OR TS=(Tele ICU)) OR TS=(Mobile Health)) OR TS=(Health, Mobile)) OR TS=(mHealth)) OR TS=(Telehealth)) OR TS=(eHealth) and Preprint Citation Index (Exclusions - Database) Run Date: Thu Dec 21 2023 10:12:20 GMT+0800 (CST) Result: 192519

14: ((TS=(Multi-level Counseling Program)) OR TS=(combined intervention)) OR TS=(mixed intervention) and Preprint Citation Index (Exclusions - Database) Run Date: Thu Dec 21 2023 10:15:34 GMT+0800 (CST) Result: 202437

15: #14 OR #13 OR #12 OR #11 OR #10 OR #9 OR #8 OR #7 OR #6 OR #5 OR #4 OR #3 and Preprint Citation Index (Exclusions - Database) Run Date: Thu Dec 21 2023 10:16:11 GMT+0800 (CST) Retrieved: 4636359

16: #15 AND #1 AND #2 and Preprint Citation Index (Exclusions - Database) Run Date: Thu Dec 21 2023 10:16:24 GMT+0800 (CST) Retrieved: 40679

Cochrane library
Search Name:
Date Run: 22/12/2023 06:01:13
Comment:

ID Search Hits
#1 MeSH descriptor: [Internet Addiction Disorder] explode all trees 29
#2 (Addiction Disorder, Internet):ti,ab,kw OR (Addiction Disorders, Internet):ti,ab,kw OR (Disorder, Internet Addiction):ti,ab,kw OR (Disorders, Internet Addiction):ti,ab,kw OR (Internet Addiction Disorders):ti,ab,kw (Word variations have been searched) 237
#3 (Internet Addiction):ti,ab,kw OR (Addiction, Internet):ti,ab,kw OR (Addictions, Internet):ti,ab,kw OR (Internet Addictions):ti,ab,kw OR (Social Media Addiction):ti,ab,kw (Word variations have been searched) 457
#4 (Smartphone Addiction):ti,ab,kw OR (Addiction, Smartphone):ti,ab,kw OR (Addictions, Smartphone):ti,ab,kw OR (Smartphone Addictions):ti,ab,kw OR (Internet Gaming Disorder):ti,ab,kw (Word variations have been searched) 234
#5 (Addiction, Social Media):ti,ab,kw OR (Addictions, Social Media):ti,ab,kw OR (Media Addiction, Social):ti,ab,kw OR (Media Addictions, Social):ti,ab,kw OR (Social Media Addictions):ti,ab,kw (Word variations have been searched) 93
#6 (Disorder, Internet Gaming):ti,ab,kw OR (Disorders, Internet Gaming):ti,ab,kw OR (Gaming Disorder, Internet):ti,ab,kw OR (Gaming Disorders, Internet):ti,ab,kw OR (Internet Gaming Disorders):ti,ab,kw (Word variations have been searched) 94
#7 (problematic Internet use):ti,ab,kw OR (internet use disorder):ti,ab,kw OR (internet use):ti,ab,kw OR (Internet Uses):ti,ab,kw OR (Use, Internet):ti,ab,kw (Word variations have been searched) 5888
#8 (Web Usage):ti,ab,kw OR (Usage, Web):ti,ab,kw OR (Web Use):ti,ab,kw OR (Use, Web):ti,ab,kw OR (Internet Usage):ti,ab,kw (Word variations have been searched) 7883
#9 (Usage, Internet):ti,ab,kw OR (Internet-addicted):ti,ab,kw OR (Internet depend):ti,ab,kw OR (Internet overuse):ti,ab,kw OR (Compulsive internet):ti,ab,kw (Word variations have been searched) 832

#10 (Pathological internet):ti,ab,kw OR (Excessive internet):ti,ab,kw OR (Addictive internet):ti,ab,kw OR (Video game addiction):ti,ab,kw OR (Online game addiction):ti,ab,kw (Word variations have been searched) 398
#11 (Cybersex addiction):ti,ab,kw OR (Internet sex addiction):ti,ab,kw OR (Social network addiction):ti,ab,kw OR (Facebook addiction):ti,ab,kw (Word variations have been searched) 112
#12 #1 or #2 or #3 or #4 or #5 or #6 or #7 or #8 or #9 or #10 or #11 12161
#13 MeSH descriptor: [Cognitive Behavioral Therapy] explode all trees 12999
#14 (Cognitive Behaviour Therapy):ti,ab,kw OR (Behaviour Therapies, Cognitive):ti,ab,kw OR (Behaviour Therapy, Cognitive):ti,ab,kw OR (Cognitive Behaviour Therapies):ti,ab,kw AND (Therapies, Cognitive Behaviour):ti,ab,kw (Word variations have been searched) 11953
#15 (Therapy, Cognitive Behaviour):ti,ab,kw OR (Cognitive Psychotherapy):ti,ab,kw OR (Cognitive Psychotherapies):ti,ab,kw OR (Psychotherapies, Cognitive):ti,ab,kw AND (Cognition Therapy):ti,ab,kw (Word variations have been searched) 15677
#16 (Cognition Therapies):ti,ab,kw OR (Therapies, Cognition):ti,ab,kw OR (Therapy, Cognitive Behavior):ti,ab,kw OR (Behavior Therapies, Cognitive):ti,ab,kw AND (Cognitive Behavior Therapies):ti,ab,kw (Word variations have been searched) 12479
#17 (Behavior Therapies, Cognitive):ti,ab,kw OR (Cognitive Behavior Therapies):ti,ab,kw OR (Therapies, Cognitive Behavior):ti,ab,kw OR (Therapy, Cognition):ti,ab,kw OR (Behavior Therapy, Cognitive):ti,ab,kw (Word variations have been searched) 25329
#18 #13 or #14 or #15 or #16 or #17 35609
#19 (Virtual Reality Exposure Therapy):ti,ab,kw OR (Virtual Reality Immersion Therapy):ti,ab,kw OR (Virtual Reality Therapy):ti,ab,kw OR (Reality Therapies, Virtual):ti,ab,kw OR (Reality Therapy, Virtual):ti,ab,kw (Word variations have been searched) 2189
#20 (Therapies, Virtual Reality):ti,ab,kw OR (Therapy, Virtual Reality):ti,ab,kw OR (Virtual Reality Therapies):ti,ab,kw OR (Reality Therapies, Virtual):ti,ab,kw OR (Reality Therapy, Virtual):ti,ab,kw (Word variations have been searched) 2189
#21 #19 or #20 2189
#22 MeSH descriptor: [Exercise] explode all trees 38402
#23 (Exercise, Physical):ti,ab,kw OR (Exercises, Physical):ti,ab,kw OR (Physical Exercise):ti,ab,kw OR (Physical Exercises):ti,ab,kw OR (Acute Exercise):ti,ab,kw (Word variations have been searched) 60772
#24 (Acute Exercises):ti,ab,kw OR (Exercise, Acute):ti,ab,kw OR (Exercises, Acute):ti,ab,kw OR (Exercise, Isometric):ti,ab,kw OR (Exercises, Isometric):ti,ab,kw (Word variations have been searched) 16094
#25 (football):ti,ab,kw OR (Tai Chi):ti,ab,kw OR (basketball):ti,ab,kw OR (High-Intensity Interval Training):ti,ab,kw OR (High Intensity Interval Training):ti,ab,kw (Word variations have been searched) 7555
#26 (High-Intensity Interval Trainings):ti,ab,kw OR (Interval Training, High-Intensity):ti,ab,kw OR (Interval Trainings, High-Intensity):ti,ab,kw OR (Training,

High-Intensity Interval):ti,ab,kw OR (Trainings, High-Intensity Interval):ti,ab,kw (Word variations have been searched) 3543
#27 (High-Intensity Intermittent Exercise):ti,ab,kw OR (Exercise, High-Intensity Intermittent):ti,ab,kw OR (Exercises, High-Intensity Intermittent):ti,ab,kw OR (High-Intensity Intermittent Exercises):ti,ab,kw OR (Sprint Interval Training):ti,ab,kw (Word variations have been searched) 916
#28 #22 or #23 or #24 or #25 or #26 or #27 85548
#29 (family intervention):ti,ab,kw OR (counseling):ti,ab,kw OR (Mindfulness):ti,ab,kw OR (acupuncture):ti,ab,kw OR (nursing):ti,ab,kw (Word variations have been searched) 101521
#30 MeSH descriptor: [Play Therapy] explode all trees 97
#31 (Therapies, Sandplay):ti,ab,kw OR (Therapy, Sandplay):ti,ab,kw OR (Sandplay):ti,ab,kw OR (Sandplays):ti,ab,kw (Word variations have been searched) 22
#32 MeSH descriptor: [Transcranial Direct Current Stimulation] explode all trees 1755
#33 (tDCS):ti,ab,kw OR (Cathodal Stimulation Transcranial Direct Current Stimulation):ti,ab,kw OR (Cathodal Stimulation tDCS):ti,ab,kw OR (Cathodal Stimulation tDCSs):ti,ab,kw OR (Stimulation tDCS, Cathodal):ti,ab,kw (Word variations have been searched) 5213
#34 (Stimulation tDCSs, Anodal):ti,ab,kw OR (tDCS, Anodal Stimulation):ti,ab,kw OR (tDCSs, Anodal Stimulation):ti,ab,kw OR (Repetitive Transcranial Electrical Stimulation):ti,ab,kw (Word variations have been searched) 2371
#35 #30 or #31 or #32 or #33 or #34 5805
#36 MeSH descriptor: [Psychology, Positive] explode all trees 48
#37 (Positive Psychology):ti,ab,kw (Word variations have been searched) 12018
#38 #36 or #37 12018
#39 #18 or #21 or #28 or #29 or #35 or #38 222050
#40 #12 and #39 3713

PubMed Search Strategy

#1((((((((((((Psychology, Positive[MeSH Terms]) OR (Positive Psychology[Title/Abstract])) OR (((((((((((((((((((((((tDCS[MeSH Terms]) OR (Cathodal Stimulation Transcranial Direct Current Stimulation[Title/Abstract])) OR (Cathodal Stimulation tDCS[Title/Abstract])) OR (Cathodal Stimulation tDCSs[Title/Abstract])) OR (Stimulation tDCS, Cathodal[Title/Abstract])) OR (Stimulation tDCSs, Cathodal[Title/Abstract])) OR (tDCS, Cathodal Stimulation[Title/Abstract])) OR (tDCSs, Cathodal Stimulation[Title/Abstract])) OR (Transcranial Random Noise Stimulation[Title/Abstract])) OR (Transcranial Alternating Current Stimulation[Title/Abstract])) OR (Transcranial Electrical Stimulation[Title/Abstract])) OR (Electrical Stimulation, Transcranial[Title/Abstract])) OR (Electrical Stimulations, Transcranial[Title/Abstract])) OR (Stimulation, Transcranial Electrical[Title/Abstract])) OR (Stimulations, Transcranial

Electrical[Title/Abstract])) OR (Transcranial Electrical Stimulations[Title/Abstract])) OR (Anodal Stimulation Transcranial Direct Current Stimulation[Title/Abstract])) OR (Anodal Stimulation tDCS[Title/Abstract])) OR (Anodal Stimulation tDCSs[Title/Abstract])) OR (Stimulation tDCS, Anodal[Title/Abstract])) OR (Stimulation tDCSs, Anodal[Title/Abstract])) OR (tDCS, Anodal Stimulation[Title/Abstract])) OR (tDCSs, Anodal Stimulation[Title/Abstract])) OR (Repetitive Transcranial Electrical Stimulation[Title/Abstract]))) OR ((exercise[MeSH Terms]) OR (Exercises[Title/Abstract])) OR (Physical Activity[Title/Abstract])) OR (Activities, Physical[Title/Abstract])) OR (Activity, Physical[Title/Abstract])) OR (Physical Activities[Title/Abstract])) OR (Exercise, Physical[Title/Abstract])) OR (Exercises, Physical[Title/Abstract])) OR (Physical Exercise[Title/Abstract])) OR (Physical Exercises[Title/Abstract])) OR (Acute Exercise[Title/Abstract])) OR (Acute Exercises[Title/Abstract])) OR (Exercise, Acute[Title/Abstract])) OR (Exercises, Acute[Title/Abstract])) OR (Exercise, Isometric[Title/Abstract])) OR (Exercises, Isometric[Title/Abstract])) OR (Isometric Exercises[Title/Abstract])) OR (Isometric Exercise[Title/Abstract])) OR (Exercise, Aerobic[Title/Abstract])) OR (Aerobic Exercise[Title/Abstract])) OR (Aerobic Exercises[Title/Abstract])) OR (Exercises, Aerobic[Title/Abstract])) OR (Exercise Training[Title/Abstract])) OR (Exercise Trainings[Title/Abstract])) OR (Training, Exercise[Title/Abstract])) OR (Trainings, Exercise[Title/Abstract])) OR (football[Title/Abstract])) OR (Tai Chi[Title/Abstract])) OR (basketball[Title/Abstract])) OR (High-Intensity Interval Training[Title/Abstract])) OR (High Intensity Interval Training[Title/Abstract])) OR (High-Intensity Interval Trainings[Title/Abstract])) OR (Interval Training, High-Intensity[Title/Abstract])) OR (Interval Trainings, High-Intensity[Title/Abstract])) OR (Training, High-Intensity Interval[Title/Abstract])) OR (Trainings, High-Intensity Interval[Title/Abstract])) OR (High-Intensity Intermittent Exercise[Title/Abstract])) OR (Exercise, High-Intensity Intermittent[Title/Abstract])) OR (Exercises, High-Intensity Intermittent[Title/Abstract])) OR (High-Intensity Intermittent Exercises[Title/Abstract])) OR (Sprint Interval Training[Title/Abstract])) OR (Sprint Interval Trainings[Title/Abstract]))) OR ((((((((((sandplay therapy[MeSH Terms]) OR (Play Therapies[Title/Abstract])) OR (Therapies, Play[Title/Abstract])) OR (Therapy, Play[Title/Abstract])) OR (Sandplay Therapy[Title/Abstract])) OR (Sandplay Therapies[Title/Abstract])) OR (Therapies, Sandplay[Title/Abstract])) OR (Therapy, Sandplay[Title/Abstract])) OR (Sandplay[Title/Abstract])) OR (Sandplays[Title/Abstract]))) OR (((((family intervention[Title/Abstract]) OR (counseling[Title/Abstract])) OR (Mindfulness[Title/Abstract])) OR (acupuncture[Title/Abstract])) OR (nursing[Title/Abstract]))) OR ((((((((Virtual Reality Exposure Therapy[MeSH Terms]) OR (Virtual Reality Immersion Therapy[Title/Abstract])) OR (Virtual Reality Therapy[Title/Abstract])) OR (Reality Therapies, Virtual[Title/Abstract])) OR (Reality Therapy, Virtual[Title/Abstract])) OR (Therapies, Virtual Reality[Title/Abstract])) OR (Therapy, Virtual Reality[Title/Abstract])) OR (Virtual Reality Therapies[Title/Abstract]))) OR ((((((((((((((((((((((((((((((Cognitive Behavioral Therapy[Title/Abstract]) OR

(Behavioral Therapies, Cognitive[Title/Abstract])) OR (Behavioral Therapy, Cognitive[Title/Abstract])) OR (Cognitive Behavioral Therapies[Title/Abstract])) OR (Therapies, Cognitive Behavioral[Title/Abstract])) OR (Therapy, Cognitive Behavioral[Title/Abstract])) OR (Psychotherapy, Cognitive[Title/Abstract])) OR (Therapy, Cognitive[Title/Abstract])) OR (Cognitive Therapies[Title/Abstract])) OR (Therapies, Cognitive[Title/Abstract])) OR (Cognitive Therapy[Title/Abstract])) OR (Cognitive Behaviour Therapy[Title/Abstract])) OR (Behaviour Therapies, Cognitive[Title/Abstract])) OR (Behaviour Therapy, Cognitive[Title/Abstract])) OR (Cognitive Behaviour Therapies[Title/Abstract])) OR (Therapies, Cognitive Behaviour[Title/Abstract])) OR (Therapy, Cognitive Behaviour[Title/Abstract])) OR (Cognitive Psychotherapy[Title/Abstract])) OR (Cognitive Psychotherapies[Title/Abstract])) OR (Psychotherapies, Cognitive[Title/Abstract])) OR (Cognition Therapy[Title/Abstract])) OR (Cognition Therapies[Title/Abstract])) OR (Therapies, Cognition[Title/Abstract])) OR (Therapy, Cognitive Behavior[Title/Abstract])) OR (Behavior Therapies, Cognitive[Title/Abstract])) OR (Cognitive Behavior Therapies[Title/Abstract])) OR (Therapies, Cognitive Behavior[Title/Abstract])) OR (Therapy, Cognition[Title/Abstract])) OR (Behavior Therapy, Cognitive[Title/Abstract])) OR (Cognitive Behavior Therapy[Title/Abstract]))) OR (group counselling[Title/Abstract])) OR (routine intervention[Title/Abstract])) OR (education intervention[Title/Abstract])) OR (((((((((((((((TS=(Telemedicine[Title/Abstract])) OR TS=(Tele-Referral[Title/Abstract])) OR TS=(Tele Referral[Title/Abstract])) OR TS=(Tele-Referrals[Title/Abstract])) OR TS=(Virtual Medicine[Title/Abstract])) OR TS=(Medicine, Virtual[Title/Abstract])) OR TS=(Tele-Intensive Care[Title/Abstract])) OR TS=(Tele Intensive Care[Title/Abstract])) OR TS=(Tele-ICU[Title/Abstract])) OR TS=(Tele ICU[Title/Abstract])) OR TS=(Mobile Health[Title/Abstract])) OR TS=(Health, Mobile[Title/Abstract])) OR TS=(mHealth[Title/Abstract])) OR TS=(Telehealth[Title/Abstract])) OR TS=(eHealth[Title/Abstract]))) OR (((TS=(Multi-level Counseling Program[Title/Abstract])) OR TS=(combined intervention[Title/Abstract])) OR TS=(mixed intervention[Title/Abstract]))

#2(randomized controlled trial [pt] OR controlled clinical trial [pt] OR randomized [tiab] OR placebo [tiab] OR drug therapy [sh] OR randomly [tiab] OR trial [tiab] OR groups [tiab]) NOT (animals [mh] NOT humans [mh])

#3(("Internet Addiction Disorder"[Mesh]) OR (((Addiction Disorder, Internet[Title/Abstract]) OR (Addiction Disorders, Internet[Title/Abstract])) OR (Disorder, Internet Addiction[Title/Abstract])) OR (Disorders, Internet Addiction[Title/Abstract])) OR (Internet Addiction Disorders[Title/Abstract])) OR (Internet Addiction[Title/Abstract])) OR (Addiction, Internet[Title/Abstract])) OR (Addictions, Internet[Title/Abstract])) OR (Internet Addictions[Title/Abstract])) OR (Social Media Addiction[Title/Abstract])) OR (Addiction, Social Media[Title/Abstract])) OR (Addictions, Social Media[Title/Abstract])) OR (Media Addiction, Social[Title/Abstract])) OR (Media Addictions, Social[Title/Abstract])) OR (Social Media Addictions[Title/Abstract])) OR (Smartphone Addiction[Title/Abstract])) OR (Addiction, Smartphone[Title/Abstract]))

OR (Addictions, Smartphone[Title/Abstract])) OR (Smartphone Addictions[Title/Abstract])) OR (Internet Gaming Disorder[Title/Abstract])) OR (Disorder, Internet Gaming[Title/Abstract])) OR (Disorders, Internet Gaming[Title/Abstract])) OR (Gaming Disorder, Internet[Title/Abstract])) OR (Gaming Disorders, Internet[Title/Abstract])) OR (Internet Gaming Disorders[Title/Abstract])) OR (problematic Internet use[Title/Abstract])) OR (internet use disorder[Title/Abstract])) OR (internet use[Title/Abstract])) OR (Internet Uses[Title/Abstract])) OR (Use, Internet[Title/Abstract])) OR (Web Usage[Title/Abstract])) OR (Usage, Web[Title/Abstract])) OR (Web Use[Title/Abstract])) OR (Use, Web[Title/Abstract])) OR (Internet Usage[Title/Abstract])) OR (Usage, Internet[Title/Abstract])) OR (Internet-addicted[Title/Abstract])) OR (Internet depend[Title/Abstract])) OR (Internet overuse[Title/Abstract])) OR (Compulsive internet[Title/Abstract])) OR (Pathological internet[Title/Abstract])) OR (Excessive internet[Title/Abstract])) OR (Addictive internet[Title/Abstract])) OR (Video game addiction[Title/Abstract])) OR (Online game addiction[Title/Abstract])) OR (Cybersex addiction[Title/Abstract])) OR (Internet sex addiction[Title/Abstract])) OR (Social network addiction[Title/Abstract])) OR (Facebook addiction[Title/Abstract])))
#4((((((((((((((Psychology, Positive[MeSH Terms]) OR (Positive Psychology[Title/Abstract])) OR ((((((((((((((((((((((((tDCS[MeSH Terms]) OR (Cathodal Stimulation Transcranial Direct Current Stimulation[Title/Abstract])) OR (Cathodal Stimulation tDCS[Title/Abstract])) OR (Cathodal Stimulation tDCSs[Title/Abstract])) OR (Stimulation tDCS, Cathodal[Title/Abstract])) OR (Stimulation tDCSs, Cathodal[Title/Abstract])) OR (tDCS, Cathodal Stimulation[Title/Abstract])) OR (tDCSs, Cathodal Stimulation[Title/Abstract])) OR (Transcranial Random Noise Stimulation[Title/Abstract])) OR (Transcranial Alternating Current Stimulation[Title/Abstract])) OR (Transcranial Electrical Stimulation[Title/Abstract])) OR (Electrical Stimulation, Transcranial[Title/Abstract])) OR (Electrical Stimulations, Transcranial[Title/Abstract])) OR (Stimulation, Transcranial Electrical[Title/Abstract])) OR (Stimulations, Transcranial Electrical[Title/Abstract])) OR (Transcranial Electrical Stimulations[Title/Abstract])) OR (Anodal Stimulation Transcranial Direct Current Stimulation[Title/Abstract])) OR (Anodal Stimulation tDCS[Title/Abstract])) OR (Anodal Stimulation tDCSs[Title/Abstract])) OR (Stimulation tDCS, Anodal[Title/Abstract])) OR (Stimulation tDCSs, Anodal[Title/Abstract])) OR (tDCS, Anodal Stimulation[Title/Abstract])) OR (tDCSs, Anodal Stimulation[Title/Abstract])) OR (Repetitive Transcranial Electrical Stimulation[Title/Abstract]))) OR ((exercise[MeSH Terms]) OR (Exercises[Title/Abstract])) OR (Physical Activity[Title/Abstract])) OR (Activities, Physical[Title/Abstract])) OR (Activity, Physical[Title/Abstract])) OR (Physical Activities[Title/Abstract])) OR (Exercise, Physical[Title/Abstract])) OR (Exercises, Physical[Title/Abstract])) OR (Physical Exercise[Title/Abstract])) OR (Physical Exercises[Title/Abstract])) OR (Acute Exercise[Title/Abstract])) OR (Acute Exercises[Title/Abstract])) OR (Exercise, Acute[Title/Abstract])) OR (Exercises,

Acute[Title/Abstract])) OR (Exercise, Isometric[Title/Abstract])) OR (Exercises, Isometric[Title/Abstract])) OR (Isometric Exercises[Title/Abstract])) OR (Isometric Exercise[Title/Abstract])) OR (Exercise, Aerobic[Title/Abstract])) OR (Aerobic Exercise[Title/Abstract])) OR (Aerobic Exercises[Title/Abstract])) OR (Exercises, Aerobic[Title/Abstract])) OR (Exercise Training[Title/Abstract])) OR (Exercise Trainings[Title/Abstract])) OR (Training, Exercise[Title/Abstract])) OR (Trainings, Exercise[Title/Abstract])) OR (football[Title/Abstract])) OR (Tai Chi[Title/Abstract])) OR (basketball[Title/Abstract])) OR (High-Intensity Interval Training[Title/Abstract])) OR (High Intensity Interval Training[Title/Abstract])) OR (High-Intensity Interval Trainings[Title/Abstract])) OR (Interval Training, High-Intensity[Title/Abstract])) OR (Interval Trainings, High-Intensity[Title/Abstract])) OR (Training, High-Intensity Interval[Title/Abstract])) OR (Trainings, High-Intensity Interval[Title/Abstract])) OR (High-Intensity Intermittent Exercise[Title/Abstract])) OR (Exercise, High-Intensity Intermittent[Title/Abstract])) OR (Exercises, High-Intensity Intermittent[Title/Abstract])) OR (High-Intensity Intermittent Exercises[Title/Abstract])) OR (Sprint Interval Training[Title/Abstract])) OR (Sprint Interval Trainings[Title/Abstract]))) OR (((((((((sandplay therapy[MeSH Terms]) OR (Play Therapies[Title/Abstract])) OR (Therapies, Play[Title/Abstract])) OR (Therapy, Play[Title/Abstract])) OR (Sandplay Therapy[Title/Abstract])) OR (Sandplay Therapies[Title/Abstract])) OR (Therapies, Sandplay[Title/Abstract])) OR (Therapy, Sandplay[Title/Abstract])) OR (Sandplay[Title/Abstract])) OR (Sandplays[Title/Abstract]))) OR (((((family intervention[Title/Abstract]) OR (counseling[Title/Abstract])) OR (Mindfulness[Title/Abstract])) OR (acupuncture[Title/Abstract])) OR (nursing[Title/Abstract]))) OR ((((((((Virtual Reality Exposure Therapy[MeSH Terms]) OR (Virtual Reality Immersion Therapy[Title/Abstract])) OR (Virtual Reality Therapy[Title/Abstract])) OR (Reality Therapies, Virtual[Title/Abstract])) OR (Reality Therapy, Virtual[Title/Abstract])) OR (Therapies, Virtual Reality[Title/Abstract])) OR (Therapy, Virtual Reality[Title/Abstract])) OR (Virtual Reality Therapies[Title/Abstract]))) OR ((((((((((((((((((((((((((((Cognitive Behavioral Therapy[Title/Abstract]) OR (Behavioral Therapies, Cognitive[Title/Abstract])) OR (Behavioral Therapy, Cognitive[Title/Abstract])) OR (Cognitive Behavioral Therapies[Title/Abstract])) OR (Therapies, Cognitive Behavioral[Title/Abstract])) OR (Therapy, Cognitive Behavioral[Title/Abstract])) OR (Psychotherapy, Cognitive[Title/Abstract])) OR (Therapy, Cognitive[Title/Abstract])) OR (Cognitive Therapies[Title/Abstract])) OR (Therapies, Cognitive[Title/Abstract])) OR (Cognitive Therapy[Title/Abstract])) OR (Cognitive Behaviour Therapy[Title/Abstract])) OR (Behaviour Therapies, Cognitive[Title/Abstract])) OR (Behaviour Therapy, Cognitive[Title/Abstract])) OR (Cognitive Behaviour Therapies[Title/Abstract])) OR (Therapies, Cognitive Behaviour[Title/Abstract])) OR (Therapy, Cognitive Behaviour[Title/Abstract])) OR (Cognitive Psychotherapy[Title/Abstract])) OR (Cognitive Psychotherapies[Title/Abstract])) OR (Psychotherapies, Cognitive[Title/Abstract])) OR (Cognition Therapy[Title/Abstract])) OR (Cognition Therapies[Title/Abstract])) OR (Therapies, Cognition[Title/Abstract])) OR (Therapy, Cognitive

Behavior[Title/Abstract])) OR (Behavior Therapies, Cognitive[Title/Abstract])) OR (Cognitive Behavior Therapies[Title/Abstract])) OR (Therapies, Cognitive Behavior[Title/Abstract])) OR (Therapy, Cognition[Title/Abstract])) OR (Behavior Therapy, Cognitive[Title/Abstract])) OR (Cognitive Behavior Therapy[Title/Abstract]))) OR (group counselling[Title/Abstract])) OR (routine intervention[Title/Abstract])) OR (education intervention[Title/Abstract])) OR (((((((((((((((TS=(Telemedicine[Title/Abstract])) OR TS=(Tele-Referral[Title/Abstract])) OR TS=(Tele Referral[Title/Abstract])) OR TS=(Tele-Referrals[Title/Abstract])) OR TS=(Virtual Medicine[Title/Abstract])) OR TS=(Medicine, Virtual[Title/Abstract])) OR TS=(Tele-Intensive Care[Title/Abstract])) OR TS=(Tele Intensive Care[Title/Abstract])) OR TS=(Tele-ICU[Title/Abstract])) OR TS=(Tele ICU[Title/Abstract])) OR TS=(Mobile Health[Title/Abstract])) OR TS=(Health, Mobile[Title/Abstract])) OR TS=(mHealth[Title/Abstract])) OR TS=(Telehealth[Title/Abstract])) OR TS=(eHealth[Title/Abstract]))) OR (((TS=(Multi-level Counseling Program[Title/Abstract])) OR TS=(combined intervention[Title/Abstract])) OR TS=(mixed intervention[Title/Abstract]))) AND ((randomized controlled trial [pt] OR controlled clinical trial [pt] OR randomized [tiab] OR placebo [tiab] OR drug therapy [sh] OR randomly [tiab] OR trial [tiab] OR groups [tiab]) NOT (animals [mh] NOT humans [mh]))) AND ((("Internet Addiction Disorder"[Mesh]) OR ((Addiction Disorder, Internet[Title/Abstract]) OR (Addiction Disorders, Internet[Title/Abstract])) OR (Disorder, Internet Addiction[Title/Abstract])) OR (Disorders, Internet Addiction[Title/Abstract])) OR (Internet Addiction Disorders[Title/Abstract])) OR (Internet Addiction[Title/Abstract])) OR (Addiction, Internet[Title/Abstract])) OR (Addictions, Internet[Title/Abstract])) OR (Internet Addictions[Title/Abstract])) OR (Social Media Addiction[Title/Abstract])) OR (Addiction, Social Media[Title/Abstract])) OR (Addictions, Social Media[Title/Abstract])) OR (Media Addiction, Social[Title/Abstract])) OR (Media Addictions, Social[Title/Abstract])) OR (Social Media Addictions[Title/Abstract])) OR (Smartphone Addiction[Title/Abstract])) OR (Addiction, Smartphone[Title/Abstract])) OR (Addictions, Smartphone[Title/Abstract])) OR (Smartphone Addictions[Title/Abstract])) OR (Internet Gaming Disorder[Title/Abstract])) OR (Disorder, Internet Gaming[Title/Abstract])) OR (Disorders, Internet Gaming[Title/Abstract])) OR (Gaming Disorder, Internet[Title/Abstract])) OR (Gaming Disorders, Internet[Title/Abstract])) OR (Internet Gaming Disorders[Title/Abstract])) OR (problematic Internet use[Title/Abstract])) OR (internet use disorder[Title/Abstract])) OR (internet use[Title/Abstract])) OR (Internet Uses[Title/Abstract])) OR (Use, Internet[Title/Abstract])) OR (Web Usage[Title/Abstract])) OR (Usage, Web[Title/Abstract])) OR (Web Use[Title/Abstract])) OR (Use, Web[Title/Abstract])) OR (Internet Usage[Title/Abstract])) OR (Usage, Internet[Title/Abstract])) OR (Internet-addicted[Title/Abstract])) OR (Internet depend[Title/Abstract])) OR (Internet overuse[Title/Abstract])) OR (Compulsive internet[Title/Abstract])) OR (Pathological internet[Title/Abstract])) OR (Excessive internet[Title/Abstract])) OR (Addictive internet[Title/Abstract])) OR (Video game addiction[Title/Abstract])) OR (Online

game addiction[Title/Abstract])) OR (Cybersex addiction[Title/Abstract])) OR (Internet sex addiction[Title/Abstract])) OR (Social network addiction[Title/Abstract])) OR (Facebook addiction[Title/Abstract])))))

Embase Search Strategy

#11. #1 AND #2 AND #10 666
22 Dec 2023
#10. #3 OR #4 OR #5 OR #6 OR #7 OR #8 OR #9 4,386,272
22 Dec 2023
#9. 'psychology, positive'/exp OR 'psychology, 126,527
22 Dec 2023
positive' OR (('psychology,'/exp OR psychology,) AND positive) OR 'positive psychology':ti,ab,kw
#8. 'sandplay therapy' OR (sandplay AND 476
22 Dec 2023
('therapy'/exp OR therapy)) OR 'play therapies':ti,ab,kw OR 'therapies, play':ti,ab,kw OR 'therapy, play':ti,ab,kw OR 'sandplay therapy':ti,ab,kw OR 'sandplay therapies':ti,ab,kw OR 'therapies, sandplay':ti,ab,kw OR 'therapy, sandplay':ti,ab,kw OR sandplay:ti,ab,kw OR sandplays:ti,ab,kw
#7. 'family intervention'/exp OR 'family 654,623
22 Dec 2023
intervention' OR (('family'/exp OR family) AND ('intervention'/exp OR intervention)) OR counseling:ti,ab,kw OR mindfulness:ti,ab,kw OR acupuncture:ti,ab,kw OR nursing:ti,ab,kw
#6. 'virtual reality exposure therapy'/exp OR 3,381,661
22 Dec 2023
'virtual reality exposure therapy' OR (virtual AND ('reality'/exp OR reality) AND ('exposure'/exp OR exposure) AND ('therapy'/exp OR therapy)) OR 'virtual reality immersion':ti,ab,kw OR therapy:ti,ab,kw OR 'virtual reality therapy':ti,ab,kw OR 'reality therapies, virtual':ti,ab,kw OR 'reality therapy, virtual':ti,ab,kw OR 'therapies, virtual reality':ti,ab,kw OR 'therapy, virtual reality':ti,ab,kw OR 'virtual reality therapies':ti,ab,kw
#5. tdcs OR 'cathodal stimulation transcranial direct 10,906

22 Dec 2023

current stimulation':ti,ab,kw OR 'cathodal stimulation tdcs':ti,ab,kw OR 'cathodal stimulation tdcss':ti,ab,kw OR 'stimulation tdcs, cathodal':ti,ab,kw OR 'stimulation tdcss, cathodal':ti,ab,kw OR 'tdcs, cathodal stimulation':ti,ab,kw OR 'tdcss, cathodal stimulation':ti,ab,kw OR 'transcranial random noise stimulation':ti,ab,kw OR 'transcranial ternating current stimulation':ti,ab,kw OR 'transcranial electrical stimulation':ti,ab,kw OR 'electrical stimulation, transcranial':ti,ab,kw OR 'electrical stimulations, transcranial':ti,ab,kw OR 'stimulation, transcranial electrical':ti,ab,kw OR 'stimulations, transcranial electrical':ti,ab,kw OR 'transcranial electrical stimulations':ti,ab,kw OR 'anodal stimulation transcranial direct current stimulation':ti,ab,kw OR 'anodal stimulation tdcs':ti,ab,kw OR 'anodal stimulation tdcss':ti,ab,kw OR 'stimulation tdcs, anodal':ti,ab,kw OR 'stimulation tdcss, anodal':ti,ab,kw OR 'tdcs, anodal stimulation':ti,ab,kw OR 'tdcss, anodal stimulation':ti,ab,kw OR 'repetitive transcranial electrical stimulation':ti,ab,kw

#4. exercises OR 'physical activity':ti,ab,kw OR 353,862

22 Dec 2023

'activities, physical':ti,ab,kw OR 'activity, physical':ti,ab,kw OR 'physical activities':ti,ab,kw OR 'exercise, physical':ti,ab,kw OR 'exercises, physical':ti,ab,kw OR 'physical exercise':ti,ab,kw OR 'physical exercises':ti,ab,kw OR 'acute exercise':ti,ab,kw OR 'acute exercises':ti,ab,kw OR 'exercise, acute':ti,ab,kw OR 'exercises, acute':ti,ab,kw OR 'exercise, isometric':ti,ab,kw OR 'exercises, isometric':ti,ab,kw OR 'isometric exercises':ti,ab,kw OR 'isometric exercise':ti,ab,kw OR 'exercise, aerobic':ti,ab,kw OR 'aerobic exercise':ti,ab,kw OR 'aerobic exercises':ti,ab,kw OR 'exercises, aerobic':ti,ab,kw OR 'exercise training':ti,ab,kw

OR 'exercise trainings':ti,ab,kw OR 'training, exercise':ti,ab,kw OR 'trainings, exercise':ti,ab,kw OR football:ti,ab,kw OR 'tai chi':ti,ab,kw OR basketball:ti,ab,kw OR 'high-intensity interval training':ti,ab,kw OR 'high intensity interval training':ti,ab,kw OR 'high-intensity interval trainings':ti,ab,kw OR 'interval training, high-intensity':ti,ab,kw OR 'interval trainings, high-intensity':ti,ab,kw OR 'training, high-intensity interval':ti,ab,kw OR 'trainings, high-intensity interval':ti,ab,kw OR 'high-intensity intermittent exercise':ti,ab,kw OR 'exercise, high-intensity intermittent':ti,ab,kw OR 'exercises, high-intensity intermittent':ti,ab,kw OR 'high-intensity intermittent exercises':ti,ab,kw OR 'sprint interval training':ti,ab,kw OR 'sprint interval trainings':ti,ab,kw

#3. 'behavioral therapies, cognitive' OR (behavioral AND therapies, AND cognitive) OR 'behavioral therapy, cognitive':ti,ab,kw OR 'cognitive behavioral therapies':ti,ab,kw OR 'therapies, cognitive behavioral':ti,ab,kw OR 'therapy, cognitive behavioral':ti,ab,kw OR 'psychotherapy, cognitive':ti,ab,kw OR 'therapy, cognitive':ti,ab,kw OR 'cognitive therapies':ti,ab,kw OR 'therapies, cognitive':ti,ab,kw OR 'cognitive therapy':ti,ab,kw OR 'cognitive behaviour therapy':ti,ab,kw OR 'behaviour therapies, cognitive':ti,ab,kw OR 'behaviour therapy, cognitive':ti,ab,kw OR 'cognitive behaviour therapies':ti,ab,kw OR 'therapies, cognitive behaviour':ti,ab,kw OR 'therapy, cognitive behaviour':ti,ab,kw OR 'cognitive psychotherapy':ti,ab,kw OR 'cognitive psychotherapies':ti,ab,kw OR 'psychotherapies, cognitive':ti,ab,kw OR 'cognition therapy':ti,ab,kw OR 'cognition therapies':ti,ab,kw OR 'therapies, cognition':ti,ab,kw OR 'therapy, cognitive behavior':ti,ab,kw OR 'behavior therapies, cognitive':ti,ab,kw OR 'cognitive behavior 20,662

22 Dec 2023

therapies':ti,ab,kw OR 'therapies, cognitive behavior':ti,ab,kw OR 'therapy, cognition':ti,ab,kw OR 'behavior therapy, cognitive':ti,ab,kw OR 'cognitive behavior therapy':ti,ab,kw

#2.	'internet addiction disorder'/exp OR 'internet addiction disorder' OR (('internet'/exp OR internet) AND ('addiction'/exp OR addiction) AND ('disorder'/exp OR disorder)) OR 'addiction disorder, internet':ti,ab,kw OR 'addiction disorders, internet':ti,ab,kw OR 'disorder, internet addiction':ti,ab,kw OR 'disorders, internet addiction':ti,ab,kw OR 'internet addiction disorders':ti,ab,kw OR 'internet addiction':ti,ab,kw OR 'addiction, internet':ti,ab,kw OR 'addictions, internet':ti,ab,kw OR 'internet addictions':ti,ab,kw OR 'social media addiction':ti,ab,kw OR 'addiction, social media':ti,ab,kw OR 'addictions, social media':ti,ab,kw OR 'media addiction, social':ti,ab,kw OR 'media addictions, social':ti,ab,kw OR 'social media addictions':ti,ab,kw OR 'smartphone addiction':ti,ab,kw OR 'addiction, smartphone':ti,ab,kw OR 'addictions, smartphone':ti,ab,kw OR 'smartphone addictions':ti,ab,kw OR 'internet gaming disorder':ti,ab,kw OR 'disorder, internet gaming':ti,ab,kw OR 'disorders, internet gaming':ti,ab,kw OR 'gaming disorder, internet':ti,ab,kw OR 'gaming disorders, internet':ti,ab,kw OR 'internet gaming disorders':ti,ab,kw OR 'problematic internet use':ti,ab,kw OR 'internet use disorder':ti,ab,kw OR 'internet use':ti,ab,kw OR 'internet uses':ti,ab,kw OR 'use, internet':ti,ab,kw OR 'web usage':ti,ab,kw OR 'usage, web':ti,ab,kw OR 'web use':ti,ab,kw OR 'use, web':ti,ab,kw OR 'internet usage':ti,ab,kw OR 'usage, internet':ti,ab,kw OR 'internet addicted':ti,ab,kw OR 'internet depend':ti,ab,kw OR 'internet overuse':ti,ab,kw OR 'compulsive	15,402	22 Dec 2023

internet':ti,ab,kw OR 'pathological internet':ti,ab,kw OR 'excessive internet':ti,ab,kw OR 'addictive internet':ti,ab,kw OR 'video game addiction':ti,ab,kw OR 'online game addiction':ti,ab,kw OR 'cybersex addiction':ti,ab,kw OR 'internet sex addiction':ti,ab,kw OR 'social network addiction':ti,ab,kw OR 'facebook addiction':ti,ab,kw

#1. 'crossover procedure':de OR 'double-blind procedure':de OR 'randomized controlled trial':de OR 'single-blind procedure':de OR random*:de,ab,ti OR factorial*:de,ab,ti OR crossover*:de,ab,ti OR ((cross NEXT/1 over*):de,ab,ti) OR placebo*:de,ab,ti OR ((doubl* NEAR/1 blind*):de,ab,ti) OR ((singl* NEAR/1 blind*):de,ab,ti) OR assign*:de,ab,ti OR allocat*:de,ab,ti OR volunteer*:de,ab,ti 3,184,520
22 Dec 2023

Chinese BioMedical Literature Search Strategy

(cognitive behavioral therapy or behavioral therapy or exercise or exercise or physical activity or badminton or basketball or soccer exercise or Tai Chi or Baduanjin or high-intensity interval exercises or high-intensity interval training or aerobic exercise or resistance exercises or physical therapy or physical therapy or transcranial magnetic stimulation or electrical stimulation or family therapy or group intervention or family intervention or psychological therapy or counseling or positive thinking or acupuncture or nursing or positive psychotherapy or positive psychological intervention or sandplay or sandbox therapy or virtual reality therapy or reality therapy or hypnosis) and (Internet addiction or Internet addiction or gaming addiction or computer addiction or video game addiction or online gaming addiction or Internet addiction or transitional Internet access) and (random) 134 2023-12-23 23:04

China National Knowledge Infrastructure Search Strategy

Search formula A: Topic = cognitive behavioral therapy + behavioral therapy + sports + exercise + physical activity + badminton + basketball + soccer + sports exercise + tai chi + bada-jin + high intensity interval exercises + OR Topic = electrical stimulation + family therapy + group intervention + family intervention + psychotherapy + counseling + positive thinking + acupuncture + nursing + positive psychotherapy + positive psychological intervention + sandplay + sandplay therapy + virtual reality Therapy + Reality Therapy + Hypnosis + Sandplay + Sandplay OR Topic = Virtual

Reality Therapy + Reality Therapy + Hypnosis + Virtual Reality Techniques AND Topic = Internet Addiction + Internet Addiction + Gaming Addiction + Computer Addiction + Video Game Addiction + Online Game Addiction + Internet Addiction + Transitional Internet Access OR (Title = Cognitive Behavioral Therapy + Behavioral Therapy + Exercise + Exercise + Physical Activity + Badminton + Basketball + soccer + exercise + tai chi + bajuanjin + high intensity interval exercises + or title = electrical stimulation + family therapy + group intervention + family intervention + psychotherapy + counseling + positive thinking + acupuncture + nursing + positive psychotherapy + positive psychological intervention + sandplay + sandplay therapy + virtual reality therapy + reality therapy + hypnosis + sandplay + sandplay therapy or title = virtual reality therapy + reality therapy + Hypnosis + Virtual Reality Therapy or title = Internet Addiction + Internet Addiction + Game Addiction + Computer Addiction + Video Game Addiction + Online Game Addiction + Internet Addiction + Transitional Internet Access) (fuzzy match)

WanFang Search Strategy

(cognitive behavioral therapy or behavioral therapy or exercise or exercise or physical activity or badminton or basketball or soccer exercise or Tai Chi or Baduanjin or high-intensity interval exercises or high-intensity interval training or aerobic exercise or resistance exercises or physical therapy or physical therapy or transcranial magnetic stimulation or electrical stimulation or family therapy or group intervention or family intervention or psychological therapy or counseling or positive thinking or acupuncture or nursing or positive psychotherapy or positive psychological intervention or sandplay or sandbox therapy or virtual reality therapy or reality therapy or hypnosis) and (Internet addiction or Internet addiction or gaming addiction or computer addiction or video game addiction or online gaming addiction or Internet addiction or transitional Internet access) and (random)

China Science and Technology Journal Search Strategy

#1= Cognitive-behavioral therapy or behavioral therapy or exercise or exercise or physical activity or badminton or basketball or soccer exercise or Tai Chi or Baduanjin or high-intensity interval exercises or high-intensity interval training or aerobic exercise or resistance exercises or physical therapy or physical therapy or physical therapy or transcranial magnetic stimulation or electrical stimulation or family therapy or group intervention or family intervention or Psychotherapy or counseling or positive thinking or acupuncture or nursing or positive psychotherapy or positive psychological intervention or sandplay or sandbox therapy or virtual reality therapy or reality therapy or hypnosis
#2= Internet addiction or Internet addiction or gaming addiction or computer addiction or video game addiction or online game addiction or Internet addiction or transitioning to the Internet
#3= #1 and #2
