# Supplementary material for: Optimal Non-Pharmacological Interventions for Reducing Problematic Internet Use in Youth: A Systematic Review and Bayesian Network Meta-Analysis
Source: Behav Sci (Basel). 2025 Jan 20;15(1):98. doi: 10.3390/bs15010098 (PMC11762159; doi:10.3390/bs15010098)
Supplement: Supplementary file 1 [file behavsci-15-00098-s001.zip › Supplement Appendix S3. List of excluded studies.pdf]

List of Excluded Studies

Article Number	Literature Basic Information	Reason for exclusion
1	[1] MATSUDA Y, TAKEUCHI T, TAKAHASHI Y, et al. Development and evaluation of the e-learning teaching materials for surgical nursing training; proceedings of the 9th International Congress on Nursing Informatics, Seoul, SOUTH KOREA, F 2006.	Studies were not randomized controlled trial
2	[2] ZHANG CAIYU. Analysis of Psychological Issues and Group Counseling Intervention Measures for Internet Addiction in Middle School Students [M].	Studies without appropriate outcomes
3	[3] ZHANG GONG. Discussion on the Treatment Model of Internet Addiction Patients [M].	Studies were not randomized controlled trial
4	[4] AHN C-Y, LEE HYUNGCHO. A Study on the Development and Effectiveness of Cognitive-Behavioral Therapy for Internet Game Addiction [J]. Korean Journal of Health Psychology, 2002, 7(3): 463-86.	Studies were not report their available data
5	[5] COLBURN C M. Strategic interaction online: A comparison of instructional techniques to optimize the use of scenarios as a distance learning exercise on the Internet [M]. 2002.	Studies were not report their available data
6	[6] KYUNG K H, KWON J-H. The Effect of the Cognitive-Behavioral Group Therapy for High Risk Students of Internet Addiction [J]. Korean Journal of Clinical Psychology, 2002, 21(3): 503-14.	Studies were not report their available data
7	[7] LIANG ZHUPING. College Students' Internet Psychological Disorders and Group Counseling %J Journal of Guangxi University (Philosophy and Social Science Edition) [J]. 2003, (06): 101-5.	Studies were not randomized controlled trial
8	[8] ZHANG CAIXIA. Internet Addiction Behavior and Adjustment in Vocational College Students %J Journal of Nantong Textile Vocational and Technical College [J]. 2003, (04): 72-4.	Studies without appropriate outcomes
9	[9] ZHANG CHUNYAN. Causes of Internet Addiction and Psychotherapy %J Journal of Jimei University (Education Science Edition) [J]. 2003, (03): 70-2+86.	Studies were not report their available data
10	[10] CHEN QIUMING, WU YEGUANG. Psychotherapy for Children's Internet Addiction %J China Clinical Rehabilitation [J]. 2004, (18): 3473-85.	Studies were not report their available data

11	[11] SHAO ZHI, YANG RONG, LUO KANGLING, et al. Clinical Study of Psychotherapy for Students with Internet Addiction %J Chinese Journal of Child Health Care [J]. 2004, (06): 548-57.	Studies were not report their available data
12	[12] WU JIE, WANG YAN, YANG XIUFENG, et al. Analysis of the Causes of Internet Addiction in College Students and Discussion on Correction Methods %J Chinese Journal of School Health [J]. 2004, (04): 462-3.	Studies were not report their available data
13	[13] XU JUN. Analysis of the Current Situation of College Students' Internet Addiction and Research on Psychological Treatment and Prevention Strategies [D]; Wuhan University of Technology, 2004.	Studies were not report their available data
14	[14] YANG YANPING. Group Psychological Counseling for Internet Addiction Students [D]; East China Normal University, 2004.	Studies without appropriate outcomes
15	[15] GRIEBSCH I, COAST J, BROWN J. Quality-adjusted life-years lack quality in pediatric care: A critical review of published cost-utility studies in child health [J]. Pediatrics, 2005, 115(5): E600-E14.	Studies were not randomized controlled trial
16	[16] BAI YU, FAN FUMIN. College Students' Internet Dependence and Group Intervention Methods %J Youth Studies [J]. 2005, (05): 42-9+19.	Studies without appropriate outcomes
17	[17] DONG WEI. A New Attempt at Internet Addiction Syndrome: Drug Treatment Plus Psychological Intervention %J Mass Psychology [J]. 2005, (10): 18-9.	Studies were not report their available data
18	[18] GAO WENBIN, ZHU ZHUOHONG, CHEN ZHIYAN, et al. Study on the Psychological Mechanism of Internet Addiction and Comprehensive Psychological Intervention; proceedings of the 10th National Psychological Science Conference, Shanghai, China, F, 2005 [C].	Studies were not randomized controlled trial
19	[19] GONG YINQING, YANG RONG, ZHANG BIN. Psychological Treatment Methods and Techniques for Internet Addiction in Middle School Students %J Chinese Journal of School Health [J]. 2005, (05): 408-9.	Studies were not report their available data
20	[20] LIANG YINGNAN. A Review of Internet Addiction Issues %J Science and Technology Information [J]. 2005, (22): 139.	Studies were not report their available data
21	[21] YANG FANGRU, HAO WEI. Efficacy Observation of Psychological and Social Comprehensive Intervention in 52 Cases of Internet Addiction Adolescents %J Chinese Journal of Clinical Psychology [J]. 2005, (03): 343-5+52.	Studies were not report their available data

22	[22] YANG RONG, SHAO ZHI, ZHENG YONG. Comprehensive Intervention for Internet Addiction in Middle School Students %J Chinese Mental Health Journal [J]. 2005, (07): 25-7.	Studies were not report their available data
23	[23] YU YANZHI. The Feasibility of Group Psychological Intervention to Improve Adolescents' Internet Addiction Behavior %J China Clinical Rehabilitation [J]. 2005, 9(20).	Studies were not randomized controlled trial
24	[24] ZHANG GAOCHANG. The Preventive Effect of Positive Psychological Quality on College Students' Internet Addiction [D]; East China Normal University, 2005.	Studies without appropriate outcomes
25	[25] ZHU ZHUOHONG, GAO WENBIN, CHEN ZHIYAN, et al. Action Research on Treatment Programs for Adolescent Internet Addiction; proceedings of the 10th National Psychological Science Conference, Shanghai, China, F, 2005 [C].	Studies were not randomized controlled trial
26	[26] SAMPSON M A, MUNOZ-FURLONG A, SICHERER S H. Risk-taking and coping strategies of adolescents and young adults with food allergy [J]. Journal of Allergy and Clinical Immunology, 2006, 117(6): 1440-5.	Studies were not report their available data
27	[27] T. N C. - Efficacy and Safety of Three Insulin Protocols in Medical Intensive Care Unit Patients [J]. 2006.	Studies were not report their available data
28	[28] YAN C, YANG F. Internet Addiction Disorder Behavioral Traits and Family Education Methods in Adolescents [J]. Chinese Journal of Practical Pediatrics, 2006, 21(9): 701-3.	Studies were not randomized controlled trial
29	[29] AN MINBING. The Intervention of Social Work in Adolescents' Internet Addiction Issues %J Journal of China University of Mining and Technology (Social Science Edition) [J]. 2006, (01): 106-8.	Studies were not report their available data
30	[30] CHEN XI WU A ZHENG A YANG A. The Role of Cognitive Psychotherapy in the Treatment of Internet Addiction %J Shandong Psychiatry [J]. 2006, 19(4).	Studies were not report their available data
31	[31] CHEN YUANYUAN. Causes of Adolescent Internet Addiction and Prevention and Treatment %J Journal of Shanghai Youth Management Cadre Institute [J]. 2006, (1).	Studies were not randomized controlled trial
32	[32] FAN FUMIN, BAI YU. Group Counseling Research on College Students' Internet Dependence (Abstract); proceedings of the 2006 Academic Seminar of the Chinese Society of Social Psychology, Anhui, China, F, 2006 [C].	Studies without appropriate outcomes

33	[33] GAO WENBIN, CHEN ZHIYAN. Research on the Pathological Psychological Mechanism of Internet Addiction and Comprehensive Psychological Intervention %J Advances in Psychological Science [J]. 2006, (04): 596-603.	Studies were not report their available data
34	[34] LEI MING, CHEN XGUANG, LIU WEIYAN, et al. Group Prevention of College Students' Internet Addiction with Psychological Counseling %J Northwest Medical Education [J]. 2006, 14(03): 265-7.	Studies were not report their available data
35	[35] LIN ZHIXIONG, ZOU XIAOBO, XIE BO, et al. Combined Treatment of Psychological Drugs for Adolescent Internet Addiction %J Neural Disease and Mental Health [J]. 2006, (02): 127-9.	Studies were not report their available data
36	[36] NI XIAOLI, YAN HONG, CHEN SILU. College Students' Internet Addiction and Mental Health Education %J Chinese Journal of Behavioral Medicine Science [J]. 2006, (05): 409-10.	Studies were not randomized controlled trial
37	[37] RAN MIN. Analysis of the Causes of College Students' Internet Addiction and Intervention Measures [D]; Sichuan University, 2006.	Studies without appropriate outcomes
38	[38] YU XIANPING. An Empirical Study on the Correction of Middle School Students' Internet Addiction Based on Family Therapy [D]; Jiangxi Normal University, 2006.	Studies were not report their available data
39	[39] ZHANG FENGNING, ZHANG YIPING, ZOU JINSHAN. Adolescent Internet Addiction and Systemic Family Therapy %J Chinese General Practice [J]. 2006, (01): 48-50.	Studies were not report their available data
40	[40] ZHANG LINGFANG WANG A LV A. Nursing of Internet Addiction Syndrome %J Occupational and Health [J]. 2006, 22(22).	Studies without appropriate outcomes
41	[41] ZHANG YUNHONG. Psychological Analysis and Correction Research of Internet Addiction Adolescents [D]; Shaanxi Normal University, 2006.	Studies were not report their available data
42	[42] MCCONNON A, KIRK S F L, COCKROFT J E, et al. The Internet for weight control in an obese sample: results of a randomised controlled trial [J]. BMC health services research, 2007, 7.	Studies were not report their available data
43	[43] YOUNG K S. Cognitive behavior therapy with Internet addicts: Treatment outcomes and implications [J]. Cyberpsychology & Behavior, 2007, 10(5): 671-9.	Studies were not report their available data
44	[44] CAO FENGLIN. Research on the Psychological Mechanism, Functional Neuroimaging, and Group Psychological Intervention of Adolescent Internet Addiction [D]; Central South University,	Studies were not report their available data

	2007.	
45	[45] ZENG MEIHUA. Psychological Analysis and Psychotherapy Exploration of Adolescent Internet Addiction Syndrome %J Social Science Forum (Academic Research Edition) [J]. 2007, (11): 115-8.	Studies without appropriate outcomes
46	[46] HAN JISHENG WU A YAN A. The Therapeutic Effect of 2/100Hz Transcutaneous Acupoint Electrical Stimulation on 27 Cases of Adolescent Internet Addiction Syndrome %J Chinese Journal of Drug Dependence [J]. 2007, 16(1).	Studies were not randomized controlled trial
47	[47] JIANG WENQING. Study on the Characteristics and Comprehensive Intervention Effects of Internet Addiction in Middle School Students in Shanghai [D]; Shanghai Jiao Tong University, 2007.	Studies were not report their available data
48	[48] LIU CHULAI GUO A YIN A ZHOU A. Research on Psychological and Sports Intervention for Adolescent Internet Addiction %J Journal of Capital Institute of Physical Education [J]. 2007, 19(6).	Studies without appropriate outcomes
49	[49] YE XIAOQING, LAI MINZHEN, XIE BO. Drug Treatment and Psychological Intervention for Internet Addiction Patients %J Nursing Research [J]. 2007, (35): 3262-3.	Studies were not randomized controlled trial
50	[50] ZHANG HONGYING, ZHANG CHENGZHONG. Research on Psychological and Behavioral Intervention for Adolescent Internet Addiction %J Clinical Psychosomatic Diseases Magazine [J]. 2007, (02): 151-2+28.	Studies without appropriate outcomes
51	[51] ZHU JIAN, KONG KUANGJIAN. The Therapeutic Effect of Sandbox Therapy on Adolescent Internet Addiction; proceedings of the 5th Academic Seminar of the Chinese Mental Health Association, Nanchang, Jiangxi, China, F, 2007 [C].	Studies were not randomized controlled trial
52	[52] CARROLL K M, BALL S A, MARTINO S, et al. Computer-assisted delivery of cognitive-behavioral therapy for addiction: A randomized trial of CBT4CBT [J]. American Journal of Psychiatry, 2008, 165(7): 881-8.	Studies were not report their available data
53	[53] CHICTR-TRC. - Clinical Research of Acupuncture Combined Behavior Intervention on Teenagers with Internet Addiction [J]. 2008.	Studies were not report their available data
54	[54] GONG XIAOHUI YANG A. Exploration of Cognitive Behavioral Therapy and Integrated Chinese and Western Medicine Treatment for Internet Addiction %J Shi Zhen Guo Yi Guo Yao [J]. 2008, 19(4).	Studies were not report their available data

55	[55] LI NING. Research on the Comprehensive Intervention Model of Psychopharmaceuticals for Internet Addiction Disorder [D]; Second Military Medical University, 2008.	Studies were not randomized controlled trial
56	[56] LI ZUNQING LIU A. Evaluation of the Rehabilitation Effect of Psychological Intervention on Inpatient Internet Addiction Patients %J Chinese Journal of School Health [J]. 2008, 29(12).	Studies without appropriate outcomes
57	[57] LIU CUI, MENG XIANGKANG, ZHENG BIYUN, et al. Educational Intervention Research on College Students with Internet Addiction %J Pharmaceutical Education [J]. 2008, (03): 1-4.	Studies were not report their available data
58	[58] QU YAN. Adolescent Internet Addiction and Group Counseling Strategies %J Journal of Sichuan University of Arts and Sciences [J]. 2008, (05): 83-4.	Studies were not report their available data
59	[59] WEI QIUXIANG. The Role of Psychological Nursing Intervention in the Treatment of Internet Addiction Patients [J]. 2008, (3 %J Journal of Heze Medical College).	Studies without appropriate outcomes
60	[60] WU SHAOLAN, YANG YONGXIN. Efficacy Analysis of Psychological Cognitive Nursing on the Recovery of Internet Addiction Patients' Character %J Chinese Medical Herald [J]. 2008, (23): 161-2.	Studies without appropriate outcomes
61	[61] XU GUANGRONG. Group Counseling Reality Therapy for College Students' Internet Addiction %J Chinese Journal of Health Psychology [J]. 2008, (06): 709-11.	Studies were not report their available data
62	[62] YOU YUJING. Research on the Nursing Intervention Measures for Parents' Child-rearing Styles and Internet Addiction Patients %J Qilu Nursing Journal [J]. 2008, (21): 5-6.	Studies were not report their available data
63	[63] ZHONG XIN, TAO RAN, YANG FENGCHI. Research on Psychological Intervention of Internet Addiction %J Capital Medical University Journal [J]. 2008, (05): 569-72.	Studies were not randomized controlled trial
64	[64] ZHU BIYA, WANG LIYAN. Positive Psychology Perspective on the Prevention and Treatment of Adolescent Internet Addiction %J Software Guide (Education Technology) [J]. 2008, (06): 44-5.	Studies were not report their available data
65	[65] MACKERT M, KAHLOR L, TYLER D, et al. Designing e-Health Interventions for Low-Health-Literate Culturally Diverse Parents: Addressing the Obesity Epidemic [J]. Telemedicine Journal and E-Health, 2009, 15(7): 672-7.	Studies without appropriate outcomes
66	[66] ZHU T-M, JIN R-J, ZHONG X-M. Clinical effect of electroacupuncture combined with psychologic interference on patient with Internet addiction disorder [J]. Zhongguo Zhong xi yi jie he za	Studies were not randomized controlled trial

	zhi Zhongguo Zhongxiyi jiehe zazhi = Chinese Journal of Integrated Traditional and Western Medicine, 2009, 29(3): 212-4.	
67	[67] CAO YURONG. The Role of Comprehensive Family Intervention in the Treatment of Internet Addiction %J Journal of Psychiatry [J]. 2009, 22(1): 48-9.	Studies were not report their available data
68	[68] DAN YUJIN LIU A. Exercise Psychology Perspective on the Psychological Attribution and Intervention of Adolescent Internet Addiction %J Journal of Beijing Sport University [J]. 2009, 32(8).	Studies without appropriate outcomes
69	[69] JIA FUCHI LI A CHEN A ZHANG A. The Impact of Fitness Exercise Prescription on the Intervention Effects of College Students' Internet Addiction %J Sports Science Journal [J]. 2009, 16(8).	Studies were not randomized controlled trial
70	[70] LU YAN. The Psychological Impact of Nursing Intervention on Inpatient Internet Addiction Adolescents' Anxiety [J]. 2009, 11(4 %J Southwest Military Medicine).	Studies without appropriate outcomes
71	[71] WANG HEYAN, MEI JUNAN. The Impact of Family Intervention on the Recovery of Internet Addicts [J]. 2009, 21(20 %J Chinese Journal of Civil Health).	Studies were not report their available data
72	[72] YU QING, YU LAN. Evaluation of the Correction Effect on the Mood and Social of Inpatient Internet Addiction Patients [J]. 2009, 28(1 %J International Nursing Journal).	Studies were not report their available data
73	[73] ZHAN LAIYING, LI RONGQIN. The Role of Introspection Cognitive Therapy in the Psychological Recovery of Internet Addiction Patients %J Nursing Journal [J]. 2009, 24(15): 77-9.	Studies were not report their available data
74	[74] ZHAO RUI ZHANG A. Research on the Intervention of Outdoor Expansion Sports for Adolescent Internet Addiction %J Inner Mongolia Sports Science and Technology [J]. 2009, (2).	Studies were not report their available data
75	[75] ZHONG XIN, TAO RAN, ZU SI, et al. Research on the Effect of Group Psychological Intervention on Adolescent Internet Addiction [J]. 2009, 30(4 %J Capital Medical University Journal).	Studies were not report their available data
76	[76] SON TAEHONG. The Effect of Cognitive-Behavioral Therapy on Youth's Excessive Internet Uses Based on Single System Design: An Exploratory Research to Develop a Home Intervention Program [J]. 2009, 11(2): 145-72.	Studies without appropriate outcomes
77	[77] ANDERSSON G. The promise and pitfalls of the internet for cognitive behavioral therapy [J]. BMC medicine, 2010, 8.	Studies were not report their available data

78	[78] HERCBERG S, CASTETBON K, CZERNICHOW S, et al. The Nutrinet-Sante Study: a web-based prospective study on the relationship between nutrition and health and determinants of dietary patterns and nutritional status [J]. BMC public health, 2010, 10.	Studies were not report their available data
79	[79] HUANG X Q, LI M C, TAO R. Treatment of internet addiction [J]. Current Psychiatry Reports, 2010, 12(5): 462-70.	Studies without appropriate outcomes
80	[80] CHEN LING. The Application of Cognitive Behavioral Therapy in the Prevention of Internet Addiction %J Social Work [J]. 2010, (11).	Studies were not randomized controlled trial
81	[81] CHEN XIUHONG. Family Intervention Research on Adolescent Internet Addiction Issues - From the Perspective of Family Function Theory %J Journal of Shanghai Youth Management Cadre Institute [J]. 2010, (01): 54-6.	Studies without appropriate outcomes
82	[82] GAO WENBIN, CHEN ZHIYAN. Efficacy Assessment of Group Therapy for Internet Addiction Adolescents; proceedings of the 12th China Association for Science and Technology Annual Meeting, Fuzhou, Fujian, China, F, 2010 [C].	Studies were not randomized controlled trial
83	[83] MIYAHARA HIROAKI, WANG XIAOMIN, YE JIQUN, et al. Evaluation of Family Therapy Outcomes for Adolescent Internet Addiction %J Chinese Journal of School Health [J]. 2010, 31(03): 300-1.	Studies were not report their available data
84	[84] GONG XIAOTAO ZHENG A ZHANG A. Electroacupuncture Combined with Behavioral Therapy for 38 Cases of Internet Addiction %J Shanghai Journal of Acupuncture and Moxibustion [J]. 2010, 29(2).	Studies without appropriate outcomes
85	[85] HOU CHUNYU, YAN TINGSHAN, ZHENG PENG. Exploration of Group Counseling Intervention for Vocational College Students with Internet Addiction %J China Science and Technology Information [J]. 2010, (21): 224-5.	Studies without appropriate outcomes
86	[86] LI JIANMING, CHU GUORONG, ZHANG FUAN, et al. Clinical Characteristics and Comprehensive Intervention of 108 Cases of Adolescent Internet Addiction %J Journal of Metallurgical Industry Medicine [J]. 2010, 27(04): 454-5.	Studies were not report their available data

87	[87] LIAO XIAOCHUN. Efficacy Analysis of Comprehensive Intervention Treatment for 142 Cases of Middle School Students with Internet Addiction [J]. 2010, 17(6 %J Practical Preventive Medicine).	Studies were not report their available data
88	[88] TAO RAN. Group Psychotherapy for Adolescent Internet Addiction; proceedings of the 1st National Academic Seminar on Internet Addiction, Beijing, China, F, 2010 [C].	Studies were not randomized controlled trial
89	[89] WU YINGDUO, GAO CAIFENG, WU CHUNRONG. Stigma-free Psychological Nursing Intervention to Promote the Recovery of Internet Addiction Patients [J]. 2010, (12 %J Nursing Journal).	Studies without appropriate outcomes
90	[90] YU XUELI, SHI JING. Causes of Middle School Students' Internet Addiction and Intervention %J Journal of Ningbo Institute of Education [J]. 2010, 12(03): 67-9.	Studies were not report their available data
91	[91] ZHANG GUIFENG, MA HAO, LIU YANGYANG, et al. Clinical Study on the Intervention of Acupuncture Combined with Cognitive Behavioral Therapy for Adolescent Internet Addiction %J Chinese Journal of Traditional Chinese Medicine and Pharmacy [J]. 2010, 28(12).	Studies were not report their available data
92	[92] ZHANG JIANGLI. The Effect of Drug and Psychological Comprehensive Treatment for Internet Addiction Children [J]. 2010, (12 %J Practical Pediatrics Clinic Medicine).	Studies were not report their available data
93	[93] ZHAO HUI. Research on the Intervention of Sports Activities for College Students' Internet Addiction %J Science and Technology Information [J]. 2010, (31).	Studies without appropriate outcomes
94	[94] ZHAO JING. Exploration and Research on the Social Psychological Factors of College Students' Internet Addiction and Group Psychological Intervention [D]; Shanxi Medical University, 2010.	Studies were not randomized controlled trial
95	[95] ZHENG HONGLI, YIN HAILAN. Research on Family Intervention for Adolescent Internet Dependence %J Journal of Qingdao Vocational and Technical College [J]. 2010, 23(03): 59-63.	Studies were not report their available data
96	[96] GOLSTEIJN C, VAN DEN HOVEN E. Facilitating communication about books through an online community [J]. Personal and Ubiquitous Computing, 2011, 15(2): 197-217.	Studies were not report their available data
97	[97] LIU Q X. Internet addiction disorder among Chinese adolescents: the effect of parent-adolescent interaction and need satisfaction and family group therapy [J]. 2011.	Studies without appropriate outcomes
98	[98] LOU Y T. An Empirical Study on The Intervention Effect of Physical Exercise Prescription on Teenage Internet Addiction; proceedings of the 2nd International Conference on Education and Sports Education, Guangzhou, PEOPLES R CHINA, F 2011 Apr 17-18, 2011 [C]. 2011.	Studies were not randomized controlled trial

99	[99] MORROW J R, JR., BAIN T M, FRIERSON G M, et al. Long-Term Tracking of Physical Activity Behaviors in Women: The WIN Study [J]. Medicine and science in sports and exercise, 2011, 43(1): 165-70.	Studies without appropriate outcomes
100	[100] OH H, LEE Y, KIM KYUNGMIN. The Effect of Residential Treatment for Adolescents with Severe Internet Addiction [J]. Asian Journal of Education, 2011, 12(2): 207-39.	Studies were not report their available data
101	[101] PARR J M, KAVANAGH D J, YOUNG R M, et al. Acceptability of cognitive-behaviour therapy via the Internet for cessation of benzodiazepine use [J]. Drug and Alcohol Review, 2011, 30(3): 306-14.	Studies were not report their available data
102	[102] SON C, JEONG EUNSIL. Effects of Mindfulness-Based Cognitive Therapy on Internet Addiction Level, Anxiety, and Stress in College Students with Internet Addiction [J]. Korean Journal of Clinical Psychology, 2011, 30(4): 825-43.	Studies were not report their available data
103	[103] YANG C, JEONG YONSEON. A Study on High School Students Actual Condition for Internet Addiction, Obesity Levels and Physical Fitness [J]. Korean Society for Wellness, 2011, 6(3): 241-52.	Studies without appropriate outcomes
104	[104] YUEQIN H, XINQIAO Z, ZHAORUI L I U. Community intervention of health education to internet addiction in high school students [J]. Chinese Mental Health Journal, 2011, 25(5): 328-31.	Studies were not report their available data
105	[105] ZHU T, LI H, DU Y, et al. Effect of Electro-acupuncture and Psychological Intervention on the Cerebral Working Ability of Internet Addiction Disorder Patients [J]. Journal of Traditional Chinese Medicine, 2011, 52(19): 1647-50.	Studies were not report their available data
106	[106] ZHU T-M, LI H, DU Y-P, et al. Intervention on network craving and encephalofluctuogram in patients with internet addiction disorder: a randomized controlled trial [J]. Zhongguo zhen jiu = Chinese acupuncture & moxibustion, 2011, 31(5): 395-9.	Studies without appropriate outcomes
107	[107] CAI XIAOWU, CHEN ZHONGSHAN. Psychological Intervention and Prevention of College Students' Internet Addiction from the Perspective of Counselors %J Changsha University Journal [J]. 2011, 25(01): 127-8.	Studies were not report their available data
108	[108] GUO HUA. Outdoor Sports Intervention for College Students ' Internet Addiction %J China Foreign Investment [J]. 2011, (14).	Studies without appropriate outcomes

109	[109] JIA FUCHI LI A CHEN A LI A. Evaluation of the Intervention Effect of Basketball Exercise Prescription on Middle School Students with Internet Addiction %J Chinese Journal of School Health [J]. 2011, 32(5).	Studies were not randomized controlled trial
110	[110] LIU XUELAN, LI LIZHEN, HUANG XUEMEI. Application of Family Therapy in the Intervention of Adolescent Internet Addiction %J Journal of South China Normal University (Social Science Edition) [J]. 2011, (03): 71-6+160.	Studies were not report their available data
111	[111] LONG XIAOQUAN LONG A. Empirical Study on the Intervention of Sports Activities for College Students' Internet Addiction %J Journal of Guizhou Minzu College for Nationalities: Philosophy and Social Science Edition [J]. 2011, (4).	Studies were not report their available data
112	[112] MA NANZHEN, HE CHENGSEN. A Case-Control Study on Cognitive Behavioral Therapy for College Students' Tendency towards Internet Addiction [J]. 2011, 15(6 %J Chinese Journal of Disease Control and Prevention).	Studies without appropriate outcomes
113	[113] QI XIANGQIAN. Research on the Treatment of Adolescent Internet Addiction through Sports Activities %J Xiangzhou: Theory Edition [J]. 2011, (8).	Studies were not randomized controlled trial
114	[114] SHI LAN, TANG SHICHENG, WANG YUDONG. Research on College Students' Internet Addiction at Hunan University %J China Health Education [J]. 2011, 27(10).	Studies without appropriate outcomes
115	[115] SU PEIZHU, ZHANG GUIFENG, LIU YANGYANG, et al. Acupuncture Combined with Behavioral Therapy for Depressive Symptoms in Internet Addiction Individuals [J]. 2011, 38(3 %J Liaoning Journal of Traditional Chinese Medicine).	Studies were not report their available data
116	[116] WANG CHANGHAI, GUAN QINGHUA. Research on Psychological Intervention for College Students' Internet Addiction %J Education and Occupation [J]. 2011, (09): 98-9.	Studies were not report their available data
117	[117] ZHANG CHENGAI. The Psychological Impact of Nursing Intervention on Inpatient Internet Addiction Adolescents [J]. 2011, 33(3 %J Journal of Shandong Medical College).	Studies without appropriate outcomes
118	[118] ZHANG YING, NING LI, WANG YING. Psychological Nursing Intervention for Inpatient Internet Addiction Adolescents [J]. 2011, 17(8 %J Chinese Modern Nursing Magazine).	Studies without appropriate outcomes

119	[118] ZHOU DEZHAO. Clinical Study on the Treatment of Internet Addiction Syndrome with Acupuncture and Auricular Pressure [D]; Guangzhou University of Chinese Medicine, 2011.	Studies were not randomized controlled trial
120	[119] ZHOU WENTING. Research on the Role of Sports Activities in the Intervention of Adolescent Internet Addiction %J Henan Science and Technology [J]. 2011, 0(11X).	Studies were not report their available data
121	[120] GUAN M, QIAN S, LIU W. Effect of Group Therapy on Higher Vocational School Students with Internet Dependence [J]. Chinese General Practice, 2012, 15(12A): 4000-2.	Studies were not report their available data
122	[121] JAEGER S, MUELLER K W, RUCKES C, et al. Effects of a manualized short-term treatment of internet and computer game addiction (STICA): study protocol for a randomized controlled trial [J]. Trials, 2012, 13.	Studies without appropriate outcomes
123	[122] KWON K, PARK S-M, JEON EUN YOUNG, et al. The Development and Effects of Parents-Child Participatory Group Counseling Programs for the Prevention of Elementary School Age Internet Addition [J]. The Korea Journal of Youth Counseling, 2012, 20(2): 57-85.	Studies were not report their available data
124	[123] MIN J. The Effect of Group Counseling Program for the Internet Addiction and Cyber Delinquent Children Based on Adlerian Therapy [J]. Korean Journal Of Counseling And Psychotherapy, 2012, 24(3): 533-53.	Studies were not report their available data
125	[124] YEH Y-C, LIN S S J, TSENG Y-H, et al. THE QUESTIONNAIRE OF LIFESTYLE CHANGE IN REGARD TO PROBLEMATIC INTERNET USE: FACTOR STRUCTURE AND CONCURRENT AND CROSS-YEAR PREDICTIVE UTILITIES [J]. Turkish Online Journal of Educational Technology, 2012, 11(4): 315-24.	Studies without appropriate outcomes
126	[125] ZHU T-M, LI H, JIN R-J, et al. Effects of Electroacupuncture Combined Psycho-intervention on Cognitive Function and Event-Related Potentials P300 and Mismatch Negativity in Patients with Internet Addiction [J]. Chinese Journal of Integrative Medicine, 2012, 18(2): 146-51.	Studies were not report their available data
127	[126] CAO HONGQING. The Impact of Physical Exercise on High School Students' Internet Addiction %J Inner Mongolia Sports Science and Technology [J]. 2012, 25(2).	Studies without appropriate outcomes
128	[127] JI FANG. Nursing Intervention on the Anxiety of Inpatient Internet Addiction Adolescents and Its Psychological Impact [J]. 2012, 34(2 %J Journal of Shandong Medical College).	Studies were not report their available data

129	[128] LONG LIN ZHOU A LIU A. Research on the Intervention Strategies of Sports Activities for College Students with Internet Addiction %J Sports Science Literature Bulletin [J]. 2012, 20(12).	Studies were not report their available data
130	[129] QIAN LONGCHAO. The Role of Table Tennis Exercise Intervention in Correcting Adolescent Internet Addiction [D]; Guangzhou Sport University, 2012.	Studies were not report their available data
131	[130] TIAN YINGCHUAN CHAI A XIAO A. Experience with Cognitive Behavioral Therapy Intervention for 32 Cases of Internet Addiction in New Soldiers %J Journal of Logistics University of People's Armed Police Force: Medical Edition [J]. 2012, 21(8).	Studies without appropriate outcomes
132	[131] WANG QIAN. Research on the Current Situation and Intervention of College Students' Internet Addiction [D]; Shanghai Normal University, 2012.	Studies were not report their available data
133	[132] ZHAO JING XIE A QI A ZHANG A. The Feasibility of Group Psychological Intervention Methods to Improve Behavior of Medical Students with Internet Addiction %J Chinese School Doctor [J]. 2012, 26(11).	Studies were not report their available data
134	[133] ZHONG MIAO LI A. Review and Research on the Intervention of Sports Activities for Internet Addiction %J Sports Science Literature Bulletin [J]. 2012, 20(10).	Studies were not report their available data
135	[134] JADAD A R. On Living a Long, Healthy, and Happy Life, Full of Love, and with no Regrets, until Our Last Breath [J]. Verhaltenstherapie, 2013, 23(4): 287-9.	Studies without appropriate outcomes
136	[135] LI H, LU L. THE ROLE OF COGNITIVE DISTORTION IN ONLINE GAME ADDICTION AMONG CHINESE ADOLESCENTS [J]. Children and Youth Services Review, 2013, 35(9): 1468-75.	Studies were not report their available data
137	[136] WEITZMAN E R, KELEMEN S, QUINN M, et al. Participatory Surveillance of Hypoglycemia and Harms in an Online Social Network [J]. JAMA internal medicine, 2013, 173(5): 345-51.	Studies without appropriate outcomes
138	[137] WOELFLING K, LEMENAGER T, PEUKERT P, et al. Computer games and internet addiction as well as pathological gambling. Therapy approaches [J]. Der Nervenarzt, 2013, 84(5): 576-83.	Studies were not report their available data
139	[138] ZIMMER B. Process and Outcome of an Internet-Based Preparation for Inpatient Psychosomatic Psychotherapeutic Treatment: An Examination of Website Use [J]. Psychiatrische Praxis, 2013, 40(6): 347-53.	Studies were not report their available data

140	[139] FU HONGYING. Psychological Intervention and Prevention Countermeasures for College Students' Internet Addiction %J Journal of Chongqing Second Normal College [J]. 2013, 26(02): 133-5.	Studies were not report their available data
141	[140] JIANG CUI. Discussion on the Current Situation and Intervention Research of College Students' Internet Addiction %J Journal of Chifeng College (Natural Science Edition) [J]. 2013, 29(12): 110-2.	Studies without appropriate outcomes
142	[141] LI QIZHEN. Research on the Intervention of Sports Activities for the Prevention and Treatment of Adolescent Internet Addiction %J Contemporary Sports Science and Technology [J]. 2013, 3(8).	Studies were not randomized controlled trial
143	[142] LU YANHUI, SU YING, MU JIANGBING, et al. The Impact of Systematic Health Education on the Family Int imacy and Adaptability of Patients with Internet Addiction Syndrome [J]. 2013, 29(22 %J Chinese Journal of Practical Nursing).	Studies without appropriate outcomes
144	[143] MU HONGWEI. Research on the Intervention Effects of Group Sandplay Therapy for College Students with Internet Dependence %J Journal of Jiamusi Normal College [J]. 2013, (05): 337.	Studies without appropriate outcomes
145	[144] QI YULONG, WANG LAN, MA YAN, et al. Research on the Intervention of College Students' Internet Addiction Behavior [J]. 2013, 38(11 %J Journal of Bengbu Medical College).	Studies were not report their available data
146	[145] WANG MENG, LIU BINGLUN, LI RENJUN, et al. Evaluation of Psychological Intervention Effects on Middle School Students with Internet Addiction in Jinan %J Chinese Journal of School Health [J]. 2013, 34(9).	Studies without appropriate outcomes
147	[146] WU HONGXIN, QIAO DONG DONG, ZHAO CHAOHUI. Comparative Study on the Improvement of Symptoms in Internet Addiction Patients with Psychological Intervention [J]. 2013, 26(2 %J Journal of Psychiatry).	Studies were not report their available data
148	[147] WU XIANHUA. Research on the Characteristics of Interpersonal Relationships and Comprehensive Intervention for Adolescents with Internet Addiction [D]; Huazhong University of Science and Technology, 2013.	Studies were not report their available data
149	[149] YAN HUILING. A Brief Analysis of the Intervention of Expansion Sports for College Students' Internet Addiction %J Contemporary Sports Science and Technology [J]. 2013, 3(6).	Studies were not report their available data
150	[150] ZHANG CHAO, LIU DONGSHENG, DAN MENG, et al. Research on the Effect of Sports Intervention for Internet Addiction in Medical College Students [J]. 2013, (2 %J Chinese Higher	Studies without appropriate outcomes

	Medical Education).	
151	[151] ZHANG JIYAN. Study on the Correction of College Students' Internet Game Addiction from the Perspective of Positive Psychology %J Journal of Guangxi Normal University for Nationalities [J]. 2013, 30(04): 144-7.	Studies were not randomized controlled trial
152	[152] ZHANG YING, LIANG WENWEN. Nursing Intervention for Depressive Symptoms in Inpatient Internet Addiction Adolescents [J]. 2013, 10(07 %J Nursing Practice and Research).	Studies were not report their available data
153	[153] ZHENG CHENG, ZHU JIANYU, LI MING. The Effect of Neurolinguistic Programming Group Counseling on College Students' Tendency towards Internet Addiction [J]. 2013, 21(9 %J Chinese Journal of Health Psychology).	Studies were not report their available data
154	[154] AJAY KRISHNA G S, KODALI M, KRISHNAMOHAN P, et al. Effect of cognitive behavior therapy for problematic internet usage [J]. Indian Journal of Psychiatry, 2014, 56: S68.	Studies were not report their available data
155	[155] DONG G, LIN X, ZHOU H, et al. Cognitive flexibility in internet addicts: fMRI evidence from difficult-to-easy and easy-to-difficult switching situations [J]. Addictive behaviors, 2014, 39(3): 677-83.	Studies without appropriate outcomes
156	[156] DONG G, POTENZA M N. A cognitive-behavioral model of Internet gaming disorder: Theoretical underpinnings and clinical implications [J]. Journal of psychiatric research, 2014, 58: 7-11.	Studies were not report their available data
157	[157] KALAITZAKI A E, BIRTCHNELL J. The impact of early parenting bonding on young adults' Internet addiction, through the mediation effects of negative relating to others and sadness [J]. Addictive behaviors, 2014, 39(3): 733-6.	Studies were not report their available data
158	[158] KIM S M, HAN D H. Virtual reality therapy for internet gaming disorder [J]. Alcohol and Alcoholism, 2014, 49: i19.	Studies were not report their available data
159	[159] KING D L, DELFABBRO P H. Internet Gaming Disorder Treatment: A Review of Definitions of Diagnosis and Treatment Outcome [J]. Journal of Clinical Psychology, 2014, 70(10): 942-55.	Studies without appropriate outcomes
160	[160] SU Y, DONG J, LU Y. Persistent nursing interventions for patients with Internet addiction [J]. Journal of Nursing Science, 2014, 29(23): 61-5.	Studies were not report their available data

161	[161] WARTBERG L, THOMSEN M, MOLL B, et al. Pilot Study on the Effectiveness of a Cognitive Behavioural Group Programme for Adolescents with Pathological Internet Use [J]. Praxis der Kinderpsychologie und Kinderpsychiatrie, 2014, 63(1): 21-35.	Studies were not report their available data
162	[162] WEI Q, YANG Y, WANG C, et al. An intervention program for both adolescents with Internet addiction disorder and their parents [J]. Journal of Nursing Science, 2014, 29(19): 68-71.	Studies were not report their available data
163	[163] DI YUN, HU SULI. The Application of Modern Psychological Counseling Comprehensive Therapy in College Students' Internet Addiction %J Knowledge Economy [J]. 2014, (17): 69-70.	Studies without appropriate outcomes
164	[164] HUANG YUBIN. The Intervention Study of Group Painting Activity on College Students' Internet Addiction [Z]. Proceedings of the 22nd Academic Annual Meeting of Shanghai University Psychological Counseling Association. Shanghai. 2014: 54-60	Studies were not report their available data
165	[165] JI LIU ZHANG A. The Role of Sports Intervention in the Treatment of Adolescent Internet Addiction %J Ideological and Political Course Research [J]. 2014, 0(5).	Studies were not report their available data
166	[166] SU YING, DONG JUNLING, LU YANHUI. Continuous Comprehensive Nursing Intervention for Patients with Internet Addiction Syndrome [J]. 2014, 29(23 %J Nursing Journal).	Studies without appropriate outcomes
167	[167] WANG DONGPING, WEI SHIQIANG, ZHAN HEQIN. The Efficacy of Psychological Intervention Combined with Medication for Children with Internet Gaming Dependence [J]. 2014, 29(15 %J Chinese Journal of Practical Pediatrics).	Studies were not report their available data
168	[168] XU LINGZHONG MING A QI A ZHAO A RONG A. Evaluation of the Effect of Cognitive Behavioral Therapy Intervention on the Behavior of Medical Students with Internet Addiction %J Journal of Bengbu Medical College [J]. 2014, 39(2).	Studies were not report their available data
169	[169] ZHANG GUOPENG. The Effect of Physical Exercise Intervention on the Treatment of Middle School Students' Internet Addiction %J Science and Education Forum [J]. 2014, (5).	Studies were not report their available data
170	[170] ZHAO YANQING. The Application of Group Counseling Based on Positive Psychology Theory in the Intervention of College Students' Internet Dependence %J Science and Technology Education Guide [J]. 2014, (17): 227-8.	Studies without appropriate outcomes

171	[171] ZHU ZUDE SHI A YANG A. The Neuromechanism and Intervention Research of Internet Addiction %J Industry and Science Forum [J]. 2014, 0(12).	Studies were not report their available data
172	[172] DONG G, LIN X, POTENZA M N. Decreased functional connectivity in an executive control network is related to impaired executive function in Internet gaming disorder [J]. Progress in neuro-psychopharmacology & biological psychiatry, 2015, 57: 76-85.	Studies were not report their available data
173	[173] MOK J Y, WOO J M, WEON K K, et al. The Impact of Forest Therapy on Neuro-cognitive, Psychosocial, and Physiological Aspects of Adolescent Internet Addiction Risk Group [J]. Journal of Korean Society of Forest Science, 2015, 104(2): 277-84.	Studies were not report their available data
174	[174] T. N C. - Clinical Research of Electro-acupuncture Combined Psychological Intervention on Internet Addiction Disorder [J]. 2015.	Studies without appropriate outcomes
175	[175] AI LISA. Research on the Intervention of Internet Addiction among Russian College Students [D]; Tianjin University, 2015.	Studies were not report their available data
176	[176] DAI YANGHUI. Positive Psychological Intervention for Adolescent Internet Addiction %J Youth Magazine [J]. 2015, (04): 22-5.	Studies without appropriate outcomes
177	[177] JIA FUCHI LI A CHEN A HU A CUI A. Evaluation of the Intervention Effect of High-Intensity Interval Training and Diet Intervention on Obese Female College Students with Internet Addiction %J Chinese Journal of School Health [J]. 2015, 36(10).	Studies were not report their available data
178	[178] LI MINGCHAO. Analysis of the Effect of Psychological Counseling in the Treatment of Adolescent Internet Addiction %J Baotou Medical College Journal [J]. 2015, 31(04): 101-2.	Studies were not report their available data
179	[179] LI RENJUN, LIU BINGLUN, WANG HONGJUN, et al. Follow-up Study on the Intervention of Internet Addiction in Middle School Students in Jinan; proceedings of the 13th National Academic Conference of the Chinese Medical Association, Jinan, Shandong, China, F, 2015 [C].	Studies were not randomized controlled trial
180	[180] LIU XUEJUN. Exploration and Prospect of Acupuncture and Acupuncture Combined with Behavioral Treatment in the Intervention of Internet Addiction; proceedings of the 14th Academic Annual Meeting of the Mental Disorders Committee of the China Society of Integrative Medicine, Changsha, Hunan, China, F, 2015 [C].	Studies without appropriate outcomes

181	[181] WEN CHAO, DAI YU. Clinical Intervention Research on Adolescent Internet Addiction with Acupuncture Combined with Cognitive Behavioral Therapy [Z]. Proceedings of the 2015 Annual Conference of the China Acupuncture Society. Shanghai. 2015: 282-3	Studies without appropriate outcomes
182	[182] XIA CUI CUI, ZHANG JIMING, WANG DONGSHENG. Application of Cognitive Behavioral Therapy in the Group Intervention of College Students' Internet Addiction; proceedings of the 30th Anniversary Memorial Activity and the 8th National Academic Conference of the Chinese Mental Health Association, Beijing, China, F, 2015 [C].	Studies were not report their available data
183	[183] YAN WEI. Psychological Intervention and Prevention Strategy for College Students' Internet Addiction from the Perspective of Counselors %J Seeking Knowledge Guide [J]. 2015, (17): 95-6.	Studies without appropriate outcomes
184	[184] ZHOU YANAN. Clinical Correction Effect of Fluoxetine on Middle School Students with Internet Addiction Accompanied by Depression [J]. 2015, 14(8 %J Today's Health).	Studies were not report their available data
185	[185] BEUKES E W, VLAESCU G, MANCHAIAH V, et al. Development and technical functionality of an Internet-based intervention for tinnitus in the UK [J]. Internet interventions, 2016, 6: 6-15.	Studies were not report their available data
186	[186] CROSBY J M, TWOHIG M P. Acceptance and Commitment Therapy for Problematic Internet Pornography Use: A Randomized Trial [J]. Behavior therapy, 2016, 47(3): 355-66.	Studies were not report their available data
187	[187] LI M, LU L. INTERVENTION STUDY ON THE PSYCHOLOGICAL HEALTH OF CHINESE RURAL LEFT-BEHIND MIDDLE SCHOOL STUDENTS WITH INTERNET ADDICTION DISORDER BY SUNSHINE SPORTS [J]. Revista Argentina De Clinica Psicologica, 2016, 25(2): 167-76.	Studies were not report their available data
188	[188] MORENO M A, ARSENIEV-KOEHLER A, SELKIE E. Development and Testing of a 3-Item Screening Tool for Problematic Internet Use [J]. Journal of Pediatrics, 2016, 176: 167-+.	Studies without appropriate outcomes
189	[189] PARK J H, LEE Y S, SOHN J H, et al. Effectiveness of atomoxetine and methylphenidate for problematic online gaming in adolescents with attention deficit hyperactivity disorder [J]. Human Psychopharmacology-Clinical and Experimental, 2016, 31(6): 427-32.	Studies were not report their available data
190	[190] PARK S-G, PARK J-H, OH CHANGHONG, et al. The Effects of the Forest Environment on Internet Addition Treatment [J]. Journal of Korea Academia-Industrial cooperation Society, 2016,	Studies were not report their available data

	17(4): 489-99.	
191	[191] YUAN K, QIN W, YU D, et al. Core brain networks interactions and cognitive control in internet gaming disorder individuals in late adolescence/early adulthood [J]. Brain Structure & Function, 2016, 221(3): 1427-42.	Studies were not report their available data
192	[192] CHEN BIN. The Impact of Sports Intervention on the State of Internet Addiction in College Students %J Contemporary Sports Science and Technology [J]. 2016, 6(22).	Studies without appropriate outcomes
193	[193] HU GENGDAN, ZHANG JUN. The Role and Mechanism of Sports Correction of Adolescent Internet Addiction from the Perspective of Human Instinct %J China Sports Science [J]. 2016, 52(01): 68-77.	Studies were not report their available data
194	[194] NING KUI, WANG XINKAI, JIN HONG, et al. Observation of the Effects of Cognitive Behavioral Group Psychotherapy on Adolescent Internet Addiction [J]. 2016, (12 %J Journal of Xinxiang Medical University).	Studies without appropriate outcomes
195	[195] WU YAN, YE YINZHEN. Research on the Psychological Intervention of Reading Therapy for College Students with Internet Addiction %J Modern Communication [J]. 2016, (05): 158-9.	Studies were not report their available data
196	[196] ATAŞALAR J. The Effectiveness of a Program Designed to Prevent Problematic Internet Use Among Sixth Graders [M]. 2017.	Studies were not report their available data
197	[197] CALVETE E, GAMEZ-GUADIX M, CORTAZAR N. Mindfulness facets and problematic Internet use: A six-month longitudinal study [J]. Addictive behaviors, 2017, 72: 57-63.	Studies were not report their available data
198	[198] HWA L J, LEE SEUNG HWAN, JO YOUNG HOON, et al. Research Trends in Acupuncture Treatment of Internet Addiction Disorder [J]. The Korean Society of Oriental Neuropsychiatry, 2017, 28(1): 1-13.	Studies without appropriate outcomes
199	[199] KING D L, KAPTSIS D, DELFABBRO P H, et al. Effectiveness of Brief Abstinence for Modifying Problematic Internet Gaming Cognitions and Behaviors [J]. Journal of Clinical Psychology, 2017, 73(12): 1573-85.	Studies were not report their available data
200	[200] LAU J T F, WU A M S, GROSS D L, et al. Is Internet addiction transitory or persistent?	Studies were not report their available data

	Incidence and prospective predictors of remission of Internet addiction among Chinese secondary school students [J]. Addictive behaviors, 2017, 74: 55-62.	
201	[201] PUTRI A P S, POERWANDARI E K, RUSLI E. Social Anxiety and Internet Addiction: CBT Intervention Module Development Based on Self-Reflection; proceedings of the 1st International Conference on Intervention and Applied Psychology (ICIAP), Univ Indonesia, Fac Psychol, Depok, INDONESIA, F 2017 Sep 15-16, 2017 [C]. 2017.	Studies were not report their available data
202	[202] REN Y, YANG J, LIU L. Social Anxiety and Internet Addiction among Rural Left-behind Children: The Mediating Effect of Loneliness [J]. Iranian journal of public health, 2017, 46(12): 1659-68.	Studies without appropriate outcomes
203	[203] T. N C. - The Effectiveness of tDCS on Internet Game Addiction [J]. 2017.	Studies were not report their available data
204	[204] WöLFLING K, MüLLER K W, DREIER M, et al. Short-term treatment of internet and computer game addiction (STICA)-a randomized clinical trial [J]. Journal of behavioral addictions, 2017, 6: 60.	Studies were not report their available data
205	[205] ZHANG M W B, HO R C M. Smartphone applications for immersive virtual reality therapy for internet addiction and internet gaming disorder [J]. Technology and Health Care, 2017, 25(2): 367-72.	Studies were not report their available data
206	[206] CUI XIAOHONG. Prevention and Intervention Countermeasures for Adolescent Internet Addiction from the Perspective of Positive Psychology %J Global Market Information Guide [J]. 2017, 0(37).	Studies without appropriate outcomes
207	[207] LIU JUN. Intervention Study of Exercise Prescription and Psychological Counseling on College Students' Internet Addiction %J Journal of Guangzhou Sport University [J]. 2017, 37(3).	Studies were not report their available data
208	[208] LIU YAPPING. Outdoor Sports Intervention Treatment for College Students' Internet Addiction %J Sports Style [J]. 2017, 0(11).	Studies were not report their available data
209	[209] QIU SHUAI, PENG ZHUOFENG, FENG XUEKE. Group Psychological Counseling Intervention for College Students with Internet Addiction %J Western Quality Education [J]. 2017, 3(13): 116.	Studies without appropriate outcomes
210	[210] KIM S-H, YIM H-W, JO S-J, et al. The Effects of Group Cognitive Behavioral Therapy on the Improvement of Depression and Anxiety in Adolescents with Problematic Internet Use [J]. Journal of	Studies were not report their available data

	the Korean Academy of Child and Adolescent Psychiatry, 2018, 29(2): 73-8.	
211	[211] KUSS D. Treatment of Internet gaming disorder [J]. European Psychiatry, 2018, 48: S62-S3.	Studies without appropriate outcomes
212	[212] LI W, GARLAND E L, HOWARD M O. Therapeutic mechanisms of Mindfulness-Oriented Recovery Enhancement for internet gaming disorder: Reducing craving and addictive behavior by targeting cognitive processes [J]. Journal of addictive diseases, 2018, 37(1-2): 5-13.	Studies were not report their available data
213	[213] NEBTOUN F, NAZARBOLAND N, GHANBARI S. Do brain/behavioral systems mediate the relationship between Internet addiction and executive functions? [J]. European Psychiatry, 2018, 48: S317.	Studies without appropriate outcomes
214	[214] NIELSEN P, RIGTER H. Multidimensional Family Therapy for adolescents with Internet gaming disorder: The design of a transnational treatment study [J]. Journal of behavioral addictions, 2018, 7: 23.	Studies were not report their available data
215	[215] T. K C. - Investigation of treatment efficacy of neuromodulation on internet/game addiction [J]. 2018.	Studies without appropriate outcomes
216	[216] WANG Z, WU L, YUAN K, et al. Cortical thickness and volume abnormalities in Internet gaming disorder: Evidence from comparison of recreational Internet game users [J]. European Journal of Neuroscience, 2018, 48(1): 1654-66.	Studies were not report their available data
217	[217] WöLFLING K, MüLLER K, DREIER M, et al. Efficacy of a cognitive-behavioral intervention on symptoms of internet addiction and associated factors-a closer look at the results from STICA [J]. Journal of behavioral addictions, 2018, 7: 164-5.	Studies were not report their available data
218	[218] GE YING, HUO JUNYU, WANG XINQIANG. Initial Sandplay Characteristics of Adolescents with Internet Addiction %J Chinese Journal of School Health [J]. 2018, 39(03): 383-6.	Studies without appropriate outcomes
219	[219] HUANG DAN. The Effect of Structural Family Therapy on Improving Adolescents with Internet Addiction %J Journal of Jinan Vocational College [J]. 2018, (04): 88-90.	Studies were not report their available data
220	[220] LEI YAN. Research on the Effect of Psychological Counseling in the Treatment of Adolescent Internet Addiction %J Modern Medicine and Health Research [J]. 2018, 2(15): 140+2.	Studies were not report their available data

221	[221] ZHOU HUIYU GUO A. Research on the Intervention of Group Counseling for College Students' Internet Addiction from the Perspective of Positive Psychology %J Journal of Kaifeng Educational Institute [J]. 2018, 38(9).	Studies without appropriate outcomes
222	[222] GHOLAMIAN B, SHAHNAZI H, HASSANZADEH A. The effect of educational intervention based on BASNEF model for reducing internet addiction among female students: a quasi-experimental study [J]. Italian Journal of Pediatrics, 2019, 45(1).	Studies without appropriate outcomes
223	[223] N. I. - Effectiveness of TDCS in treatment of internet addiction in students [J]. 2019.	Studies were not report their available data
224	[224] SZÁSZ-JANOCHA C, VONDERLIN E, LINDENBERG K. A cognitive behavioral group therapy program for adolescents with Internet Gaming and Internet Use Disorder: Effects at 12-months follow-up [J]. Journal of behavioral addictions, 2019, 8: 35.	Studies were not report their available data
225	[225] T. N C. - Efficacy of Transcranial Direct Current Stimulation (tDCS) in Patients Suffering From Pathological Use of Internet Gaming [J]. 2019.	Studies without appropriate outcomes
226	[226] TAN K-A. The Effects of Personal Susceptibility and Social Support on Internet Addiction: an Application of Adler's Theory of Individual Psychology [J]. International journal of mental health and addiction, 2019, 17(4): 806-16.	Studies were not report their available data
227	[227] THROUVALA M A, JANIKIAN M, GRIFFITHS M D, et al. The role of family and personality traits in Internet gaming disorder: A mediation model combining cognitive and attachment perspectives [J]. Journal of behavioral addictions, 2019, 8(1): 48-62.	Studies were not report their available data
228	[228] ZHANG S, SONG Y. Effects of different motor skills intervention on college students' internet addiction [J]. Basic and Clinical Pharmacology and Toxicology, 2019, 124: 210-1.	Studies without appropriate outcomes
229	[229] JI ZIYANG, LI YAN, JIN HONG, et al. Analysis of the Effects of Cognitive Behavioral Group Psychotherapy on Adolescents with Internet Addiction [Z]. Proceedings of the 21st National Academic Conference of the Chinese Medical Association. Chongqing. 2019: 80-3	Studies were not report their available data
230	[230] NIE HUI. Research on the Intervention of Positive Psychology for Mobile Internet Addiction among College Students %J Journal of Qiqihaer University: Philosophy and Social Science Edition [J]. 2019, (4).	Studies without appropriate outcomes

231	[231] YIN XIAYUN, YU BINYANG, ZHAO WENYE. Internet Gaming Addiction and Its Intervention in Adolescents: The Perspective of Family Risk %J Contemporary Education and Practice [J]. 2019, 11(05): 17-23.	Studies were not report their available data
232	[232] GANSNER M, NISENSON M, CARSON N, et al. A pilot study using ecological momentary assessment via smartphone application to identify adolescent problematic internet use [J]. Psychiatry Research, 2020, 293.	Studies were not report their available data
233	[233] HAN J, SEO Y, HWANG H, et al. - Efficacy of cognitive behavioural therapy for internet gaming disorder [J]. 2020, - 27(- 2).	Studies were not report their available data
234	[234] LUKAVSKA K, VACEK J, GABHELIK R. The effects of parental control and warmth on problematic internet use in adolescents: A prospective cohort study [J]. Journal of behavioral addictions, 2020, 9(3): 664-75.	Studies were not report their available data
235	[235] N. I. - The effect of intervention of internet gaming disorder among adolescents [J]. 2020.	Studies were not report their available data
236	[236] PARK M, JUNG M H, LEE J, et al. Neurophysiological and Cognitive Correlates of Error Processing Deficits in Internet Gaming Disorder [J]. Cerebral Cortex, 2020, 30(9): 4914-21.	Studies were not report their available data
237	[237] SARI S, KARAGUN E. The Effect of Sports on Children's Internet Addiction, Optimism and Communication Skills [J]. International Journal of Applied Exercise Physiology, 2020, 9(11): 157-66.	Studies without appropriate outcomes
238	[238] SARUHANJAN K, ZARSKI A-C, SCHAUB M P, et al. Design of a Guided Internet- and Mobile-Based Intervention for Internet Use Disorder-Study Protocol for a Two-Armed Randomized Controlled Trial [J]. Frontiers in psychiatry, 2020, 11.	Studies were not report their available data
239	[239] SZASZ-JANOCHA C, VONDERLIN E, LINDENBERG K. Effectiveness of an early intervention program for adolescents with Internet Gaming and Internet Use Disorder: Medium-term effects of the PROTECT plus Study [J]. Zeitschrift Fur Kinder-Und Jugendpsychiatrie Und Psychotherapie, 2020, 48(1): 3-14.	Studies were not report their available data
240	[240] T. N C. - A Cognitive Behavioral Therapy Intervention for Internet Gaming Disorder [J]. 2020.	Studies were not report their available data
241	[241] WU L-L, POTENZA M N, ZHOU N, et al. A role for the right dorsolateral prefrontal cortex in enhancing regulation of both craving and negative emotions in internet gaming disorder: A randomized	Studies without appropriate outcomes

	trial [J]. European Neuropsychopharmacology, 2020, 36: 29-37.	
242	[242] ZHANG X, SHI X, XU S, et al. The Effect of Solution-Focused Group Counseling Intervention on College Students' Internet Addiction: A Pilot Study [J]. International journal of environmental research and public health, 2020, 17(7).	Studies were not report their available data
243	[243] ZHANG Y-Y, CHEN J-J, YE H, et al. Psychological effects of cognitive behavioral therapy on internet addiction in adolescents A systematic review protocol [J]. Medicine, 2020, 99(4).	Studies were not report their available data
244	[244] ZHOU B, ZHANG W, LI Y, et al. Motivational but not executive dysfunction in attention deficit/hyperactivity disorder predicts internet addiction: Evidence from a longitudinal study [J]. Psychiatry Research, 2020.	Studies without appropriate outcomes
245	[245] GE MINGHUI. Group Positive Psychotherapy Intervention for Junior High School Students with Internet Addiction [D]; Jiangxi University of Finance and Economics, 2020.	Studies were not report their available data
246	[246] JI ZIYANG, LI YAN, GU JINGYANG, et al. The Effects of Cognitive Behavioral Therapy-Oriented Group Psychotherapy on Coping Styles, Time Management, and Family Functioning in Middle School Students with Internet Addiction %J Chinese Journal of Behavioral Medicine and Brain Science [J]. 2020, 29(2): 136-41.	Studies without appropriate outcomes
247	[247] LI PEIPEI, XU FANGFANG, YANG HENG. Research on College Students' Internet Addiction Based on Sandplay Therapy %J Chinese Journal of Multimedia and Network Teaching (Mid-month Edition) [J]. 2020, (11): 202-4.	Studies were not report their available data
248	[248] LONG SULAN, YU DAOFANG, LONG FENGYUN, et al. Survey and Intervention Strategy Research on Internet Addiction among Medical College Students [J]. Comparative Research in Culture and Innovation [J]. 2020, 4(07): 64-5.	Studies were not report their available data
249	[249] OUYANG YE. Intervention Analysis and Application of Existential Group Therapy for Adolescent Internet Addiction [J]. Journal of Jincheng Vocational and Technical College [J]. 2020, 13(03): 64-6+93.	Studies without appropriate outcomes

250	[250] YU GUIXIANG. Analysis of Nursing Model and Implementation Effect for Inpatient Internet Addiction Adolescents %J Journal of General Stomatology [J]. 2020, 7(02): 86+100.	Studies were not report their available data
251	[251] ZHANG QINGFENG. Empirical Research on the Intervention of Physical Exercise for Middle School Students' Internet Addiction %J Bulletin of Sports Science and Technology [J]. 2020, 28(9).	Studies without appropriate outcomes
252	[252] ZHAO JING, FENG HAIJIAO, CHEN CHANGXIANG. The Current Situation of Mobile Internet Addiction and Influencing Factors among College Students in Universities Far from Central Cities [J]. 2020, 37(2 %J Chinese Journal of Health Psychology).	Studies were not report their available data
253	[253] AFFOUNEH S, MAHAMID F A, BERTE D Z, et al. The efficacy of a training program for social skills in reducing addictive Internet behaviors among Palestinian university students [J]. Psicologia-Reflexao E Critica, 2021, 34(1).	Studies were not report their available data
254	[254] CAI H, XI H T, ZHU Q, et al. Prevalence of problematic Internet use and its association with quality of life among undergraduate nursing students in the later stage of COVID-19 pandemic era in China [J]. American Journal on Addictions, 2021, 30(6): 585-92.	Studies were not report their available data
255	[255] CHEN Y, ZHANG L, LIU Y, et al. Acupuncture for Internet addiction A protocol for systematic review [J]. Medicine, 2021, 100(12).	Studies were not report their available data
256	[256] CHICTR. - Neural mechanism of electroacupuncture in alleviating internet craving in PIU [J]. 2021.	Studies without appropriate outcomes
257	[257] CHICTR. - A randomized controlled trial of the efficacy of Internet-based cognitive behavioral therapy on subthreshold depression in Chinese adults [J]. 2021.	Studies were not report their available data
258	[258] DIERIS-HIRCHE J, BOTTEL L, PAPE M, et al. Effects of an online-based motivational intervention to reduce problematic internet use and promote treatment motivation in internet gaming disorder and internet use disorder (OMPRIS): Study protocol for a randomised controlled trial [J]. BMJ open, 2021, 11(8).	Studies were not report their available data
259	[259] HE J, PAN T, NIE Y, et al. Behavioral modification decreases approach bias in young adults with internet gaming disorder [J]. Addictive behaviors, 2021, 113: 106686.	Studies were not report their available data

260	[260] SARKAR S, BHANDARY S, ARYA A. Effectuating Supervised Machine Learning Techniques for Multiclass Classification of Problematic Internet and Mobile Usage; proceedings of the International Conference on Computing, Communication, and Intelligent Systems (ICCCIS), Noida, INDIA, F 2021 Feb 19-20, 2021 [C]. 2021.	Studies without appropriate outcomes
261	[261] SUN X, DUAN C, NIU G, et al. Mindfulness buffers the influence of stress on cue-induced craving for Internet among Chinese colleges with problematic Internet use [J]. Journal of behavioral addictions, 2021, 10(4): 983-9.	Studies were not report their available data
262	[262] SZÁSZ-JANOCHA C, VONDERLIN E, LINDENBERG K. Treatment outcomes of a CBT-based group intervention for adolescents with Internet use disorders [J]. Journal of behavioral addictions, 2021, 9(4): 978-89.	Studies were not report their available data
263	[263] T. N C. - Examining the Efficacy of "Parental Vigilant Care" for Reducing Problematic Internet Usage [J]. 2021.	Studies were not report their available data
264	[264] TCTR. - Effectiveness of the program based on cognitive behavioral therapy compared to the program based on Satir model therapy among child and adolescent with online game addiction [J]. 2021.	Studies were not report their available data
265	[265] WU L L, POTENZA M N, ZHOU N, et al. Efficacy of single-session transcranial direct current stimulation on addiction-related inhibitory control and craving: a randomized trial in males with Internet gaming disorder [J]. Journal of psychiatry & neuroscience : JPN, 2021, 46(1): E111-e8.	Studies were not report their available data
266	[266] CHEN JIEYU, XIANG HUI. Mindfulness Intervention and Its Possible Mechanisms for Internet Gaming Addiction %J Journal of Clinical Psychiatry [J]. 2021, 31(01): 82-4.	Studies were not report their available data
267	[267] CHEN QING, LIAO YAN. The Application of Group Psychotherapy in Adolescent Internet Addiction [J]. 2021, 15(11 %J China Modern Medical Application).	Studies were not report their available data
268	[268] HUANG CHANGRONG. Effect Observation of Mindfulness Training Combined with Agomelatine in the Treatment of Patients with Internet Addiction and Depression [J]. 2021, 25(3 %J Practical Clinical Medical Magazine).	Studies were not report their available data

269	[269] JIANG ZHOUBIN ZHENG A. Research on the Intervention of Physical Exercise for College Students' Internet Addiction %J Contemporary Sports Science and Technology [J]. 2021, 11(14).	Studies were not report their available data
270	[270] QI ENXIU, YAO HAIQIN, WU QIUFENG. Analysis of the Effect of Psychological Nursing Intervention on Inpatient Internet Addiction Adolescents %J Psychological Monthly [J]. 2021, 16(23): 181-3.	Studies were not report their available data
271	[271] TANG SHIJIE, TAO GUANGYUAN, LIU CHANJUAN, et al. Clinical Correction Effect of Fluoxetine on Middle School Students with Internet Addiction Accompanied by Depression %J Health Friend [J]. 2021, (2): 289.	Studies were not report their available data
272	[272] ZHAO YUMENG. A Case Study on the Positive Psychological Intervention for Middle School Students' Internet Dependence [D]; Shanxi University, 2021.	Studies were not report their available data
273	[273] ACTRN. - Parent education and behavioural management interventions for Screens and Gaming Disorder in children [J]. 2022.	Studies were not report their available data
274	[274] BISCHOF A, BRANDT D, SCHLOSSAREK S, et al. Study protocol for a randomised controlled trial of an e-health stepped care approach for the treatment of internet use disorders versus a placebo condition: the SCAPIT study [J]. BMJ open, 2022, 12(11).	Studies were not report their available data
275	[275] CHANG C-H, CHANG Y-C, YANG L, et al. The Comparative Efficacy of Treatments for Children and Young Adults with Internet Addiction/Internet Gaming Disorder: An Updated Meta-Analysis [J]. International journal of environmental research and public health, 2022, 19(5).	Studies were not report their available data
276	[276] CTRI. - Effect of yoga module for the management of internet gaming among children [J]. 2022.	Studies were not report their available data
277	[277] LINDENBERG K, HOFMANN S. Examining the effects of dissonance-based primary prevention of Internet and computer-game addiction on attitudes toward gaming among grade 5 pupils: A pilot study [J]. Zeitschrift Fur Kinder-Und Jugendpsychiatrie Und Psychotherapie, 2022, 50(1): 25-36.	Studies were not report their available data
278	[278] MANNIKKO N, MUSTONEN T, TANNER N, et al. Effectiveness of a Brief Group Intervention Program for Young Adults with Gaming-Related Problems [J]. International journal of mental health and addiction, 2022, 20(5): 2956-72.	Studies were not report their available data

279	[279] N. I. - The Effect of Education Based on Self-Management Empowerment Model on the Sense of Coherence, Coping Strategies and Self-Efficacy of Girl Adolescents with Internet Addiction [J]. 2022.	Studies were not report their available data
280	[280] O'CONNOR K A, BAGNELL A, ROSYCHUK R J, et al. A randomized controlled trial evaluating the effect of an internet-based cognitive-behavioral program on anxiety symptoms in a community-based sample of adolescents [J]. Journal of Anxiety Disorders, 2022, 92.	Studies were not report their available data
281	[281] PAKPOUR A H, FAZELI S, ZEIDI I M, et al. Effectiveness of a mobile app-based educational intervention to treat internet gaming disorder among Iranian adolescents: study protocol for a randomized controlled trial [J]. Trials, 2022, 23(1).	Studies were not report their available data
282	[282] RIGTER H, NIELSEN P. Family therapy decreases Internet Gaming Disorder in adolescents [J]. Journal of behavioral addictions, 2022, 11: 178-9.	Studies were not report their available data
283	[283] SCHMIDT H, BRANDT D, MEYER C, et al. Motivational brief interventions for adolescents and young adults with Internet use disorders: A randomized-controlled trial [J]. Journal of behavioral addictions, 2022, 11(3): 754-65.	Studies were not report their available data
284	[284] SCHMIDT H, BRANDT D, MEYER C, et al. Brief telephone-based intervention for adolescents and young adults with problematic Internet use: Results from the randomized-controlled iPIN study [J]. Journal of behavioral addictions, 2022, 11: 52.	Studies were not report their available data
285	[285] SHARMA M K, ANAND N, TADPATRIKAR A, et al. Effectiveness of multimodal psychotherapeutic intervention for internet gaming disorder [J]. Psychiatry Research, 2022, 314.	Studies were not report their available data
286	[286] SISTE K, HANAFI E, ADRIAN L T, et al. Online dialectical behavioral therapy for adults with internet addiction: A quasi-experimental trial during the COVID-19 pandemic [J]. Psychiatry Research, 2022, 315.	Studies were not report their available data
287	[287] ZHANG W, XU R. Effect of Exercise Intervention on Internet Addiction and Autonomic Nervous Function in College Students [J]. BioMed research international, 2022.	Studies were not report their available data
288	[288] ZHAO Q, ZHANG Y, WANG M, et al. Effects of retrieval-extinction training on internet gaming disorder [J]. Journal of behavioral addictions, 2022, 11(1): 49-62.	Studies were not report their available data

289	[289] LIU YANG MA A XIA A LI A. Mechanism, Impact and Strategy Study on the Intervention of Sports Activities for Adolescent Internet Addiction %J Journal of Shenyang Sport Institute [J]. 2022, 41(6).	Studies were not report their available data
290	[290] REN JUAN, ZHANG JIANGHUI, ZHANG XUEQING, et al. The Relationship between Adolescent Internet Addiction and Sleep Disorders: A Moderated Mediation Effect Model [J]. 2022, 30(9 %J Chinese Journal of Health Psychology).	Studies were not report their available data
291	[291] MUELLER K W, DREIER M, BEUTEL M E, et al. The impact of life satisfaction in the treatment of gaming disorder and other internet use disorders: Results from a randomized controlled trial [J]. Journal of behavioral addictions, 2023, 12(1): 159-67.	Studies were not report their available data
292	[292] MUN I B, LEE S. The influence of parents' depression on children's online gaming addiction: testing the mediating effects of intrusive parenting and social motivation on children's online gaming behavior [J]. Current Psychology, 2023, 42(6): 4991-5000.	Studies were not report their available data
293	[293] PRASETIAWAN H, BARIDA M, HANDAKA I B, et al. Counselor Strategies to Reduce Online Gaming Addiction through Creative Cognitive-Behavior Group Counseling [J]. Pegem Egitim Ve Ogretim Dergisi, 2023, 13(2): 147-52.	Studies were not report their available data
294	[294] PU Y, LIU Y, QI Y, et al. Five-week of solution-focused group counseling successfully reduces internet addiction among college students: A pilot study [J]. Journal of behavioral addictions, 2023, 12(4): 964-71.	Studies were not report their available data
295	[295] T. K C. - Effectiveness of Cognitive-Behavior Therapy for Adolescents with High-risk of Internet Gaming Disorder [J]. 2023.	Studies were not report their available data
296	[296] T. N C. - WeChat-based Intervention for Internet Addiction [J]. 2023.	Studies were not report their available data
297	[297] T. N C. - Effect of Distress Tolerance Training on Problematic Internet Use and Psychological Wellbeing Among Faculty Nursing Students [J]. 2023.	Studies were not report their available data
298	[298] TAGHIPOUR E, VIZESHFAR F, ZARIFSANAIEY N. The effect of gamification-based training on the knowledge, attitudes, and academic achievement of male adolescents in preventing substance and internet addiction [J]. BMC medical education, 2023, 23(1): 860.	Studies were not report their available data

299	[299] ZHANG Y, ZHONG Y-L, LUO J, et al. Effects of resourcefulness on internet game addiction among college students: The mediating role of anxiety and the moderating role of gender [J]. Frontiers in public health, 2023, 11.	Studies were not report their available data
300	[300] ZHENG HONGBO WEI A ZENG A WANG A LI A. The Impact of Mindfulness Training Combined with EEG Biofeedback Treatment on Adolescents with Depression and Internet Addiction %J Chinese Journal of General Practice [J]. 2023, 21(2).	Studies without appropriate outcomes
301	[301] ISRCTN. - Cognitive training as treatment for games or other types of internet addiction [J]. 2021.	Studies were not report their available data
