# Supplementary material for: Optimal Non-Pharmacological Interventions for Reducing Problematic Internet Use in Youth: A Systematic Review and Bayesian Network Meta-Analysis
Source: Behav Sci (Basel). 2025 Jan 20;15(1):98. doi: 10.3390/bs15010098 (PMC11762159; doi:10.3390/bs15010098)
Supplement: Supplementary file 1 [file behavsci-15-00098-s001.zip › Supplement Appendix S4. The list of included studies.pdf]

The list of included studies

[1] BAI Y, FAN F. The Effects of Group Counseling on Internet Dependent College Students [J]. Chinese Mental Health Journal, 2007, (04): 247-50.

[2] ZHENG W, YE M, LIN F, et al. Effect Analysis of Group Psychotherapy in College Students with Internet Addiction [J]. Medicine and Society, 2007, 20(6): 28-30.

[3] CAO FENGLIN, SU LINYAN, GAO XUEPING, et al. Controlled Study on Group Psychotherapy for Excessive Internet Use in Middle School Students [J]. Chinese Mental Health Journal, 2007, (05): 346-9+58.

[4] GUO M, YU F, CHAO X, et al. Impact Evaluation of Group Counseling on Internet Addiction Adolescents [J]. Chinese Journal of School Health, 2008, 29(1): 17-9.

[5] KIM J-U. The effect of a R/T group counseling program on the Internet addiction level and self-esteem of Internet addiction university students [J]. International Journal of Reality Therapy, 2008, 27(2).

[6] ZHU TIANGE, JIN RONGJIANG, ZHONG XIAOMING, et al. The Impact of Electroacupuncture Combined with Psychological Intervention on Anxiety Status and Serum NE Changes in Internet Addiction Patients [J]. Chinese Acupuncture & Moxibustion, 2008, 28(8).

[7] LIAO X. Intervention of Aerobic Exercise for Internet Addiction College Students [J]. Journal of Hunan University of Technology, 2009, 23(6).

[8] ZHANG W, LU J. Research on Group Counseling of Injecting Emotional Factors into Internet Addicted College Students [J]. Psychological Science, 2009, 32(03): 525-7.

[9] LI GENG. Cognitive Behavioral Therapy for Adolescent Internet Addiction [D]. Ningxia Medical University, 2009.

[10] LI GENG, DAI XIUYING. Controlled Study on Cognitive Behavioral Therapy for Adolescent Internet Addiction [J]. Chinese Mental Health Journal, 2009, 23(7).

[11] ZHANG LANJUN. Intervention of Group Psychotherapy and Sports Prescription on College Students' Internet Addiction [J]. Psychological Science, 2009, 32(03): 738-41.

[12] ZHU TIANGE, JIN RONGJIANG, ZHONG XIAOMING, et al. Clinical Study on the Treatment of Internet Addiction Syndrome with Electroacupuncture Combined with Psychological Intervention [J]. Chinese Journal of Integrated Traditional and Western Medicine, 2009, 29(3): 212-4.

[13] CHEN K, ZHENG W, MU S. Evaluation on the Effect of Group Psychotherapy for Internet Addiction Students in Wenzhou Universities [J]. Chinese Journal of School Health, 2010, 31(06): 665-6.

[14] DU Y S, JIANG W, VANCE A. Longer Term Effect of Randomized, Controlled Group Cognitive Behavioral Therapy for Internet Addiction in Adolescent Students in Shanghai [J]. Australian and New Zealand Journal of Psychiatry, 2010, 44(2): 129-34.

[15] HUANG Z, QIAN M, ZHU S, et al. Effects of Interpersonal Group Counseling on College Students with Computer Gaming Addiction [J]. Chinese Mental Health Journal, 2010, 24(01): 29-33.

[16] LIU W, WEN B, YAO G, et al. Efficacy of Centralized and Closed Group Counseling on Internet-Dependent Undergraduates [J]. Chinese Journal of School Health, 2010, 31(04): 390-1+4.

[17] YE HUA. The Impact of Electroacupuncture Combined with Psychological Rehabilitation Treatment on Clinical Symptoms and Cognitive Potentials in IAD Patients [D]. Chengdu

University of Traditional Chinese Medicine, 2010.
[18] CHEN Z, TANG Y, ZHANG X, et al. Evaluation of Intervention Effect of Staged Group Counseling on Internet Addicted College Students [J]. Chinese Journal of School Health, 2011, 32(06): 726-8.
[19] PARK G R, KIM H S. Effects of a Group Counseling Integration Program on Self-determination and Internet Addiction in High School Students with Tendency to Internet Addiction [J]. Journal of Korean Academy of Nursing, 2011, 41(5): 694-703.
[20] QIU Q. Experimental Study of E-sports' Effects on Addictive Behaviors [J]. Journal of Shenyang Sport University, 2011, 30(2).
[21] ZHONG X, ZU S, SHA S, et al. THE EFFECT OF A FAMILY-BASED INTERVENTION MODEL ON INTERNET-ADDICTED CHINESE ADOLESCENTS [J]. Social Behavior and Personality, 2011, 39(8): 1021-34.
[22] KONG MING, FU WENQING, LIU WEI, et al. Research on the Impact of Group Psychological Counseling on College Students' Internet Addiction [J]. Chinese Journal of Clinical Psychology, 2011, 19(1).
[23] WANG JIE, ZHANG A. Research on the Improvement of Psychological Control Source and Physical and Mental Health of Vocational College Students with Internet Addiction through Group Psychological Counseling [J]. Chinese School Doctor, 2011, 25(2).
[24] WANG YINGLI, DAI XIUYING, LI QIULI, et al. Follow-up Clinical Efficacy of Cognitive Behavioral Therapy for Adolescent Internet Addiction [J]. Chinese Journal of Behavioral Medicine and Brain Science, 2011, 20(3).
[25] ZHANG ZHUOYING. Empirical Study on Psychological Intervention of College Students' Internet Addiction [D]. Sichuan Agricultural University, 2011.
[26] ZHU TIANGE, LI HUI, DU YUPENG, et al. The Impact of Electroacupuncture Combined with Psychological Intervention on the Brain's Working Ability of Internet Addiction Patients [J]. Chinese Journal of Traditional Medicine, 2011, 52(19).
[27] ZHU TIANGE, LI HUI, DU YUPENG, et al. The Impact of Electroacupuncture Combined with Psychological Intervention on Internet Addiction Patients' Craving and Brain Electroencephalogram Ultra-Slow Fluctuation Map: A Randomized Controlled Study [J]. Chinese Acupuncture & Moxibustion, 2011, 31(5).
[28] DUAN S, YE M. "Psychological Intervention in College Students' Internet Dependence and its Effect: Based on the Control Test in Hunan Police Academy" [J]. Journal of Hunan Agricultural University (Social Sciences), 2012, 13(06): 63-7.
[29] LV W, ZHANG B. The Effects of Group Counseling on Internet-Dependent College Students [J]. China Journal of Health Psychology, 2012, 20(12): 1845-7.
[30] WANG Q. Evaluation of Intervention Effect of Internet Addiction by Cognitive Behavioral Therapy for College Students [J]. Journal of Huangshi Institute of Technology, 2012, 28(4).
[31] ZHU T-M, LI H, JIN R-J, et al. Effects of Electroacupuncture Combined Psycho-intervention on Cognitive Function and Event-Related Potentials P300 and Mismatch Negativity in Patients with Internet Addiction [J]. CHINESE JOURNAL OF INTEGRATIVE MEDICINE, 2012, 18(2): 146-51.
[32] GUAN MINGYUE, QIAN SHAOYUE, LIU WEI. Effectiveness Study on Group Therapy for Vocational College Students' Internet Dependence [J]. Chinese General Practice, 2012, 15(34): 4000-2.

[33] LI YINGCUN. Current Status and Psychological Intervention Controlled Study on Internet Addiction in First-Year Middle School Students in Jinan [D]. Shandong University, 2012.
[34] XIAO KUNPENG, GAO A, SUN A. Empirical Study on the Impact of Sports Intervention on College Students' Internet Addiction [J]. Journal of Shenyang Sport University, 2012, 31(4).
[35] XU WEI, LIU ZHIYING. Study on the Intervention of Self-Control Based Group Counseling for Middle School Students' Internet Addiction [J]. Journal of Shijiazhuang University, 2012, 14(6): 92-5.
[36] LIU DING, LU NING, HE JIANFEI, et al. The Effect of Group Counseling on Improving Internet Usage and Study Management in College Students with Internet Addiction [J]. Chinese Mental Health Journal, 2013, 27(7).
[37] LU YANHUI, YANG YONGXIN, MU JIANGBING, et al. The Impact of the Satir Family Therapy Model on Family Intimacy and Adaptability in Patients with Internet Addiction [J]. Chinese Journal of Behavioral Medicine and Brain Science, 2013, 22(5): 425-7.
[38] YANG YANG. Study on the Impact of Electroacupuncture Therapy on Impulsive Behavior and Brain Magnetic Resonance Spectroscopy (MRS) in Internet Addiction Patients [D]. Chengdu University of Traditional Chinese Medicine, 2013.
[39] ZHANG CHENGSHENG. Study on the Intervention of Sports Activities for College Students' Internet Addiction in Kunming [J]. Sports Science, 2013, 34(4).
[40] GE Y, HU Y, ZHANG Z, et al. Effects of Psychodrama Group Counseling on Internet Addiction and Social Avoidance in Urban Left-Behind Children [J]. Chinese Mental Health Journal, 2014, 28(6): 458-65.
[41] LIU H. Research on Group Intervention of Network Dependence of Primary and Middle School Students [J]. FUJIAN INSTITUTE OF EDUCATION, 2014, 15(02): 10-5+91+128.
[42] DU YUFENG, LI XIAOMIN, HE XUEJUAN, et al. The Intervention Effect of the Satir Family Therapy Model on Adolescent Internet Addiction [J]. Journal of Chengde Medical College, 2014, 31(05): 459-60.
[43] MING LIJUAN. Evaluation Study on the Intervention Effect of Behavioral Cognitive Therapy for Medical Students' Internet Addiction [D]. Shandong University, 2014.
[44] WEI JIAWEI, REN A, AO A, HE A, LI A. Experimental Study on the Intervention of Sports Activities for College Students' Internet Addiction [J]. Heilongjiang Medical Science, 2014, 37(4).
[45] WEI QIUXIANG, YANG YONGXIN, WANG CHUNMEI, et al. Discussion on the Intervention Effect of the Whole Course Synchronous Intervention for Patients with Internet Addiction and Their Parents [J]. Nursing Journal, 2014, 29(19).
[46] LIU Q X, FANG X Y, YAN N, et al. Multi-family Group Therapy for Adolescent Internet Addiction: Exploring the Underlying Mechanisms [J]. Addictive Behaviors, 2015, 42: 1-8.
[47] ZHAO H, HU X. An Empirical Study of College Students' Internet Addiction on Group Counseling [J]. Ideological and Political Education Research, 2015, 31(01): 138-40.
[48] LI XIN. Analysis of the Intervention Effect of Group Counseling on Different Degrees of Internet Dependence in Vocational Medical Students [J]. Chinese School Health, 2015, 36(8).
[49] PENG WANXIU, LI ZHONGGUO. The Impact of Group Psychotherapy on Internet Addiction and Mental Health in Middle School Students [J]. Clinical Psychosomatic Diseases, 2015, 21(4).
[50] ZHANG JIANGXING, WANG PEIPEI. Empirical Comparison of Typical Psychotherapeutic Methods for College Students' Internet Addiction [J]. Psychological Exploration, 2015, 35(06):

557-60.
[51] ÇELIK Ç B. Educational Intervention for Reducing Internet Addiction Tendencies [J]. The Turkish Journal on Addictions, 2016, 3(3): 375-86.
[52] PARK S Y, KIM S M, ROH S, et al. The Effects of a Virtual Reality Treatment Program for Online Gaming Addiction [J]. Computer Methods and Programs in Biomedicine, 2016, 129: 99-108.
[53] ZHAO SONGTAO, YANG YONGXIN, WEI QIUXIANG, et al. Efficacy of Group Cognitive Behavioral Therapy for Adolescent Internet Addiction Syndrome [J]. Armed Police Medical Journal, 2016, (4).
[54] DENG L-Y, LIU L, XIA C-C, et al. Craving Behavior Intervention in Ameliorating College Students' Internet Game Disorder: A Longitudinal Study [J]. 2017, 8: 526.
[55] KHAZAEI F, KHAZAEI O, GHANBARI-H B. Positive Psychology Interventions for Internet Addiction Treatment [J]. Computers in Human Behavior, 2017, 72: 304-11.
[56] LI L, HUANG Y. A Study on the Intervention of Cognitive Behavior Group Counseling on Poor College Students with Internet Addiction [J]. Modern Education Science, 2017, (04): 117-21.
[57] LI W, GARLAND E L, MCGOVERN P, et al. Mindfulness-Oriented Recovery Enhancement for Internet Gaming Disorder in US Adults: A Stage I Randomized Controlled Trial [J]. Psychology of Addictive Behaviors, 2017, 31(4): 393-402.
[58] YANG C-Y, ZENG G-F. The Effect of Tai Chi Exercise on College Students' Internet Addiction [J]. Chinese Journal of School Health, 2017, 38(02): 292-4.
[59] YANG Y, LI H, CHEN X-X, et al. Electro-acupuncture Treatment for Internet Addiction: Evidence of Normalization of Impulse Control Disorder in Adolescents [J]. Chinese Journal of Integrative Medicine, 2017, 23(11): 837-44.
[60] CHENG GENGJINSHENG, LUO JIANGHONG. The Intervention Effect of EEG Biofeedback on Adolescent Internet Addiction [J]. Chinese School Health, 2017, 38(11).
[61] XU JIABING, TANG A, LIU A, ZHOU A, YANG A, YANG A. Clinical Efficacy of Cognitive Behavioral Therapy Combined with Electroacupuncture in Treating Online Game Addiction [J]. Clinical Medical Research and Practice, 2017, 2(35).
[62] CHEN S, WU S. Effect of Group Cognitive Behavioral Therapy on Adolescent Internet Addiction Patients [J]. Chinese and Foreign Medical Research, 2018, 16(15): 172-3.
[63] CHEN SUZHEN, WU SUYING. Effect Analysis of Group Cognitive Behavioral Therapy on Adolescent Internet Addiction Patients [J]. Chinese and Foreign Medical Research, 2018, 16(15): 172-3.
[64] TANG RENZHIHUI, LIU XUEJUN, YANG XULI, et al. Analysis of the Impact of Electroacupuncture Combined with Cognitive Behavioral Therapy on the Quality of Life of Online Game Addicts [J]. Harbin Medical Journal, 2018, 38(2).
[65] XU ZHIXIAO, XU HUABING. Clinical Efficacy of Group Psychotherapy for Internet Addiction Youth [J]. Journal of Clinical Rational Drug Use, 2018, 11(36).
[66] ZHANG LIBIN, WANG YAJING, LI GANG, et al. Intervention Study of Family Sandplay Therapy on Adolescent Internet Addiction [J]. Journal of Guangdong Medical College, 2018, 36(3).
[67] FANG H, DAI Y. Psychological Intervention of Positive Psychology on Internet Addiction of College Students [J]. China Journal of Health Psychology, 2019, 27(12): 1906-10.
[68] WOELFLING K, MUELLER K W, DREIER M, et al. Efficacy of Short-term Treatment of Internet and Computer Game Addiction A Randomized Clinical Trial [J]. JAMA Psychiatry, 2019, 76(10): 1018-25.

[69] HONG J S, KIM S M, KANG K D, et al. Effect of Physical Exercise Intervention on Mood and Frontal Alpha Asymmetry in Internet Gaming Disorder: Physical Exercise Intervention for IGD [J]. Mental Health and Physical Activity, 2020, 18.
[70] MATHEW P, KRISHNAN R, BHASKAR A. Effectiveness of a Nurse-Led Intervention for Adolescents With Problematic Internet Use [J]. Journal of Psychosocial Nursing and Mental Health Services, 2020, 58(7): 16-26.
[71] ZHONG Y-H. Effect of rTMS on Decreasing Internet Addicts' Craving for Internet [J]. Journal of Gannan Medical University, 2020, 40(07): 727-9.
[72] HUANG LEI. Mindfulness Intervention Study on High School Students' Internet Addiction [D]. Chongqing Normal University, 2020.
[73] SONG GUOYING, MAO DONGXUE, WU QIAN, et al. Study on the Intervention Effect of Positive Psychology Group Counseling on Internet Addiction Medical Students of "Post-2000s" [J]. Health Vocational Education, 2020, 38(15): 154-7.
[74] ALAVI S S, GHANIZADEH M, MOHAMMADI M R, et al. The Effects of Cognitive-Behavioral Group Therapy for Reducing Symptoms of Internet Addiction Disorder and Promoting Quality of Life and Mental Health [J]. Trends in Psychiatry and Psychotherapy, 2021, 43(1): 47-56.
[75] BONG S H, WON G H, CHOI T Y. Effects of Cognitive-Behavioral Therapy Based Music Therapy in Korean Adolescents with Smartphone and Internet Addiction [J]. 2021, 18(2): 110.
[76] DICLE A N. An Educational Approach for Decreasing Internet Addiction: Motivational Interviewing Psycho-Education Program [J]. Eurasian Journal of Educational Research, 2021, (94): 25-48.
[77] JEONG H, OH J K, CHOI E K, et al. Effects of Transcranial Direct Current Stimulation on Addictive Behavior and Brain Glucose Metabolism in Problematic Online Gamers [J]. Journal of Behavioral Addictions, 2021, 9(4): 1011-21.
[78] LEE J Y, JANG J H, CHOI A R, et al. Neuromodulatory Effect of Transcranial Direct Current Stimulation on Resting-State EEG Activity in Internet Gaming Disorder: A Randomized, Double-Blind, Sham-Controlled Parallel Group Trial [J]. Cerebral Cortex Communications, 2021, 2(1).
[79] LIU X, JIANG J, ZHANG Y. Effects of Logotherapy-Based Mindfulness Intervention on Internet Addiction among Adolescents during the COVID-19 Pandemic [J]. Iranian Journal of Public Health, 2021, 50(4): 789-97.
[80] PENG W, WANG Y, HAO Q, et al. Effects of Electroacupuncture Combined With Psychological Intervention on Depressive Status and Contingent Negative Variation in Patients With Internet Addiction Disorder: A Randomized Controlled Trial [J]. Frontiers in Psychiatry, 2021, 12.
[81] LU ZHAOJING, LI ZHIMING, YAN WANSEN. The Intervention Effect of Group Cognitive Therapy on Negative Emotions and Impulsivity in College Students with Internet Addiction [J]. Chinese Journal of School Health, 2021, 42(6).
[82] YANG DONG. Study on the Intervention Effect of Different Exercise Methods on College Students' Internet Addiction [J]. Journal of Pingdingshan University, 2021, 36(5): 124-8.
[83] YIN YANLING. Investigation and Intervention Study on the Current Status of Internet Addiction in Rural Junior Middle School Students [D]. Chongqing Normal University, 2021.
[84] AGBARIA Q. Cognitive Behavioral Intervention in Dealing with Internet Addiction among Arab Teenagers in Israel [J]. International Journal of Mental Health and Addiction, 2022.
[85] GONG Y, GONG N, ZHANG Z, et al. Effect of Narrative Therapy-Based Group Psychological Counseling on Internet Addiction among Adolescents [J]. Iranian Journal of Public Health, 2022, 51(6):

1331-8.
[86] LINDENBERG K, KINDT S, SZASZ-JANOCHA C. Effectiveness of Cognitive Behavioral Therapy to Prevent Gaming Disorder and Unspecified Internet Use Disorder in Adolescents: A Cluster Randomized Clinical Trial [J]. Journal of Behavioral Addictions, 2022, 11: 200-1.
[87] YANG W, HU W, MORITA N, et al. Impact of Short-Term Intensive-Type Cognitive Behavioral Therapy Intervention on Internet Addiction among Chinese College Students: A Randomized Controlled Trial [J]. International Journal of Environmental Research and Public Health, 2022, 19(9).
[88] SUN JIJUN, ZHU CHUNYAN, HU NANNAN, et al. Efficacy of High-Frequency Repetitive Transcranial Magnetic Stimulation on the Left Dorsolateral Prefrontal Cortex for Adolescent Online Gaming Disorder [J]. Journal of Clinical Psychiatry, 2022, 32(4).
[89] ZHAO YUXIA, ZHANG HAN. The Intervention Effect of Acupuncture on Psychological Craving in Internet Addiction Patients and Its Impact on Plasma DA, β-EP [J]. Henan Traditional Chinese Medicine, 2022, 42(2).
[90] ZHANG X, YANG H, ZHANG K, et al. Effects of Exercise or Tai Chi on Internet Addiction in College Students and the Potential Role of Gut Microbiota: A Randomized Controlled Trial [J]. Journal of Affective Disorders, 2023, 327: 404-15.
