# Supplementary material for: Optimal Non-Pharmacological Interventions for Reducing Problematic Internet Use in Youth: A Systematic Review and Bayesian Network Meta-Analysis
Source: Behav Sci (Basel). 2025 Jan 20;15(1):98. doi: 10.3390/bs15010098 (PMC11762159; doi:10.3390/bs15010098)
Supplement: Supplementary file 1 [file behavsci-15-00098-s001.zip › Supplement Appendix S5. Author contribution.pdf]

Jing-Jing Tian served as the main author and had full access to all data in the study. She was responsible for analyzing and interpreting the data, as well as analyzing the data acquisition, and contributed to the drafting of the manuscript. Yue-Shuai Jiang contributed to the study's conception and design. Zhen-Guo contributed to the critical revision of the manuscript for important intellectual content.
