# Supplementary material for: Optimal Non-Pharmacological Interventions for Reducing Problematic Internet Use in Youth: A Systematic Review and Bayesian Network Meta-Analysis
Source: Behav Sci (Basel). 2025 Jan 20;15(1):98. doi: 10.3390/bs15010098 (PMC11762159; doi:10.3390/bs15010098)
Supplement: Supplementary file 1 [file behavsci-15-00098-s001.zip › Supplement figure S2. Risk of bias graph.pdf]

Author, year	Item						
Song et al, 2020	L	U	U	U	H	U	U
Fateme et al, 2017	L	U	U	U	U	L	U
Fang and Dai, 2019	L	U	U	U	L	L	U
Ye et al, 2010	L	U	U	H	L	L	U
Qutaiba et al, 2021	L	U	U	U	L	U	L
Cao et al, 2007	L	U	U	H	L	U	U
Alavi et al, 2020	L	L	U	U	L	U	U
Chen et al, 2018	L	L	U	U	L	U	U
Du et al , 2010	L	L	U	H	L	U	U
Lindenberg et al, 2022	L	L	L	H	L	L	L
Wang, 2012	L	U	U	U	L	U	H
Wölfling et al, 2019	L	L	L	U	L	L	L
Yang et al, 2022	L	U	U	U	L	L	L
Liu et al, 2013	L	U	U	U	L	L	U
Li Geng, 2009	L	L	U	U	L	U	L
Li and Dai, 2009	L	L	U	U	L	U	L
Lu et al,2021	L	L	U	U	L	L	U
Ming, 2014	L	U	U	H	L	L	U
Wang et al,2011	L	L	U	H	L	U	L
Zhao et al,2016	L	U	U	U	L	L	U
Bong et al, 2020	L	U	L	U	L	U	U
Hong et al, 2020	L	U	L	U	L	L	U
Sung et al, 2016	L	U	L	U	U	L	L
Wei et al, 2021	L	U	U	U	L	L	U
Yang et al, 2017	L	L	U	U	L	U	L
Zhu et al, 2012	L	U	U	U	L	L	U
Tang et al, 2018	L	L	U	U	H	U	U
Tang et al, 2017	L	L	U	U	U	L	H
Yang, 2013	L	L	U	U	U	L	L
Zhu et al, 2008	L	L	U	H	L	L	L
Zhu et al, 2011	L	U	U	H	L	L	U
Zhu et al, 2011	L	L	U	H	L	U	L
Zhu et al, 2009	L	L	U	U	L	L	L
Çelik,2016	L	L	U	H	L	L	U

Abdullah, 2021	L	L	U	U	U	L	H
Yang, 2021	L	H	U	U	U	U	H
Zhang, 2013	L	U	U	U	L	L	U
Liao et al, 2009	L	U	U	H	L	U	L
Qiu et al, 2011	L	L	U	U	L	U	H
Yang et al,2017	L	U	U	U	U	H	H
Zhang et al, 2023	L	L	U	U	L	L	L
Ren et al,2014	L	H	U	U	L	H	U
Gao et al, 2012	L	U	U	U	L	L	L
Zhang, 2009	L	U	U	U	L	L	U
Bai et al, 2007	L	U	L	U	L	L	L
Huang et al, 2010	L	L	U	U	L	U	L
Li et al, 2017	L	L	U	H	L	U	H
Zhang and Wang, 2011	L	U	U	U	U	H	U
Yin, 2021	L	U	U	U	L	U	H
Zhang, 2011	L	H	H	U	U	U	H
Chen et al,2010	L	L	U	U	L	L	U
Chen et al,2011	L	L	U	U	L	U	U
Duan et al,2012	L	U	H	U	L	U	H
Ge et al,2014	L	U	U	H	U	L	U
Guo et al,2008	L	L	U	H	L	L	U
Liu,2014	L	L	U	U	L	L	U
Liu et al,2010	L	L	U	U	L	U	L
Lv et al,2012	L	L	U	U	L	U	L
Park and Hee, 2011	L	L	U	H	H	U	H
Zhang et al,2009	L	L	U	U	L	U	H
Zheng et al,2007	L	L	U	U	L	L	U
Guan et al,2012	L	L	U	U	L	U	U
Kong et al,2011	L	L	U	U	H	L	U
Li, 2012	L	U	U	U	H	U	U
Su,2015	L	L	U	U	U	L	U
Liu et al,2013	L	U	U	U	L	L	U
Peng et al,2015	L	L	U	U	U	L	U
Xu et al,2012	L	U	U	U	L	U	L
Xu et al,2018	L	L	U	U	L	L	U
Zhang and Wang, 2015	L	L	U	U	U	U	H

Study	Random sequence generation (selection bias)	Allocation concealment(selection bias)	Blinding of participants and personnel (performance bias)	Blinding of outcome assessment (detection bias)	Incomplete outcome data (attrition bias)	Selective reporting (reporting bias)	Other bias
Zhao and Hu, 2015	L	U	U	U	H	H	U
Zhong et al, 2011	L	L	U	U	L	L	L
Lu et al, 2013	L	L	U	U	L	L	U
Du et al, 2013	L	U	U	H	U	H	H
Wei et al,2014	L	L	U	H	U	U	U
Liu et al,2015	L	L	U	U	L	L	U
Gong et al,2022	L	U	U	H	L	U	U
Preeti et al, 2020	L	U	U	H	H	L	U
Zhao and Zhang, 2022	L	L	U	U	L	U	U
Li et al, 2017	L	L	U	U	U	H	U
Liu et al, 2021	L	L	U	U	L	L	U
Huang, 2020	L	L	U	U	U	L	U
Cheng and luo, 2017	L	L	U	U	L	L	L
Jeong et al, 2020	L	L	U	U	L	U	U
Lee et al, 2021	L	L	L	H	L	L	L
Zhong, 2020	L	U	U	U	L	L	U
Sun et al, 2022	L	L	U	U	U	L	U
Zhang et al, 2018	L	U	U	U	L	L	U
Kim, 2008	L	U	U	U	L	U	H
Deng et al, 2017	L	U	U	U	U	H	U

Low risk

Unclear risk

High risk
