# Supplementary material for: Optimal Non-Pharmacological Interventions for Reducing Problematic Internet Use in Youth: A Systematic Review and Bayesian Network Meta-Analysis
Source: Behav Sci (Basel). 2025 Jan 20;15(1):98. doi: 10.3390/bs15010098 (PMC11762159; doi:10.3390/bs15010098)
Supplement: Supplementary file 1 [file behavsci-15-00098-s001.zip › Supplement table S1. Inclusion and Exclusion criteria.pdf]

Supplement Table S1. Inclusion and Exclusion criteria

PICOS	Inclusion criteria	Exclusion criteria
P<br>Population	Participants ranged from 15 to 24 years old with IA, diagnosed by Young Diagnostic Questionnaire, Revised Chen Internet Addiction Scale (CIAS-R), or other IA scales	1) Youth were currently receiving any form of non-drug intervention<br>2) If most enrolled patients were substance withdrawal.
I<br>Intervention	Interventions included any manualized or structured non-pharmacological interventions: sports intervention (SI), electroencephalogram biological feedback (EBF), reality therapy (RT), positive psychology therapy (PPT), sandplay therapy (ST), educational intervention (EI), compound psychotherapy (CPT), electroacupuncture therapy (AT), group counseling (GC), family therapy (FT), electrotherapy (ELT), craving behavior intervention (CBI), virtual reality therapy (VRT), cognitive behavior therapy (CBT), mindfulness therapy (MT)	Any study that combines or compares with a pharmacological.
C<br>Comparator	Comparators included another class of Non-pharmacological intervention or a control condition, such as waitlist, treatment-as-usual, or named control group.	Placebo or control group in any combination with any intervention.
O<br>Outcome	The outcome was the mean overall change in internet addiction symptoms from baseline to endpoint	Clinical assessment scale or other relevant outcome can not evaluate the change in internet addiction symptom for each included studies.
S<br>Study design	Randomized controlled trials, including cross-over and cluster randomized trials, were selected without any restrictions on the year of publication or language.	Randomized controlled trials, including cross-over and cluster randomized trials, were selected without any restrictions on the year of publication or language.

T (Time stamp and database）	Cochrane Central Register of Controlled Trials, Embase, Medline, Web of Science, China National Knowledge Infrastructure, China Science and Technology Journal Database, Chinese BioMedical Literature Database, and WanFang Data were used to perform an exhaustive search to identify relevant studies from their inception to December 22, 2023, in accordance with the Cochrane Handbook for Systematic Reviews of Interventions.
