# Supplementary material for: Optimal Non-Pharmacological Interventions for Reducing Problematic Internet Use in Youth: A Systematic Review and Bayesian Network Meta-Analysis
Source: Behav Sci (Basel). 2025 Jan 20;15(1):98. doi: 10.3390/bs15010098 (PMC11762159; doi:10.3390/bs15010098)
Supplement: Supplementary file 1 [file behavsci-15-00098-s001.zip › Supplement table S2. Description of NPIs.pdf]

Supplement Table S2. Description of non-pharmacological intervention

	Type of non-pharmacological intervention	Abbreviation	Inclusion	Goals	Measure
**Active group**	Acupuncture therapy[1]	AT	Acupuncture；<br>Moxibustion.	Acupuncture for internet addiction seeks to holistically regulate brain chemistry, reduce cravings, alleviate withdrawal symptoms, improve cognitive functions, and support overall well-being, while emphasizing the importance of family and community involvement for effective treatment outcomes.	Acupuncture treatment for internet addiction involves specific measures such as needling to regulate neurobiological processes, reduce compulsive internet use, alleviate withdrawal symptoms, and improve cognitive function.
	Cognitive behavior therapy[2]	CBT	Behavioral therapy;<br>Cognitive therapy;<br>Cognitive behavioral therapy;<br>Group cognitive behavioral therapy;<br>Individual cognitive behavioral therapy.	CBT is a therapeutic approach that focuses on the assessment, confrontation, and alteration of a patient's maladaptive cognitive schemas, with the ultimate goal of facilitating positive behavioral transformations.	Cognitive Behavioral Therapy (CBT) traditionally necessitates direct, interpersonal engagement between the patient and the therapist, wherein cognitive restructuring techniques are employed to facilitate the reevaluation and modification of maladaptive thought patterns. This process is instrumental in promoting behavioral change and enhancing the overall therapeutic efficacy.
	Compound Psychotherapy[3]	CPT	Narrative therapy in combination with Pilates exercise;<br>Group behavioral therapy combined with motivational interviewing and life skills practices.	Mixed psychological therapy aims to provide a comprehensive and personalized approach to mental health treatment by integrating various therapeutic techniques to alleviate symptoms and enhance overall well-being.	Mixed psychological therapy is based on psychological interventions and aims to alleviate the symptoms of participants by integrating other therapeutic approaches.
	Craving behavior intervention[4]	CBI	Motivational interviewing；<br>Exposure and response prevention；<br>Acceptance and commitment therapy.	The goal of craving behavior intervention is to help individuals reduce their cravings for addictive substances or behaviors, enhance self-control, and thereby promote long-term behavioral change and a healthy lifestyle through psychological strategies and behavioral techniques.	Craving behavior therapy employs specific strategies like cognitive restructuring, mindfulness, and exposure techniques to help individuals manage and overcome their addictive cravings.

Educational intervention[5]	EI	Cognitive behavioral education; Self-Management skills training; Emotional regulation education.	The goal of educational intervention is to equip individuals with knowledge, skills, and support to foster healthy behavioral patterns, enhance problem-solving abilities, and prevent or mitigate negative behaviors such as internet addiction.	Educational interventions employ a mix of didactic instruction, interactive learning, skill-building workshops, multimedia resources, case studies, peer education, community engagement, and continuous support to effectively impart knowledge and foster behavioral change.
Electroencephalogram biological feedback[6]	EBF	EEG Sensorimotor Rhythm Training;<br>Electromyography (EMG) Biofeedback;<br>Skin Temperature Feedback.	EBF aims to train individuals to voluntarily regulate their brainwave patterns to improve cognitive functioning and emotional self-regulation, thereby enhancing overall mental health and well-being.	EBF involves converting an individual's physiological signals, such as EEG, EMG, or GSR, into real-time audio-visual feedback, guiding the individual to learn to control these physiological processes through psychological and behavioral training to achieve therapeutic and health improvement goals.
Mindfulness therapy[7]	MT	Mindfulness based stress reduction;<br>Mindfulness based cognitive therapy.	The objective is to reconfigure the interplay between symptomatic experiences and the associated cognitive and emotional challenges, thereby fostering a more adaptive and constructive relationship between these elements.	MT is designed to fortify attentional capacities and augment the capability to suppress cognitive and emotional responses. This intervention is posited to target and potentially ameliorate the precipitating factors of depressive symptomatology.
Positive psychology[8]	PPT	Acceptance and Commitment Therapy;<br>Humanistic Psychotherapy;<br>Positive Psychological Education.	PPT aims to foster well-being and life satisfaction by enhancing individual happiness, strengths, personal growth, resilience, interpersonal relationships, mental health, meaning and purpose in life, and cross-cultural understanding.	Positive psychology employs specific operational measures such as identifying and nurturing individual strengths, promoting positive emotions, fostering gratitude and mindfulness, setting and pursuing meaningful goals, encouraging optimistic thinking, and building strong social connections.
Reality therapy[9]	RT	Maladaptive Behaviors;<br>Adaptive Behaviors;<br>Internal Control;<br>External Control.	Reality Therapy aims to empower individuals to take responsibility for their actions, make effective choices, and satisfy their basic needs in a way that leads to a more fulfilling and successful life.	Reality therapy uses the WDEP model (Wants, Doing, Evaluation, Planning) as an operational framework to guide individuals to focus on currently controllable behaviors, set specific goals, refuse excuses, take action, and be responsible for their choices and actions, in order to achieve personal goals and enhance life satisfaction.

Sandplay therapy[10]	ST			
Sports intervention[11]	SI	Exercise;<br>Physical activity;<br>Sports;	To relieve internet addition by relaxing youth’s body and mind through any form of physical activity.	SI should be tailored to accommodate the individual's schedule, personal preferences, and physical fitness capabilities, thereby ensuring adaptability to various contexts. Moreover, such interventions are designed to be implemented in a wide range of settings with minimal risk of adverse effects.
Virtual reality therapy[12]	VRT	Virtual Reality Exposure Therapy	VRT aims to provide immersive treatment experiences that help individuals confront and manage psychological issues such as phobias, anxiety, PTSD, and addiction through controlled exposure and skill-building in a safe virtual environment.	VRT operates by using computer-generated environments to simulate real-world situations, allowing individuals to engage in controlled exposure to fearful stimuli, practice coping strategies, and receive immediate feedback from therapists, all within an interactive and immersive virtual space.
Electroacupuncture therapy[13]	ELT	Standard Electroacupuncture Therapy;<br>Electroacupuncture for Post-Surgical Pain;<br>Electroacupuncture at Specific Acupuncture Points	Electroacupuncture therapy aims to enhance the therapeutic effects of traditional acupuncture by integrating electrical stimulation, effectively treating a broad spectrum of conditions ranging from pain management to various systemic disorders, and is now widely applied in the treatment of various addictions.	Electroacupuncture therapy involves the strategic insertion of acupuncture needles at specific acupoints, followed by the application of electrical currents to modulate the intensity and frequency of stimulation, targeting a variety of health conditions and promoting the body's self-healing capabilities.
Group counselling[14]	GC	Psychoanalytic Group Counseling;<br>Behaviorist Group Counseling;<br>Interpersonal Group Counseling.	Group counseling aims to provide a supportive environment for individuals to explore and address personal issues, develop social skills, gain insights, and foster mutual support and understanding within a shared community.	Group counseling involves structured activities, open discussions, role-playing, feedback, and guided reflection led by trained professionals to facilitate personal growth, interpersonal skill development, and emotional support among members.
Family therapy[15]	FT	Family group cognitive–behavioral intervention;<br>Attachment Based Family Therapy; Family-focused treatment.	Focusing primarily on improving internet addiction symptom as a function of this<br>improved relationship.	FT tends to view change in terms of the systems of interaction between family members that is strengthen the connection between the teen and their caregiver through relationship building activities.

**Control group**	No intervention	NI	A control condition	Participants in the study are not administered any active therapeutic intervention during the course of the research; however, they are duly informed that they may be eligible to receive such treatment upon the conclusion of the study period.
	Routine intervention	RI	A control condition	The nomenclature RI commonly denotes an array of standardized therapeutic modalities accessible to the particular demographic under consideration.

Reference

[1]Ernst E. Acupuncture–a critical analysis[J]. Journal of internal medicine, 2006, 259(2): 125-137.

[2]Christ C, Schouten M J E, Blankers M, et al. Internet and computer-based cognitive behavioral therapy for anxiety and depression in adolescents and young adults: systematic review and meta-analysis[J]. Journal of medical Internet research, 2020, 22(9): e17831.

[3]Xu L, Wu L, Geng X, et al. A review of psychological interventions for internet addiction[J]. Psychiatry Research, 2021, 302: 114016.

[4]Zhang J T, Yao Y W, Potenza M N, et al. Altered resting-state neural activity and changes following a craving behavioral intervention for Internet gaming disorder[J]. Scientific Reports, 2016, 6(1): 28109.

[5]Çelik Ç B. Educational Intervention for Reducing Internet Addiction Tendencies[J]. Addicta: The Turkish Journal on Addictions, 2016, 3(3).

[6]Demin D, Poskotinova L. Neurophysiologic reactions during heart rate variability biofeedback session in adolescents with different risk of internet addiction[J]. International Journal of Environmental Research and Public Health, 2022, 19(5): 2759.

[7]Shadbad N R. The Effectiveness of Mindfulness-Based Group Therapy on Reducing Internet Addiction and Increasing the General Health of Adolescent Girls[J]. Indian Journal of Public Health Research & Development, 2017, 8(4).

[8]Khazaei F, Khazaei O, Ghanbari-H B. Positive psychology interventions for internet addiction treatment[J]. Computers in Human Behavior, 2017, 72: 304-311.

[9]Safari S, Soleimanian A A, Jajarmi M. The effectiveness of" reality therapy" training on internet addiction and perceptions of social support-family in teenage girls[J]. Journal of Health Promotion Management, 2021, 10(5): 107-120.

[10]Shin J H, Jang M. Effect of group sandplay therapy to be addicted youth’ s addiction levels and anxiety[J]. Journal of Symbols & Sandplay Therapy, 2016, 7(1): 39-55.

[11]Zhou Z, Li C, Yuan J, et al. Effectiveness of sports intervention: A meta-analysis of the effects of different interventions on adolescent internet addiction[J]. Journal of Affective Disorders, 2024.

[12]Park S Y, Kim S M, Roh S, et al. The effects of a virtual reality treatment program for online gaming addiction[J]. Computer methods and programs in biomedicine, 2016, 129: 99-108.

[13]Yang Y, Li H, Chen X, et al. Electro-acupuncture treatment for internet addiction: Evidence of normalization of impulse control disorder in adolescents[J]. Chinese Journal of Integrative Medicine, 2017, 23(11): 837-844.

[14]Fadil M, Sunawan S, Awalya A. The effectiveness of group counselling with cognitive restructuring and self-management techniques to reduce internet addiction[J]. Jurnal Bimbingan Konseling, 2019, 8(2): 134-139.

[15]Liu Q X, Fang X Y, Yan N, et al. Multi-family group therapy for adolescent Internet addiction: Exploring the underlying mechanisms[J]. Addictive Behaviors, 2015, 42: 1-8.
