# Supplementary material for: Optimal Non-Pharmacological Interventions for Reducing Problematic Internet Use in Youth: A Systematic Review and Bayesian Network Meta-Analysis
Source: Behav Sci (Basel). 2025 Jan 20;15(1):98. doi: 10.3390/bs15010098 (PMC11762159; doi:10.3390/bs15010098)
Supplement: Supplementary file 1 [file behavsci-15-00098-s001.zip › Supplement table S3. Demographic characteristics.pdf]

Table S3. Characteristics of the included studies

Author/year	Sample size		Location (Language)	Type of intervention		Outcome measurements	Intervention duration
	Intervention gtoup	Control group		Intervention group	Control group		
Song et al, 2020	60	60	Henan, China (Chinese)	PPT	RI	CIAS-R	20weeks
Fateme et al, 2017	24	24	Iran (English)	PPT	NI	YIAT	7days
Fang and Dai, 2019	24	24	Liaoning, China (Chinese)	PPT	NI	YDQ	8weeks
Ye et al, 2010	39	CG1:36<br>CG2:37	Taiwan, China (Chinese)	CBT	CG1:AT<br>CG2:CPT	YIAT	40days
Qutaiba et al, 2021	80	80	Israel (English)	CBT	RI	YIAT	12weeks
Cao et al, 2007	29	35	Henan, China (Chinese)	CBT	RI	YDQ	8weeks
Alavi et al, 2020	18	23	Iran (English)	CBT	RI	YIAT	15weeks
Chen et al, 2018	52	52	Fujian, China (Chinese)	CBT	RI	CIAS-R	NR
Du et al, 2010	32	24	Shanghai, China (Chinese)	CBT	NI	IOSRS	9weeks
Lindenberg et al, 2022	167	256	Germany (English)	CBT	NI	CIUS	12months
Wang, 2012	32	32	Hubei, China (Chinese)	CBT	RI	YDQ	3months
Wölfling et al, 2019	72	71	Germany and Austria	CBT	NI	AICA-S	15weeks
Yang et al, 2022	21	22	Yunnan, China (Chinese)	CBT	NI	YIAT	6weeks
Liu et al, 2013	16	15	Shenzhen, China (Chinese)	CBT	RI	YIAT	12weeks
Li Geng, 2009	48	48	Ningxia, China (Chinese)	CBT	NI	YDQ	8weeks
Li and Dai, 2009	38	38	Ningxia, China (Chinese)	CBT	NI	CIAS	8weeks
Lu et al, 2021	16	17	Guizhou, China (Chinese)	CBT	NI	YIAT	5weeks
Ming, 2014	60	60	Shandong, China (Chinese)	CBT	RI	YIAT	40weeks
Wang et al, 2011	36	34	Ningxia, China (Chinese)	CBT	RI	CIAS	2months
Zhao et al, 2016	65	65	Shandong, China (Chinese)	CBT	RI	CIAS	16weeks
Bong et al, 2020	67	71	Korea (English)	CBT	CPT	YIAT	8weeks
Hong et al, 2020	25	25	Korea (English)	CBT	CPT	YIAT	14weeks
Sung et al, 2016	12	12	Korea (English)	CBT	VRT	YIAT	8weeks

Wei et al, 2021	39	CG1:36<br>CG2:37	Taiwan, China<br>(Chinese)	CBT	CG1:AT<br>CG2:CPT	YIAT	40days
Yang et al, 2017	16	16	Sichuan, China<br>(Chinese)	CBT	AT	YIAT	45days
Zhu et al, 2012	40	CG1:40<br>CG2:40	Sichuan, China<br>(Chinese)	CBT	CG1:AT<br>CG2:CPT	YIAT	40days
Tang et al, 2018	23	CG1:20<br>CG2:21	Hunan, China<br>(Chinese)	CBT	CG1:AT<br>CG2:CPT	YIAT	4weeks
Tang et al, 2017	20	CG1:23<br>CG2:21	Hunan, China<br>(Chinese)	CBT	CG1:AT<br>CG2:CPT	YIAT	4weeks
Yang, 2013	14	15	Sichuan, China<br>(Chinese)	CBT	AT	YIAT	40days
Zhu et al, 2008	22	23	Sichuan, China<br>(Chinese)	CBT	CPT	YIAT	40days
Zhu et al, 2011	36	CG1:39<br>CG2:37	Sichuan, China<br>(Chinese)	CBT	CG1:AT<br>CG2:CPT	YIAT	40days
Zhu et al, 2011	36	CG1:39<br>CG2:37	Sichuan, China<br>(Chinese)	CBT	CG1:AT<br>CG2:CPT	YIAT	40days
Zhu et al, 2009	22	23	Sichuan, China<br>(Chinese)	CBT	CPT	YIAT	40days
Çelik, 2016	15	15	TURKISH<br>(English)	EI	NI	PIUS	5weeks
Abdullah, 2021	15	15	TURKISH<br>(English)	EI	NI	YIAT	6weeks
Yang, 2021	56	28	Hunan, China<br>(Chinese)	SI	NI	CIAS	12weeks
Zhang, 2013	30	30	Yunnan, China<br>(Chinese)	SI	NI	YIAT	4months
Liao et al, 2009	30	30	Hunan, China<br>(Chinese)	SI	NI	CIAS	10weeks
Qiu et al, 2011	18	18	Shandong, China<br>(Chinese)	SI	NI	YDQ	12weeks
Yang et al, 2017	26	26	Fujian, China<br>(Chinese)	SI	NI	CIAS	16weeks
Zhang et al, 2023	62	31	Anhui, China<br>(Chinese)	SI	NI	YIAT	8weeks
Ren et al, 2014	4	4	Heilongjiang, China<br>(Chinese)	SI	NI	YIAT	12weeks
Gao et al, 2012	35	34	Jilin, China<br>(Chinese)	SI	NI	YIAT	8weeks
Zhang, 2009	35	35	Xian, China<br>(Chinese)	SI	GC	CIAS	12weeks
Bai et al, 2007	24	24	Beijing and<br>Sichuan, China	GC	NI	CIAS	6weeks

			(Chinese)				
Huang et al, 2010	17	10	Beijing, China (Chinese)	GC	NI	CGAI	3weeks
Li et al, 2017	30	30	Guangdong, China (Chinese)	GC	NI	YDQ	8weeks
Zhang and Wang, 2011	18	27	Jilin, China (Chinese)	GC	NI	QIA	4months
Yin, 2021	30	30	Chongqing, China (Chinese)	GC	NI	APIUS	1.5month
Zhang, 2011	6	6	Sichuan, China (Chinese)	GC	NI	DSIADC	NR
Chen et al, 2010	30	31	Wenzhou, China (Chinese)	GC	NI	YDQ	8weeks
Chen et al, 2011	14	23	Chongqing, China (Chinese)	GC	NI	CIAS	7weeks
Duan et al, 2012	11	11	Hunan, China (Chinese)	GC	NI	YIAT	3months
Ge et al, 2014	12	12	Chongqing, China (Chinese)	GC	NI	CIAS	5weeks
Guo et al, 2008	16	16	Nanchang, China (Chinese)	GC	RI	CIAS	1month
Liu, 2014	12	12	Fujian, China (Chinese)	GC	NI	YIAT	8weeks
Liu et al, 2010	80	80	Jiangsu, China (Chinese)	GC	RI	YIAT	4weeks
Lv et al, 2012	26	26	Shandong, China (Chinese)	GC	RI	CIAS	8weeks
Park and Hee, 2011	18	19	Korea (English)	GC	NI	KIAS	5weeks
Zhang et al, 2009	12	13	Jiangsu, China (Chinese)	GC	NI	YDQ	8weeks
Zheng et al, 2007	30	31	Wenzhou, China (Chinese)	GC	NI	YDQ	9weeks
Guan et al, 2012	25	25	Jiangsu, China (Chinese)	GC	RI	CIAS	4weeks
Kong et al, 2011	36	35	Jiangsu, China (Chinese)	GC	NI	YIAT	8weeks
Li, 2012	27	25	Shandong, China (Chinese)	GC	NI	IADDS	4months
Su, 2015	15	15	Henan, China (Chinese)	GC	NI	CIAS	6weeks
Liu et al, 2013	16	15	Shenzhen, China (Chinese)	GC	RI	YIAT	13weeks
Peng et al, 2015	28	28	Gansu, China	GC	NI	YDQ	8weeks

			(Chinese)				
Xu et al, 2012	13	13	Jiangxi, China (Chinese)	GC	NI	YDQ	12weeks
Xu et al, 2018	20	20	Hubei, China (Chinese)	GC	RI	CIAS	1month
Zhang and Wang, 2015	10	CG1:10 CG2:10	Guangdong, China (Chinese)	GC	CG1:SI CG2:ST	YIAT	4monthss
Zhao and Hu, 2015	24	24	Jilin, China (Chinese)	GC	NI	CIAS	4weeks
Zhong et al, 2011	28	29	Beijing, China (Chinese)	FT	GC	OCS	14weeks
Lu et al, 2013	60	60	Shandong, China (Chinese)	FT	CPT	IAD	5months
Du et al, 2013	30	33	Shandong, China (Chinese)	FT	RI	YDQ	4months
Wei et al, 2014	60	60	Shandong, China (Chinese)	FT	RI	YIAT	20weeks
Liu et al, 2015	21	25	Neimenggu, China (Chinese)	FT	NI	PIUS	18days
Gong et al, 2022	60	58	Hunan, China (Chinese)	CPT	NI	YDQ	8weeks
Preeti et al, 2020	30	30	India (English)	CPT	NI	PIU	12weeks
Zhao and Zhang, 2022	40	20	Henan, China (Chinese)	AT	NI	YIAT	10weeks
Li et al, 2017	15	15	America (English)	MT	RI	DSM-5 Criteria for IGD	8weeks
Liu et al, 2021	60	61	Shandong, China (Chinese)	MT	RI	CIAS	8weeks
Huang, 2020	34	34	Chongqing, China (Chinese)	MT	NI	APIUS	9weeks
Cheng and luo, 2017	25	25	Jiangxi, China (Chinese)	EBF	GC	YIAT	12weeks
Jeong et al, 2020	13	13	Korea (English)	ELT	sham tDCS	YIAT	4weeks
Lee et al, 2021	14	12	Korea (English)	ELT	sham tDCS	YIAT	5days
Zhong, 2020	32	32	China (Chinese)	ELT	sham tDCS	YIAT	4weeks
Sun et al, 2022	30	31	China (Chinese)	ELT	sham rTMS	CIAS	2weeks
Zhang et al, 2018	33	33	China (Chinese)	ST	RI	YIAT	12weeks
Kim, 2008	13	12	Korea ((English))	RT	NI	K-IAS	5weeks
Deng et al, 2017	44	19	China (Chinese)	CBI	NI	CIAS	6weeks

APIUS, Adolescent pathological internet use scale; AICA-S, Assessment of internet and computer game addiction self-report; ELT, Electroacupuncture therapy; CIAS-R, Revised chen internet addiction scale; CIUS, Compulsive Internet use scale; CIAS, Chen internet addiction scale; CGAI, Computer gaming addiction invention; CBT, Cognitive behavior therapy; CPT, Compound Psychotherapy; DSIADC, Discriminating scale on internet addiction disorder of callan; EI, Educational intervention; EBF, Electroencephalogram biological feedback; FT,Family therapy; GC, Group counselling; IGD, Internet gaming disorder; IAD, Internet addiction disorder;

IOSRS, Internet overuse self-rating scale; IADDS, Internet addiction disorder diagnostic scale; MT, Mindfulness therapy; NI, No intervention; K-IAS, Korea internet addiction scale; PPT, Positive psychology; PIUS, Problematic internet use scale;QIA, Questionnaire for internet addiction; RT, Reality therapy; RI, Routine intervention; ST, Sandplay therapy; SI, Sports intervention; VRT, Virtual reality therapy; YDQ, Young diagnostics questionnaire; YIAT, Young internet addiction test.
