# Supplementary figures and images for: Optimal Non-Pharmacological Interventions for Reducing Problematic Internet Use in Youth: A Systematic Review and Bayesian Network Meta-Analysis
Source: Behav Sci (Basel). 2025 Jan 20;15(1):98. doi: 10.3390/bs15010098 (PMC11762159; doi:10.3390/bs15010098)

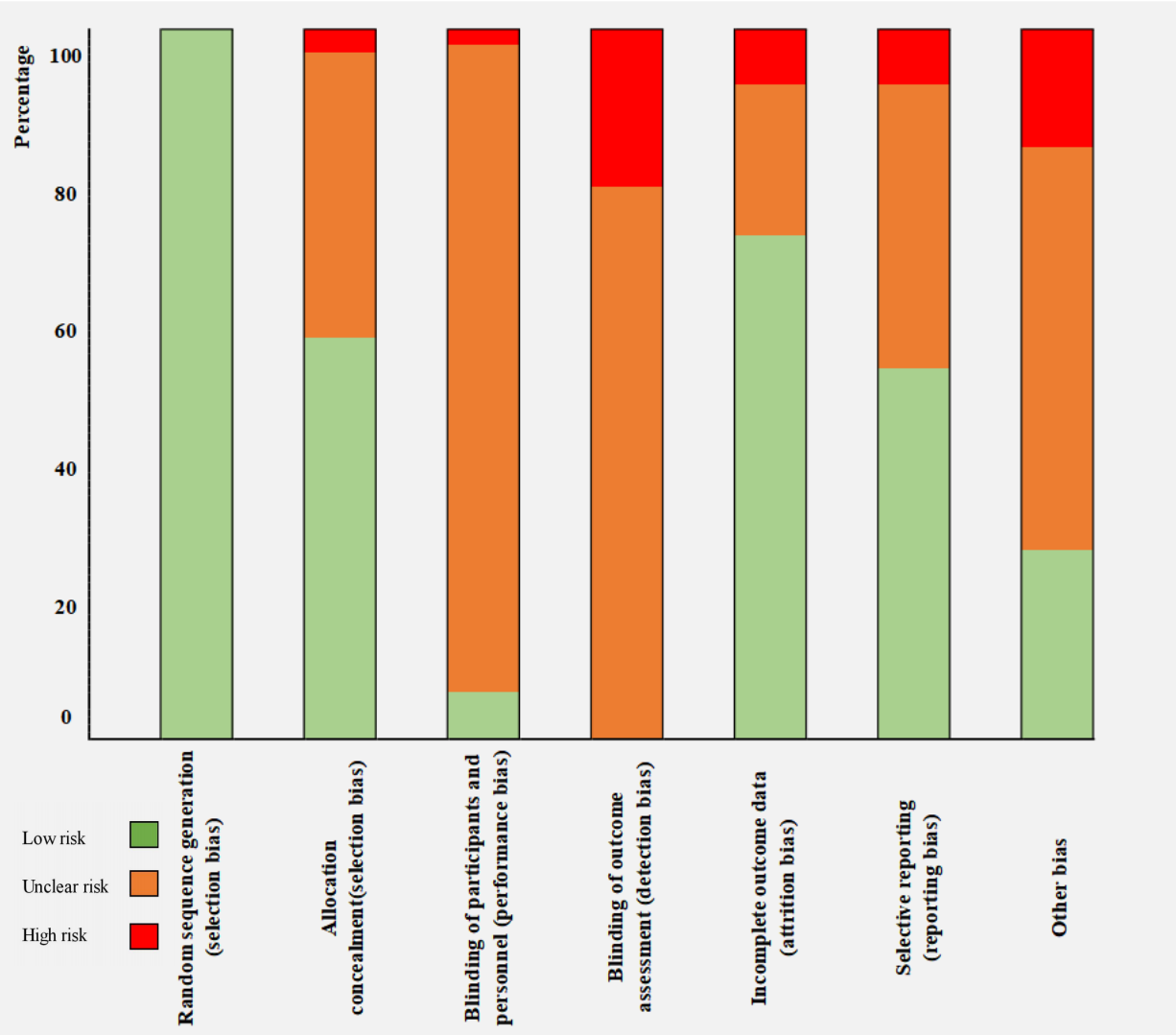

Supplement: Supplementary file 1 [file behavsci-15-00098-s001.zip › Supplement figure S1. Risk of bias summary.pdf]

Funnel plot with pseudo 95% confidence limits

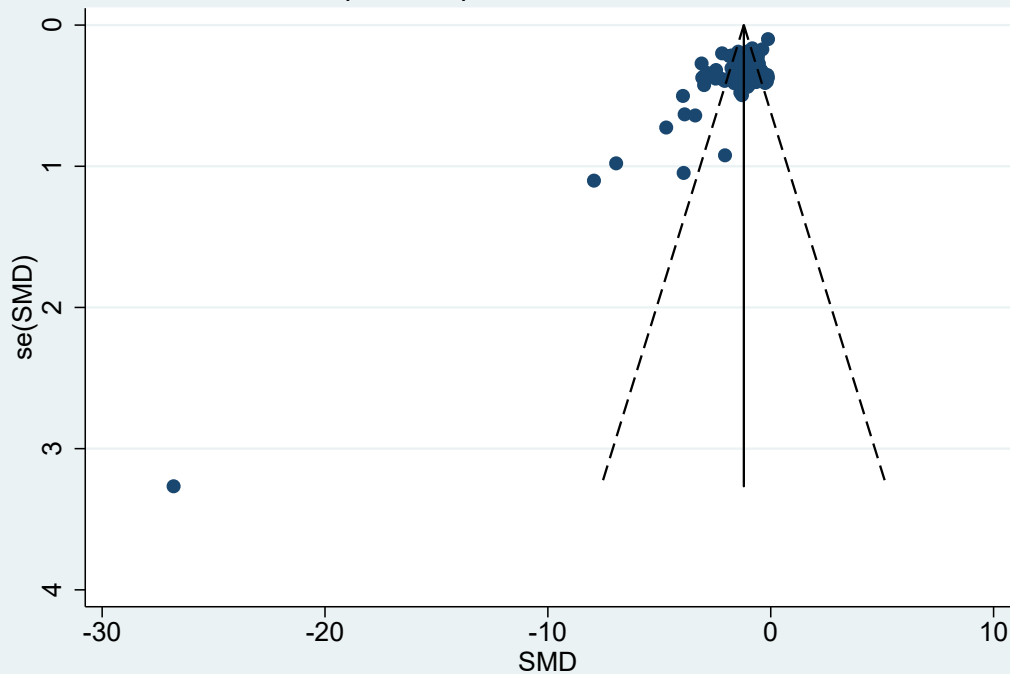

Supplement: Supplementary file 1 [file behavsci-15-00098-s001.zip › Supplement figure S3. Funnel plot.pdf]

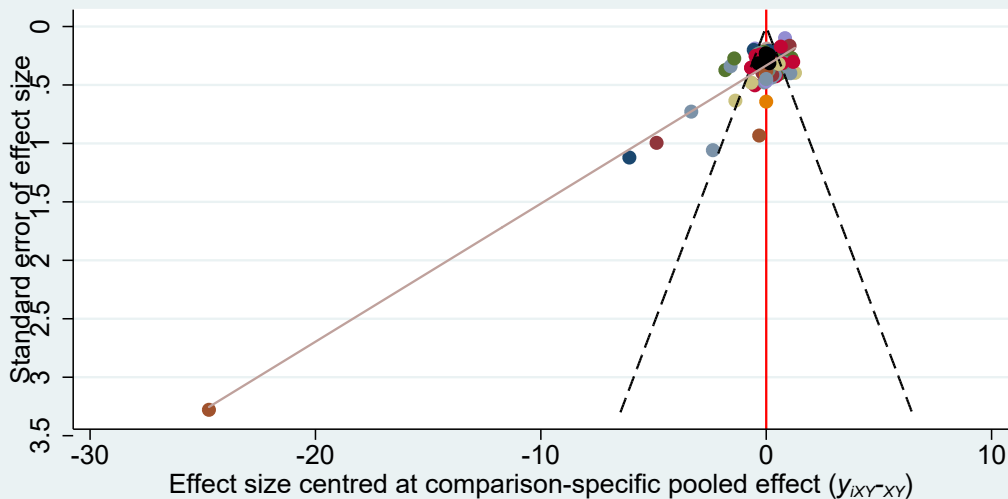

Supplement: Supplementary file 1 [file behavsci-15-00098-s001.zip › Supplement figure S4. Funnel plot.pdf]
